# The synthesis of the 2,3-difluorobutan-1,4-diol diastereomers

**DOI:** 10.3762/bjoc.13.280

**Published:** 2017-12-27

**Authors:** Robert Szpera, Nadia Kovalenko, Kalaiselvi Natarajan, Nina Paillard, Bruno Linclau

**Affiliations:** 1 Chemistry, University of Southampton, Highfield, Southampton SO17 1BJ, United Kingdom

**Keywords:** acetal isomerization, deoxyfluorination, epoxide opening, fluorinated building block, vicinal difluoride

## Abstract

The diastereoselective synthesis of fluorinated building blocks that contain chiral fluorine substituents is of interest. Here we describe optimisation efforts in the synthesis of *anti*-2,3-difluorobutane-1,4-diol, as well as the synthesis of the corresponding *syn*-diastereomer. Both targets were synthesised using an epoxide opening strategy.

## Introduction

The introduction of fluorine in organic compounds usually results in the modification of a range of chemical, physical and biological properties [1]. Fluorine incorporation is therefore a common strategy to optimise the properties of drugs/agrochemicals, as well as materials [2–6].

Many methods exist for the stereoselective introduction of the C–F group [7–11]. An alternative and often time-efficient approach is the use of fluorinated building blocks, where fluorine is introduced as part of a carbon containing fragment, sometimes also bearing other functionality [12–13]. The development of novel fluorinated building blocks is therefore of interest, particularly those that can be synthesised conveniently on a multigram scale. Interestingly, the majority of currently commercially available fluorinated building blocks do not contain stereogenic C–F bonds.

**Scheme 1:**
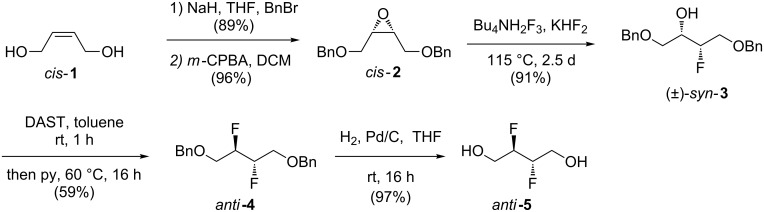
The synthesis of *anti-*2,3-difluorobutan-1,4-diol (*anti*-**5**) [17].

The *vicinal*-difluoride motif is known to exert conformational control through the fluorine *gauche* effect [14–15], and so building blocks containing this motif are of interest [16–17]. We have previously reported on the gram-scale synthesis of *meso*-2,3-difluorobutane-1,4-diol (*anti*-**5**) starting from commercially available *cis*-but-2-ene-1,4-diol (Scheme 1) [17]. The *vicinal*-difluoride group was introduced by a two-step sequence, with initial nucleophilic epoxide [18] opening by a fluoride source [19], followed by nucleophilic deoxyfluorination [9–11].

In this contribution, we report on work directed at the further optimisation of the synthesis of *anti*-**5**, as well as on a gram-scale synthesis of its diastereomer (±)-*syn-***5**, a novel compound.

## Results and Discussion

### Optimisation of the synthesis of *anti-***5**

While the synthesis of *anti-***5** as described in Scheme 1 was high-yielding [17], two disadvantages were apparent. First, the epoxide opening takes 2.5 days at 115 °C and uses an expensive fluoride source (Landini’s reagent [18]: Bu_4_NH_2_F_3_). It was found that Bu_4_NH_2_F_3_ made in-house gave significantly reduced yields. Second, the use of the benzyl ether protecting group resulted in a significant increase in mass, and therefore, chromatographic purification of the protected intermediates upon scale-up was inconvenient.

**Scheme 2:**
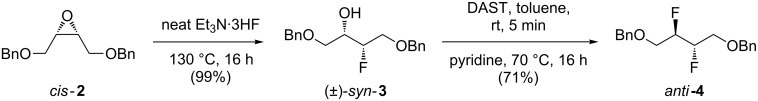
Improved epoxide opening and deoxofluorination conditions.

As previously reported [17], epoxide opening of *cis-***2** with Olah’s reagent (HF·py) led to an 80% yield of the fluorohydrin after just three hours, however, the product was obtained as a mixture of both the *syn-* and *anti*-diastereomers. Whilst no mechanistic studies were conducted, it is possible that competing S_N_1 and/or anchimeric assistance by the benzyloxy group occurred. Work by Schlosser has shown that 1,2-disubstituted epoxide opening with Et_3_N·3HF proceeds with excellent diastereoselectivity [19]. Et_3_N·3HF is less acidic than Olah’s reagent, disfavouring S_N_1 and rearrangement pathways [20–21]. Indeed, the use of this reagent for the epoxide opening of *cis*-**2** led to (*±*)-*syn-***3** in excellent yield (Scheme 2), with no significant isomerisation (see Supporting Information File 1). Epoxide opening with the recently described TBAF/KHF_2_ [22] was also possible, but in lower yield (75%, not shown). Incidentally, it was also found that the subsequent deoxofluorination reaction was somewhat higher yielding when DAST was added at rt over just 5 min, immediately followed by the addition of pyridine and heating at 70 °C.

It should be noted that DAST is known to undergo decomposition at temperatures above 90 °C [23]. Here we use DAST in solution. The initial mixing is at room temperature, and heating doesn’t exceed 70 °C, and therefore, the procedure is deemed to have low risk. Nonetheless, care must be taken and the reaction was run with the protection of a blast shield.

**Scheme 3:**
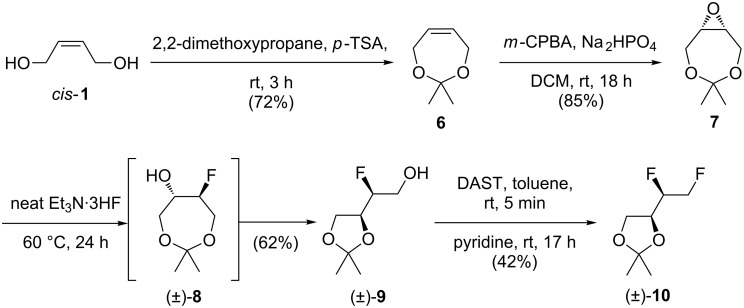
Attempted synthesis of *anti*-**5** via acetonide protection.

In order to reduce the relative contribution of the protecting group to the overall weight of the intermediates, the use of an acetonide was explored. Given the starting alkene was *cis-*configured, its introduction was possible from the start (Scheme 3).

Hence, following literature procedures [24–26], the reaction of *cis*-**1** with 2,2-dimethoxypropane and subsequent epoxidation led to **7**. However, epoxide opening with Et_3_N·3HF was accompanied by acetonide rearrangement to afford fluorohydrin (±)-**9**, containing the thermodynamically favoured five-membered ring [24]. This is clearly indicated by the appearance of a doublet of doublets for the primary alcohol OH proton. DAST-mediated deoxofluorination then led to (±)-**10**, in which an alkyl fluoride signal at −232 ppm confirmed the presence of a primary fluoride.

**Scheme 4:**
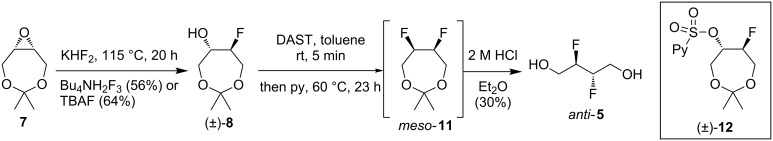
Completion of the synthesis of *anti-***5**.

Hence, non-acidic epoxide opening conditions were investigated to circumvent the rearrangement (Scheme 4). Both the use of Bu_4_NH_2_F_3_ [18] and of the TBAF/KHF_2_ reagent combination [22] were successful (56% and 64%, respectively). While subsequent fluorination using PyFluor only led to the formation of the 2-pyridinesulfonate intermediate (*±*)-**12**, the use of DAST at 60 °C proved successful. The difluoride *meso*-**11** was not isolated due to its low boiling point, but was immediately subjected to acid hydrolysis to give *anti*-**5**. Unfortunately, the yield for this two-step process was only moderate (30%).

### Synthesis of (*±*)-*syn*-**5**

**Scheme 5:**
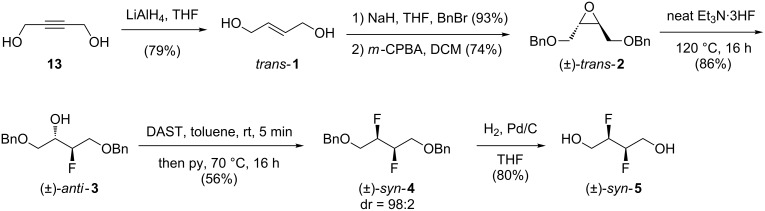
Synthesis of (*±*)-*syn*-**5**.

The synthesis of (*±*)-*syn*-**5** (Scheme 5) was achieved starting from the *trans-*configured but-2-ene-1,4-diol (**1**), which is not commercially available in geometrically pure form.

Hence, according to literature procedures, reduction of 1,4-butynediol (**13**) by LiAlH_4_ to give *trans*-**1** [27] was followed by benzylation [28] and epoxidation with *m-*CPBA to give (*±*)-*trans*-**2** [28]. When the reaction was performed on a small scale, excess *m-*CPBA and the byproduct 3-chlorobenzoic acid were removed by extraction with a saturated Na_2_S_2_O_3_ solution. However, on scale-up this proved inconvenient due to the large volumes of solvent required, and so these impurities were precipitated out the reaction mixture by cooling to 0 °C and collected by filtration through Celite. After work-up, the obtained epoxide was of high purity and no additional chromatographic purification was required, which was convenient on scale. The reaction of (*±*)-*trans-***2** with neat Et_3_N·3HF at 120 °C for 16 h led, after aqueous work-up, to (*±*)-*anti*-**3** in high diastereomeric purity (see Supporting Information File 1). The ^19^F shift of −195.3 ppm is different compared to that of (*±*)-*syn*-**3** (−204.4 ppm) [17]. Upon scale-up of the reaction to 10 g of (*±*)-*trans-***2**, a similarly high yield of 90% (crude) was obtained, which again could be used directly in the next step without purification. Conversion of fluorohydrin (*±*)-*anti*-**3** to difluoride (*±*)-*syn-***4** under the same conditions as shown in Scheme 2 resulted in 56% yield after column chromatography. ^19^F NMR analysis of the crude product showed a dr of 98:2 in favour of (*±*)-*syn*-**4**. However, given a diastereomerically pure starting material was used, this indicates that S_N_1 or neighbouring group participation pathways may have occurred, although only to a very small extent. Separation of the diastereomers proved not possible. Finally, deprotection of (*±*)-*syn*-**4** by palladium catalysed hydrogenolysis led to (*±*)-*syn*-**5**. Recrystallization to remove the minor diastereomer was not successful.

## Conclusion

A gram-scale synthesis of both *syn-* and *anti*-2,3-difluorobutan-1,4-diol diastereomers is described. The key steps involve epoxide opening and subsequent deoxyfluorination. For the first step, Et_3_N·3HF was found to be the best reagent, giving an excellent yield with no formation of diastereomeric byproducts. Unfortunately it was found that the subsequent DAST-mediated deoxyfluorination gives rise to a small amount of the undesired diastereomer. The primary alcohol groups require protection, for which the benzyl group has been employed. While this group is effective for this purpose, there is a significant mass increase upon its introduction (roughly three fold increase). An investigation to use the much smaller acetonide protecting group, which can be used for the *cis-*1,4-butenediol starting material, was carried out. It was found that the use of Et_3_N·3HF for the epoxide opening step also lead to acetal rearrangement, leading to a more stable 1,3-dioxolane ring. While the use of Bu_4_NH_2_F_3_/KHF_2_ and TBAF/KHF_2_ achieves epoxide opening without acetonide rearrangement, the subsequent deoxyfluorination/deprotection sequence is low yielding (30%). Overall, the protocols provided will be of use for the large-scale synthesis of both *syn-* and *anti*-2,3-difluorobutan-1,4-diol building blocks.

## Supporting Information

File 1 Experimental part and NMR spectra.
