# Supplementary material for: The synthesis of the 2,3-difluorobutan-1,4-diol diastereomers
Source: Beilstein J Org Chem. 2017 Dec 27;13:2883–7. doi: 10.3762/bjoc.13.280 (PMC5753060; doi:10.3762/bjoc.13.280)

Supporting Information

for

The synthesis of the 2,3-difluorobutan-1,4-diol diastereomers

Robert Szpera, Nadia Kovalenko, Kalaiselvi Natarajan, Nina Paillard and Bruno Linclau*

Address: Chemistry, University of Southampton, Highfield, Southampton SO17 1BJ, United Kingdom

Email: Bruno Linclau - bruno.linclau@soton.ac.uk

* Corresponding author

Experimental part and NMR spectra

Table of Contents

1 Experimental Section **S5**

1.1 **The synthesis of *anti*-5** **S5**

1.1.1 (±)-*syn*-1,4-Bis(benzyloxy)-3-fluorobutan-2-ol ((±)-*syn*-3) S5

1.1.2 *anti*-1,4-Bis(benzyloxy)-2,3-difluorobutane (*anti*-4) S6

1.1.3 2,2-Dimethyl-1,3-dioxep-5-ene (6) [2]: S6

1.1.4 4,4-Dimethyl-3,5,8-trioxabicyclo[5.1.0]octane (7) [3]: S7

1.1.5 *syn*-2-Fluoro-3,4-*O*-isopropylidene butane-1,3,4-triol ((±)-9): S7

1.1.6 4-(1,2-Difluoroethyl)-2,2-dimethyl-1,3-dioxolane ((±)-10): S8

1.1.7 (±)-6-Fluoro-2,2-dimethyl-1,3-dioxepan-5-ol ((±)-8): S9

1.1.8 Deoxofluorination of (±)-8 ..... S10

1.2 Synthesis of (±)-*syn*-5 ..... **S11**

1.2.1 *(E)*-But-2-ene-1,4-diol (*trans*-1) [7]: S11

1.2.2 *(E)*-1,4-Dibenzyloxybut-2-ene (*trans*-14) [7]: S12

1.2.3 (±)-*trans*-1,2-Bis((benzyloxy)methyl)oxirane ((±)-*trans*-2) [7]: S13

1.2.4 (±)-*Anti*-1,4-bis(benzyloxy)-3-fluorobutan-2-ol ((±)-*anti*-3): S13

1.2.5 4.2.5 (±)-*Syn*-1,4-bis(benzyloxy)-2,3-difluorobutane ((±)-*syn*-4): S14

1.2.6 4.2.6 (±)-*Syn*-2,3-difluorobutane-1,4-diol ((±)-*syn*-5): S15

1.3 References **S15**

2 Copies of Spectra **S16**

2.1 *syn*-1,4-Bis(benzyloxy)-3-fluorobutan-2-ol ((±)-*syn*-3) **S16**

2.1.1 ^{1}H NMR (400 MHz, $CDCl_3$) S16

2.1.2 ^{13}C NMR (100 MHz, $CDCl_3$) S17

2.1.3 ^{13}C DEPT-135 NMR ^{13}C NMR (100 MHz, $CDCl_3$) S18

2.1.4 ^{19}F NMR (376 MHz, $CDCl_3$) S19

2.1.5 Diastereoselectivity of the epoxide opening S20

2.2 2,2-Dimethyl-1,3-dioxep-5-ene (6) **S22**

2.2.1 ^{1}H NMR (400 MHz, $CDCl_3$) S22

2.2.2 ^{13}C NMR (100 MHz, $CDCl_3$) S23
2.3 4,4-Dimethyl-3,5,8-trioxabicyclo[5.1.0]octane (7) S24
2.3.1 ^{1}H NMR (400 MHz, $CDCl_3$) S24
2.3.2 ^{13}C NMR (100 MHz, $CDCl_3$) S25
2.4 *syn*-2-Fluoro-3,4-*O*-isopropylidene butane-1,3,4-triol ((±)-9) S26
2.4.1 ^{1}H NMR (400 MHz, $CDCl_3$) S26
2.4.2 $^{1}H\{OH\}$ NMR (500 MHz, $CDCl_3$)(D_2O exchange experiment) S27
2.4.3 $^{1}H\{^{19}F\}\{OH\}$ NMR (500 MHz, $CDCl_3$)(D_2O exchange experiment) S28
2.4.4 ^{19}FNMR of ±9 (471 MHz, $CDCl_3$) S29
2.4.5 ^{13}C NMR (100 MHz, $CDCl_3$) S30
2.5 4-(1,2-Difluoroethyl)-2,2-dimethyl-1,3-dioxolane ((±)-10) S31
2.5.1 ^{1}H NMR (500 MHz, $CDCl_3$) S31
2.5.2 $^{1}H\{^{19}F\}$ NMR (500 MHz, $CDCl_3$) S32
2.5.3 ^{19}F NMR (471 MHz, $CDCl_3$) S33
2.5.4 $^{19}F\{^{1}H\}$ (471 MHz, $CDCl_3$) S34
2.5.5 ^{13}C NMR (100 MHz, $CDCl_3$) S35
2.5.6 DEPT-135 (100 MHz, $CDCl_3$) S36
2.6 6-Fluoro-2,2-dimethyl-1,3-dioxepan-5-ol ((±)-8) S37
2.6.1 ^{1}H NMR (400 MHz, $CDCl_3$) S37
2.6.2 ^{13}C NMR (100 MHz, $CDCl_3$) S38
2.6.3 ^{19}F NMR (376 MHz, $CDCl_3$) S39
2.7 Sulfonate byproduct (±)-12 ..... S40
2.7.1 ^{1}H NMR (400 MHz, $CDCl_3$) S40
2.7.2 ^{13}C NMR (100 MHz, $CDCl_3$) S41
2.7.3 ^{19}F NMR (376 MHz, $CDCl_3$) S42
***2.8* *meso*-5,6-Difluoro-2,2-dimethyl-1,3-dioxepane (*meso*-11) S43**
2.8.1 ^{1}H NMR (400 MHz, $CDCl_3$) S43
2.8.2 ^{19}F NMR (376 MHz, $CDCl_3$) S44
2.8.3 ^{13}C NMR (100 MHz, $CDCl_3$) S45

2.9 *anti*-2,3-Difluorobutane-1,4-diol (*anti*-5)....S46
2.9.1 ^{1}H NMR (400 MHz, $CDCl_3$)....S46
2.9.2 ^{19}F NMR (376 MHz, $CDCl_3$)....S47
2.10 *(E)*-But-2-ene-1,4-diol (*trans*-1)....S48
2.10.1 ^{1}H NMR (400 MHz, $CDCl_3$)....S48
2.10.2 ^{13}C NMR (100 MHz, $CDCl_3$)....S49
2.11 *(E)*-1,4-Dibenzyloxybut-2-ene (*trans*-14)....S50
2.11.1 ^{1}H NMR (400 MHz, $CDCl_3$)....S50
2.11.2 ^{13}C NMR (100 MHz, $CDCl_3$)....S51
2.12 *trans*-1,2-Bis((benzyloxy)methyl)oxirane ((±)-*trans*-2)....S52
2.12.1 ^{1}H NMR (400 MHz, $CDCl_3$)....S52
2.12.2 ^{13}C NMR (100 MHz, $CDCl_3$)....S53
2.13 *anti*-1,4-Bis(benzyloxy)-3-fluorobutan-2-ol ((±)-*anti*-3)....S54
2.13.1 ^{1}H NMR (500 MHz, $CDCl_3$)....S54
2.13.2 ^{13}C NMR (100 MHz, $CDCl_3$)....S56
2.13.3 ^{19}F NMR (376 MHz, $CDCl_3$)....S57
2.13.4 Diastereoselectivity of the epoxide opening....S58
2.14 *syn*-1,4-Bis(benzyloxy)-2,3-difluorobutane ((±)-*syn*-4)....S60
2.14.1 ^{1}H NMR (500 MHz, $CDCl_3$)....S60
2.14.2 ^{13}C NMR (100 MHz, $CDCl_3$)....S61
2.14.3 ^{19}F NMR (376 MHz, $CDCl_3$)....S62
2.14.4 Diastereoselectivity of the DAST reaction....S64
2.15 *syn*-2,3-Difluorobutane-1,4-diol ((±)-*syn*-5)....S66
2.15.1 ^{1}H{^{19}F} NMR (500 MHz, $CDCl_3$)....S66
2.15.2 ^{13}C NMR (100 MHz, $CDCl_3$)....S67
2.15.3 ^{19}F NMR (376 MHz, $CDCl_3$)....S68

1 Experimental section

1.1 The synthesis of *anti*-5

1.1.1 (±)-*syn*-1,4-Bis(benzyloxy)-3-fluorobutan-2-ol ((±)-*syn*-3)

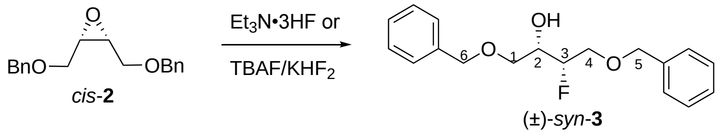

a) Opening with $Et_3N·3HF$: A mixture of epoxide *cis*-**2** (24.5 g, 86.2 mmol) and $Et_3N·3HF$ (37.5 mL, 230 mmol) were heated at 130 °C under argon for 16 h. The reaction mixture was diluted with ice cold water (300 mL), and then quenched with $NaHCO_3$ solution (230 mL). The mixture was extracted with Et_2O, then the combined extracts were dried over $MgSO_4$ and concentrated in vacuo to afford the crude product as a dark brown oil (26.1 g, 99%).

b) Opening with TBAF/KHF_2: TBAF·$3H_2O$ (5.23 g, 16.6 mmol) and KHF_2 (1.72 g, 22.1 mmol) were added sequentially to *cis*-**2** (3.14 g, 11.1 mmol) and the resulting mixture was heated at 115 °C for 22 h. The reaction mixture was allowed to cool to rt and was diluted with Et_2O and quenched with sat. $NaHCO_3$. The organic phase was separated and the aqueous phase was extracted with Et_2O. The combined organic phases were dried over $MgSO_4$ and filtered to give a brown oil. The crude was purified by column chromatography (20% ethyl acetate/petroleum ether) to give (±)-*syn*-**3** as a pale yellow oil (2.52 g, 75%). **^{1}H NMR** (400 MHz, $CDCl_3$) δ 7.39-7.28 (10H, m, ArH), 4.73 (1H, ddt, *J* = 47.1, 5.5, 3.4 Hz, H-3), 4.62-4.52 (4H, m, H-5 and H-6), 4.04 (1H, dm, *J* = 22.3 Hz, H-2), 3.81 (1H, ddd, *J* = 23.1, 11.4, 3.7 Hz, H-4 or H-4'), 3.75 (1H, ddd, *J* = 24.3, 11.4, 5.4 Hz, H-4 or H-4'), 3.63 (1H, ddd, *J* = 9.8, 5.3, 1.0 Hz, H-1 or H-1'), 3.59 (1H, ddd, *J* = 9.8, 6.4, 1.0 Hz, H-1 or H-1'), 2.62 (1H, dd, *J* = 4.7, 0.7 Hz) ppm; **^{13}C NMR** (101 MHz, $CDCl_3$), δ 137.7 (C_{Ar}), 137.5 (C_{Ar}), 128.4 (CH_{Ar}), 127.8 (CH_{Ar}), 127.7 (CH_{Ar}), 91.6 (d, *J* = 176.1 Hz, C-3), 73.7 (C-5 or C-6), 73.5 (C-5 or C-6), 70.2 (d, *J* = 5.1 Hz, C-1), 70.1 (d, *J* = 19.8 Hz, C-2), 69.6 (d, *J* = 22.7 Hz, C-4) ppm; **^{19}F NMR** (376 MHz, $CDCl_3$) δ -204.4 (1H, dq, *J* = 46.8, 23.1 Hz, F-3) ppm. Data matches literature [1].

1.1.2 *anti*-1,4-Bis(benzyloxy)-2,3-difluorobutane (*anti*-4)

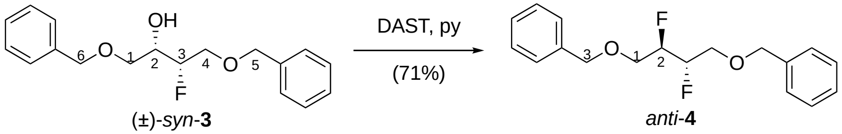

To a solution of fluorohydrin (±)-*syn*-**3** (10.6 g, 34.8 mmol) in dry toluene (50 mL) under argon, DAST (6.2 mL, 47 mmol) was added dropwise. After complete addition, the mixture was stirred at room temperature for 5 min, and then pyridine (7.3 mL, 90 mmol) was added. The reaction mixture was heated at 70 °C for 16 h behind a blast shield, and was then poured into ice cold $NaHCO_3$ solution (100 mL). The mixture was extracted with Et_2O (3 × 100 mL), and the combined extracts were washed with $NaHCO_3$ solution (300 mL), brine (300 mL), dried over $MgSO_4$, and concentrated in vacuo to afford a dark oil. Purification by flash chromatography (0–8% ethyl acetate in petrol) afforded the desired product as a yellow crystalline solid (7.57 g, 71%). **^{1}H NMR** (400MHz, $CDCl_3$): δ 7.39 – 7.29 (10H, m, Ar-H), 4.95 – 4.76 (2H, m, H-1), 4.61 (4H, s, H-3), 3.88 – 3.69 (4H, m, H-2) ppm. **^{13}C NMR** (101 MHz, $CDCl_3$): δ 137.6 (C_{Ar}), 128.4 (CH_{Ar}), 127.8 (CH_{Ar}), 127.7 (CH_{Ar}), 89.5 (2C, dd, J = 177.5, 28.6 Hz, C-1), 73.6 (2C, C-3), 68.2 (2C, m, C-2) ppm. **^{19}F NMR** (377 MHz, $CDCl_3$): δ -199.1 (2F, m, CHF). Data matches literature [1].

1.1.3 2,2-Dimethyl-1,3-dioxep-5-ene (6) [2]:

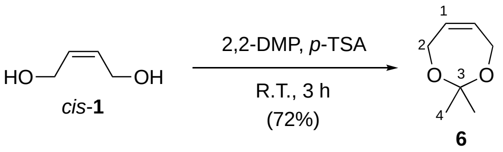

To a solution of *cis*-but-2-ene-1,4-diol (**1**, 9.3 mL, 113 mmol) in DCM, *p*-TSA·H_2O (1.08 g, 6.2 mmol) was added under argon at room temperature followed by 2,2-dimethoxypropane (27.9 mL, 227 mmol). The reaction mixture was allowed to stir for 3 h. After the completion of the reaction, the reaction mixture was quenched with sat. $NaHCO_3$ solution (50 mL) and extracted with DCM (2 × 50 mL). The combined organic layers were washed with brine (25 mL), dried over $MgSO_4$ and the solvent was very slowly removed in vacuo to yield **6** as colourless oil (10.5 g, 72%). The crude product was directly taken to the next step without purification. R_f = 0.51 (EtOAc/hexane 20%); **^{1}H NMR** (400 MHz,

$CDCl_3$) δ 5.66 (2H, t, *J* = 1.6 Hz, H-1), 4.25 (4H, 2, *J* = 1.6 Hz, H-2), 1.43 (6H, s, H-4) ppm; **^{13}C NMR** (101 MHz, $CDCl_3$): δ 129.5 (2C, C-1), 102.0 (1C, C-3), 61.4 (2C, C-2), 24.0 (2C, C-4) ppm. Data matches literature [3].

1.1.4 4,4-Dimethyl-3,5,8-trioxabicyclo[5.1.0]octane (7) [3]:

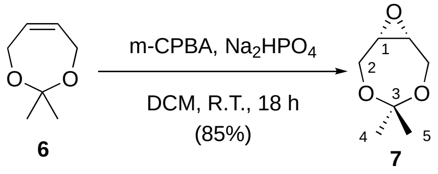

To a solution of **6** (10.0 g, 78 mmol) in DCM (400 mL) *m*-CPBA (27.0 g, 156 mmol) and finely powdered Na_2HPO_4 (12.7 g, 90 mmol) were added under argon. The reaction mixture was stirred at room temperature for 18 h, and was then chilled to 0 °C. The precipitate was removed by filtration through Celite, and the filtrate was washed sequentially with sat. $NaHCO_3$ (150 mL), sat. $Na_2S_2O_3$ (150 mL), and then brine. The organic phase was then dried over $MgSO_4$ and concentrated in vacuo*.* The crude product was purified by column chromatography (EtOAc/hexane 5% to 30%) to afford **7** as a pale yellow oil (9.6 g, 85%). R_f = 0.21 (EtOAc/hexane 20%); **^{1}H NMR** (400 MHz, $CDCl_3$): δ 4.06-3.96 (4H, m, H-2 and H-2^1), 3.21-3.18 (2H, m, H-1), 1.38 (3H, s, H-4 or H-5), 1.32 (3H, s, H-4 or H-5) ppm; **^{13}C NMR** (101 MHz, $CDCl_3$): δ 102.2 (1C, C-3), 60.0 (2C, C-2), 56.4 (2C, C-1), 24.7 (1C, C-4 or C-5), 23.4 (1C, C-4 or C-5) ppm; Data matches literature [4].

1.1.5 *syn*-2-Fluoro-3,4-*O*-isopropylidene butane-1,3,4-triol ((±)-9):

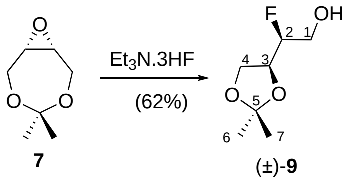

Epoxide **7** (1.0 g, 6.9 mmol) and neat $Et_3N·3HF$ (3.4 mL, 20.8 mmol) were added to a flask under argon and heated at 60 °C for 24 h. The reaction mixture was slowly added to ice cold sat. $NaHCO_3$. The aqueous phase was extracted with Et_2O, and the combined organic phases were washed with saturated aqueous $NaHCO_3$ solution, brine, dried over anhydrous $MgSO_4$, and concentrated in vacuo at room temperature to

afford a yellow oil which was purified by column chromatography (2–5% acetone in DCM) to afford (±)-**9** as a colourless oil (0.7 g, 62%). **IR** (neat): ν_{max} (cm^{-1}) 3414 (br), 2988 (w), 2360 (w), 1373 (m), 1215 (m), 1056. **^{1}H NMR** (400 MHz, $CDCl_3$): δ 4.52 (1H, ddt, *J* = 48.1, 5.6, 3.8 Hz, H-2), 4.32 (1H, dtd, *J* = 20.2, 6.8, 4.3 Hz, H-3), 4.10 (1H, ddd, *J* = 8.4, 6.9, 1.4 Hz, H-4 or 4'), 3.93 (1H, dd, *J* = 8.4, 6.8 Hz, H-4 or 4'), 3.86 (2H, m, H-1 and H-1'. This multiplet simplifies to: 3.87 (1H, dd, *J* = 12.7, 5.5 Hz), and 3.83 (1H, dd, *J* = 12.6 and 3.7 Hz) upon ^{19}F and OH decoupling, spectra taken on 500 MHz), 2.24 (1H, dd, *J* = 7.0, 5.6 Hz, OH), 1.45 (3H, s, H-6 or H-7), 1.39 (3H, s, H-6 or H-7) ppm; **^{13}C NMR** (101 MHz, $CDCl_3$): δ 110.1 (1C, C-5), 92.2 (1C, d, *J* = 177.5 Hz C-2), 74.9 (1C, d, *J* = 19.8 Hz, C-3), 64.9 (1C, d, *J* = 5.9 Hz, C-4), 62.5 (1C, d, *J* = 22.7, C-1), 26.1 (1C, C-6 or C-7), 25.4 (1C, C-6 or C-7) ppm; **^{19}F NMR** (471 MHz, $CDCl_3$): δ -202.3 (1F, m, F-2) ppm. These data qualitatively match literature data (all H signals were described as multiplet, and no *J*-values described in ^{13}C spectrum) [5].

1.1.6 4-(1,2-Difluoroethyl)-2,2-dimethyl-1,3-dioxolane ((±)-10):

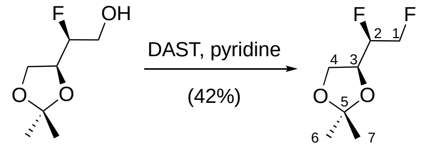

(±)-**9** (±)-**10**

To a solution of (±)-**9** (0.210 g, 1.28 mmol) in dry toluene (4 mL) under argon, DAST (0.26 mL, 1.97 mmol) was added dropwise. The solution was stirred for 5 minutes, and then dry pyridine (0.31 mL, 3.84 mmol) was added. The reaction mixture was stirred for 17 h at room temperature, and then further DAST (0.13 mL, 0.98 mmol) was added. Stirring was continued for 3 more hours, and then the reaction mixture was quenched by pouring into sat. $NaHCO_3$ solution (7 mL). The mixture was extracted with Et_2O (3 × 7 mL), and the combined organic layers were washed with sat. $NaHCO_3$ (20 mL), brine (20 mL), dried over $MgSO_4$, and concentrated in vacuo at rt to afford a crude yellow oil (0.210 g). The crude was purified by column chromatography (10–30% Et_2O in pentane) to afford (±)-**10** as a clear oil (0.090 g, 42%). **IR** (neat): ν_{max} (cm^{-1}) 2990 (w) (2939 (w) 2895 (w) 1457 (w) 1373 (m) 1218 (m) 1072 (s). **^{1}H NMR** (500 MHz, $CDCl_3$): δ 4.76 – 4.53 (3H, m, H-1, H-1', H-2), 4.30 (1H, dtd, *J* = 20.5, 6.7, 4.0 Hz, H-3, simplifies to td, *J* = 6.6, 3.9 Hz upon fluorine decoupling), 4.11 (1H, dddd, *J* = 8.5, 6.9, 1.4, 0.8 Hz, H-4 or H-4', simplifies to dd, *J* = 8.6, 6.8 Hz upon fluorine decoupling), 3.96 (1H, dd, *J* = 8.5, 6.6 Hz, H-4 or H-4[1]), 1.44 (3H, q appears as br d, *J* = 0.6 Hz, H-6 or H-7), 1.38 (3H, q, *J* = 0.6 Hz, H-6 or H-7) ppm. **^{13}C NMR** (101 MHz, $CDCl_3$): δ 110.3 (1C, C-5), 90.1 (1C, dd, *J* = 181.2, 19.1 Hz, C-2),

82.0 (1C, dd, J = 172.4, 24.2 Hz, C-1), 73.8 (1C, dd, J = 20.5, 7.3 Hz, C-3), 64.7 (1C, d, J = 5.9 Hz, C-4), 26.0 (1C, C-6 or 7), 25.3 (1C, C-6 or 7) ppm. $\{^1H\}^{19}$**F NMR** (471 MHz, $CDCl_3$): δ -203.0 (1F, d, J = 14.7 Hz, F-2), -232.3 (1F, d, J = 15.0 Hz, F-1) ppm. **MS** (ESI^+, MeCN): 167.1 $(M+H)^+$.

1.1.7 (±)-6-Fluoro-2,2-dimethyl-1,3-dioxepan-5-ol ((±)-8):

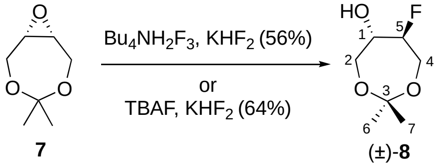

Reaction with $Bu_4NH_2F_3/KHF_2$: To the mixture of **7** (0.18 g, 1.25 mmol) and $Bu_4NH_2F_3$ (0.56 g, 1.873 mmol), KHF_2 (0.20 g, 2.50 mmol) was added at rt under argon. The reaction mixture was allowed to stir for 20 hours at 115 °C. After completion of the reaction, the reaction mixture was quenched with sat. $NaHCO_3$ (10 mL) and extracted with DCM (20 mL). Then the organic layer was washed with brine, dried over anhydrous $MgSO_4$ and concentrated in vacuo. The crude obtained was purified by column chromatography (acetone/DCM 1% to 4%) to afford fluorohydrin (±)-**8** as colourless oil (0.12 g, 56%).

Reaction with TBAF/KHF_2: TBAF·$3H_2O$ (15.6 g, 49.6 mmol) and KHF_2 (4.6 g, 58.3 mmol) were added sequentially to **7** (4.2 g, 29.2 mmol) under argon and the resulting mixture was heated at 115 °C for 20 h. The dark brown mixture was then cooled to room temperature, diluted with Et_2O (20 mL) and water (20 mL) and slowly quenched with sat. $NaHCO_3$ (100 mL). The aqueous layer was extracted with Et_2O (100 mL) and the combined organic layers were washed with brine, dried over $MgSO_4$ and filtered through silica plug to remove the brown colour. The solvent was removed in vacuo and the crude was purified by column chromatography (5% acetone/DCM) to give fluorohydrin (±)-**8** as pale yellow oil (3.1 g, 64%). R_f = 0.55 (acetone/DCM 10%); ^{1}**H NMR** (400 MHz, $CDCl_3$) δ 4.42 (1H, dm, J = 44.0 Hz, H-5), 3.99 (1H, ddd, J = 12.7 , 2.2, 1.5 Hz, H-2), 3.92 - 3.78 (3H, m, H-4, H-4' and H-1), 3.59 (1H, ddd, J = 12.7, 4.5, 1.2 Hz, H-2'), 2.62 (1H, d, J = 8.2 Hz, OH), 1.38 (3H, s, H-6 or H-7), 1.36 (3H, s, H-6 or H-7) ppm; ^{13}**C NMR** (101 MHz, $CDCl_3$) δ 101.8 (C-3), 91.7 (d, J = 176.1 Hz, C-5), 70.0 (d, J = 28.6 Hz, C-1), 60.3 (C-2), 59.3 (d, J = 20.5 Hz, C-4), 24.9 (C-6 or C-7), 23.9 (C-6 or C-7) ppm; ^{19}**F NMR** (376 MHz, $CDCl_3$) δ -194.5 (m, 1F) ppm. **HRMS** (ESI^+) $(M+Na)^+$ calcd: 187.0740, found 187.0741. Data matches literature [6].

1.1.8 Deoxofluorination of (±)-8

- with PyFluor, which gives 6-fluoro-5-(2-pyridylsulfonyl)-2,2-dimethyl-1,3-dioxepan-5-ol ((±)-**12**):

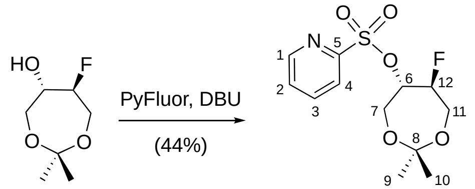

(±)-**8** (±)-**12**

To a solution of fluorohydrin (±)-**8** (0.20 g, 1.22 mmol) in toluene (0.02 mL), PyFluor (0.22 g, 1.34 mmol) and DBU (0.19 g, 2.44 mmol) were added and stirred at room temperature for 72 h. The reaction mixture was cooled to 0 °C and quenched with sat. $NaHCO_3$ followed by extraction with Et_2O. The organic layer was washed with brine and concentrated in vacuo. Column chromatography was performed (pentane/Et_2O 5–40%) to give the sulfonate (±)-**12** (0.09 g, 44%). **IR** (neat): ν_{max} (cm^{-1}) 2924 (m), 1580 (w), 1454 (w), 1429 (w), 1370 (s), 778 (s); **^{1}H NMR** (400 MHz, $CDCl_3$) δ 8.76 (1H, ddd, *J* = 4.7, 1.6, 0.9 Hz, H-12), 8.06 (1H, dt, *J* = 7.8, 1.0 Hz, H-4), 7.97 (1H, td, *J* = 7.7, 1.7 Hz, H-3), 7.59 (1H, ddd, *J* = 7.6, 4.7, 1.2 Hz, H-2), 5.00 (1H, dtdd, *J* = 13.2, 4.9, 2.5, 0.6 Hz, H-7 or H-7'), 4.62 (1H, dtdd, *J* = 44.0, 6.0, 2.3, 0.7 Hz, H-12), 4.00 (1H, dt, *J* = 13.3, 2.7 Hz, H-7 or H-7'), 3.93-3.74 (3H, m, H-6, H-11 and H-11'), 1.33 (6H, 2 x s, H-9 and H-10) ppm; **^{13}C NMR** (101 MHz, $CDCl_3$) δ 154.9 (C-5), 150.3 (C-1), 138.2 (C-3), 127.8 (C-2), 123.1 (C-4), 102.0 (C-8), 89.8 (d, *J* = 178.3 Hz, C-12), 80.9 (d, *J* = 29.4 Hz, C-7), 59.0 (m, C-6 and C-11), 24.2 (C-9 or C-10), 24.0 (C-9 or C-10); **^{19}F NMR** (376 MHz, $CDCl_3$) δ 193.7 (1F, ddd *J* = 44.0, 24.3, 8.7 Hz, F-12) ppm. **HRMS** (ESI^+, MeOH) $(M+Na)^+$ calcd: 328.0625, found 328.0629.

- with DAST, including deprotection, to give *anti*-2,3-difluorobutane-1,4-diol (*anti*-**5**):

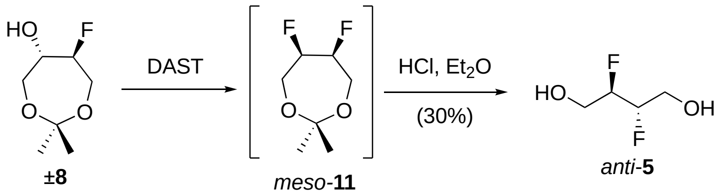

To solution of fluorohydrin (±)-**8** (1.19 g, 7.25 mmol) in toluene (3 mL), DAST (1.25 mL, 9.43 mmol) was added dropwise and the mixture was stirred for 5 min at rt. Pyridine (1.47 mL, 18.13 mmol) was added and the mixture was heated at 60 °C for 23 h behind a blast shield. The reaction mixture was cooled to rt and 2 M HCl in Et_2O (16.32 mL, 32.63 mmol) was added carefully and stirred at rt for 1 h. Et_2O was removed in vacuo and column chromatography was performed directly (30-50% acetone/petroleum ether) to give diol *anti*-**5** as an off-white solid (0.276 g, 30%). **^{1}H NMR** (400 MHz, acetone-d_6) δ 4.72 (2H, m, H-2), 4.19 (2H, t, *J* = 5.8 Hz, OH), 3.81 (4H, m, H-1) ppm; **^{19}F NMR** (376 MHz, acetone-d_6) δ -200.4 (m, F-1) ppm. **HRMS** (ESI+): $(M+Na)^+$ Calcd. 149.0382, Found 149.0385. Data matches literature [1].

An analytical sample of *meso*-**11** was isolated: **^{1}H NMR** (400 MHz, $CDCl_3$) δ 4.72–4.56 (2H, m, H-1), 4.05–3.97 (2H, m, H-2 or H-2'), 3.74–3.66 (2H, m, H-2 or H-2'), 1.39 (3H, s, H-4 or H-5), 1.33 (3H, s, H-4 or H-5) ppm; **^{13}C NMR** (101 MHz, $CDCl_3$) δ 101.9 (C-3), 88.3 (app dd, *J* = 183.4, 17.6 Hz, C-1), 58.1 (m, C-2), 24.7 (C-4 or C-5), 24.0 (C-4 or C-5) ppm; **^{19}F NMR** (376 MHz, $CDCl_3$) δ -208.5 (m, F-1) ppm.

1.2 Synthesis of (±)-*syn*-5

1.2.1 *(E)*-But-2-ene-1,4-diol (*trans*-1) [7]:

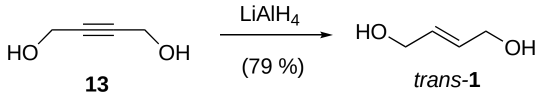

To a suspension of $LiAlH_4$ (5.290 g, 139.39 mmol) in THF (350 mL) at 0 °C under argon, was added a solution of **13** (10.000 g, 116.16 mmol) in THF (100 mL). The resulting mixture was stirred at room temperature for 36 h. The reaction mixture was then cooled to 0 °C, and the excess $LiAlH_4$ was quenched via sequential addition of H_2O (6 mL), 3 M aqueous NaOH (6 mL) and finally more H_2O (16 mL). $MgSO_4$ was added, and stirring was continued at room temperature for a further 30 min. The reaction mixture was filtered through celite, and the solid was washed with ethyl acetate (400 mL). The filtrate was concentrated in vacuo to afford a crude colourless oil (9.484 g). Purification via column chromatography (50–100% EtOAc in hexane) afforded *trans*-**1** as a colourless oil (8.128 g, 79%). **IR** (neat): ν_{max} 3281 (br), 2930 (m), 2854 (m), 1656 (w), 1417 (m), 1082 (s), 972 (s) cm^{-1}. **^{1}H NMR** (400MHz, $CDCl_3$): δ 5.89 (2H, t, *J* = 2.5 Hz, CH), 4.17 (4H, s, CH_2), 1.93 (2H, br s, OH) ppm. **^{13}C NMR** (101 MHz, $CDCl_3$): δ 130.5 (2C, CH), 62.8 (2C, CH_2) ppm. Data matches literature [7].

1.2.2 *(E)*-1,4-Dibenzyloxybut-2-ene (*trans*-14) [7]:

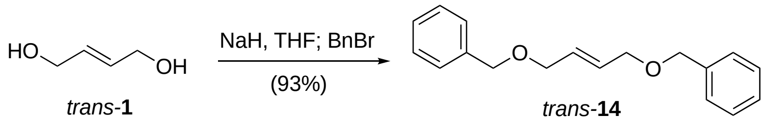

NaH (60% in dispersion oil; 8.856 g, 221.40 mmol) was added to a flask under argon and the mineral oil was removed by washing with hexane (3 × 130 mL). A solution of *trans*-**1** (8.128 g, 92.25 mmol) in THF (130 mL) was added to the NaH at 0 °C under argon, and the resulting mixture was stirred at reflux for 3.5 h. The reaction mixture was allowed to cool, benzyl bromide (26.33 mL, 221.40 mmol) was added, and stirring was continued at reflux for a further 3.5 h. The mixture was then cooled to room temperature and quenched with water (440 mL). The phases were separated and the aqueous was extracted with Et_2O (2 × 440 mL). The combined organic phases were washed sequentially with saturated aqueous NH_4Cl (440 mL), water (440 mL), and brine (440 mL), then dried over $MgSO_4$, and concentrated to afford a straw coloured crude oil (31.158 g). The crude was purified by column chromatography (0–10% EtOAc in petroleum ether) to afford *trans*-**14** as a pale yellow oil (22.976 g, 93%). **IR** (neat): ν_{max} 3029 (w), 2850 (m), 1496 (m), 1102 (s), 696 (s) cm^{-1}. **^{1}H NMR** (400MHz, $CDCl_3$): δ 7.39 – 7.31 (10H, m, Ar-H), 5.92 (2H, m, CHCH$_2$), 4.56 (4H, s, CH$_2$OBn), 4.09 (4H, m, CH$_2$CH) ppm. **^{13}C NMR** (101 MHz, $CDCl_3$): δ 138.2 (C_{Ar}), 129.5 (2C, CHCH$_2$), 128.3 (CH_{Ar}), 127.7 (CH_{Ar}), 127.6 (CH_{Ar}), 72.2 (2C, CH$_2$OBn), 70.1 (2C, CH$_2$CH) ppm. Data matches literature [7].

1.2.3 (±)-*trans*-1,2-Bis((benzyloxy)methyl)oxirane ((±)-*trans*-2) [7]:

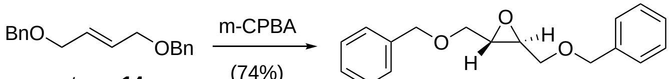

trans-**14** (±)-*trans*-**2**

m-CPBA (≤77, 9.501 g, 42.4 mmol) was added portionwise to a solution of *trans*-**14** (9.390 g, 35.0 mmol) in DCM (100 mL) at 0 °C. The resulting mixture was allowed to warm to room temperature and stirred for 16 hours. The mixture was cooled to 0 °C and the precipitate removed by filtration though celite, and the solid was washed with ice cold DCM (100 mL). The filtrate was washed with $NaHCO_3$ solution (200 mL), $Na_2S_2O_4$ solution (200 mL), and finally brine (200 mL). The organic phase was then dried over $MgSO_4$ and concentrated in vacuo to afford the epoxide (±)-*trans*-**2** as a yellow oil (7.321 g, 74%), which can be used directly in the next step.

Purification by column chromatography (0–15% EtOAc in petroleum ether) is possible. An experiment starting from 22.976 g of *trans*-**14** thus afforded 16.267 g *of trans*-**2** as a pale yellow oil (67%). **IR** (neat): ν_{max} 3030 (w), 2857 (m), 1453 (m), 873 (m), 734 (s), 696 (s) cm^{-1}. **^{1}H NMR** (400 MHz, $CDCl_3$): δ 7.39 – 7.29 (10H, m , Ar-H) 4.62 (2H, d, *J* 11.9 Hz, C<u>H</u>HPh), 4.56 (2H, d, *J* = 11.9 Hz, CH<u>H</u>Ph), 3.77 (2H, dd, *J* = 11.6, 2.7 Hz, CHC<u>H</u>H), 3.52 (2H, dd, *J* = 11.6, 5.3 Hz, CHCH<u>H</u>), 3.13 (2H, m, C<u>H</u>CH_2) ppm. **^{13}C NMR** (101 MHz, $CDCl_3$): δ 137.8 (C_{Ar}), 128.4 (CH_{Ar}), 127.7 (CH_{Ar}), 73.3 (2C, <u>C</u>H_2Ph), 69.8 (2C, CH<u>C</u>H_2), 54.4 (2C, C<u>H</u>CH_2) ppm. Data matches literature [7].

1.2.4 (±)-*anti*-1,4-Bis(benzyloxy)-3-fluorobutan-2-ol ((±)-*anti*-3):

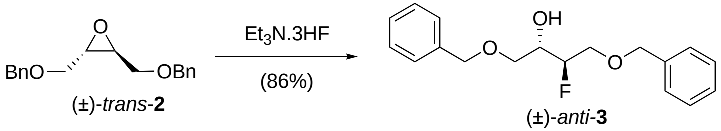

(±)-*trans*-**2** (±)-*anti*-**3**

$Et_3N·3HF$ (17.0 mL, 104 mmol) was added to a flask containing the crude epoxide (9.021 g, 31.70 mmol), and the mixture was heated at 120 °C for 16 h. The mixture was then diluted with water (100 mL) and poured into ice-cold $NaHCO_3$ solution. The aqueous was extracted with Et_2O (3 × 250 mL), and the combined extracts were washed with $NaHCO_3$ solution (500 mL), brine (500 mL), then dried over $MgSO_4$ and concentrated in vacuo to afford the *anti*-fluorohydrin (±)-*anti*-**3** as a dark oil (8.710 g, 90 %), which was used directly in the next step. Purification by column

chromatography (5–20% EtOAc in petroleum ether) is possible. An experiment starting from 10.401 g of (±)-*trans*-**2** thus afforded 9.602 g *of* (±)-*anti*-**3** as pale yellow oil (86%). **IR** (neat): v_{max} 3431 (br), 3064 (w), 3031 (w), 2916 (w), 2865 (w), 1496 (w), 1453 (m), 1103 (s), 1057 (s) cm^{-1}. **^{1}H NMR** (500 MHz, $CDCl_3$): δ H 7.39-7.29 (10H, m, Ar-H), 4.65 (1H, dddd, *J* = 47.4, 7.5, 5.0, 2.7 Hz, CHF), 4.60 (2H, m, CH_2Ph), 4.58 (2H, s, CH_2Ph), 4.04 (1H, m, CHOH), 3.85 (1H, ddd, *J* = 25.8, 11.5, 2.7 Hz, CHHCHF), 3.79 (1H, ddd, *J* = 27.8, 11.5, 5.1 Hz, CHHCHF), 3.69 (1H, ddd, *J* = 9.8, 3.6, 2.5 Hz, CHHCHOH), 3.65 (1H, ddd, *J* = 9.7, 5.6, 2.0 Hz, CHHCHOH), 2.59 (1H, d, *J* = 6.0 Hz, OH) ppm. **^{13}C NMR** (101 MHz, $CDCl_3$): δ 137.73 (C_{Ar}), 137.66 (C_{Ar}), 128.48 (CH_{Ar}), 128.43 (CH_{Ar}), 127.88 (CH_{Ar}), 127.77 (CH_{Ar}), 127.75 (CH_{Ar}), 127.70 (CH_{Ar}), 91.6 (d, *J* = 175.3 Hz, CHF), 73.6 (CH_2Ph), 73.5 (CH_2Ph), 70.1 (d, *J* = 5.1 Hz, CH_2CHOH), 69.3 (d, *J* = 20.5 Hz, CH_2CHF), 69.1 (d, *J* 24.9 Hz, CHOH). **^{19}F NMR** (471MHz, $CDCl_3$): δ -195.3 (1F, ddddt, *J* = 47.4, 27.9, 25.7, 8.2, 2.2 Hz). **HRMS** (ESI^+, MeCN): $(M+Na)^+$ Calcd. 327.1367, Found. 327.1374.

1.2.5 4.2.5 (±)-*syn*-1,4-Bis(benzyloxy)-2,3-difluorobutane ((±)-*syn*-4):

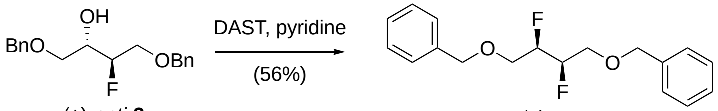

(±)-*anti*-**3** (±)-*syn*-**4**

To a solution of the crude fluorohydrin (±)-*anti*-**3** (15.811 g, 51.9 mmol) in dry toluene (75 mL) under argon, DAST (8.9 mL, 67.4 mmol) was added dropwise, and after complete addition the mixture was stirred at room temperature for 5 min. Pyridine (11.0 mL, 136 mmol) was then added and the reaction mixture was stirred at 70 °C for 16 h behind a blast shield. The mixture was then allowed to cool, poured into saturated aqueous $NaHCO_3$ (100 mL), and extracted with Et_2O (3 × 100 mL). The combined extracts were washed with $NaHCO_3$ solution (300 mL), brine (300 mL), dried over $MgSO_4$, and concentrated in vacuo to afford a yellow oil (12.552 g). The crude product was purified by column chromatography (0–6% EtOAc in petroleum ether) to afford the *syn*-difluoride (±)-*syn*-**4** as a dark oil (8.910 g, 56%). **IR** (neat): v_{max} 3031 (w), 2917 (w), 2866 (w), 2360 (w), 1496 (w), 1453 (m), 1117 (s), 1026 (s) cm^{-1}. **^{1}H NMR** (500 MHz, $CDCl_3$): δ 7.39 – 7.30 (10H, m, Ar-H), 4.84 (2H, m, CHF), 4.58 (2H, d, *J* = 12.0 Hz, CHHPh), 4.54 (2H, d, *J* = 12.0 Hz, CHHPh), 3.82 – 3.70 (4H, m, CH_2CH) **^{13}C NMR** (101 MHz, $CDCl_3$): δ 137.5 (CAr), 128.5 (CHAr), 127.9 (CHAr), 127.8 (CHAr), 90.5 (2C, dd, *J* = 179.0, 20.5 Hz, CHF), 73.7 (2C, CH_2Ph), 68.1 (2C, m, $CHCH_2$). **^{19}F NMR** (471 MHz, $CDCl_3$): δ -203.8 (2F, m). **HRMS** (ESI+, MeCN): $(M+Na)^+$ Calcd. 329.1324, Found 329.1330.

1.2.6 4.2.6 (±)-*Syn*-2,3-difluorobutane-1,4-diol ((±)-*syn*-5):

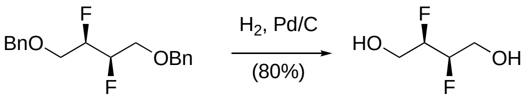

(±)-*syn*-**4** (±)-*syn*-**5**

To a solution of (±)-*syn*-**4** (3.01 g, 9.83 mmol) in THF (45 mL) under argon atmosphere was added Pd/C (5%; 4.18 g, 1.96 mmol). The flask was evacuated and then placed under hydrogen atmosphere and stirred at room temperature for 16 h. The suspension was filtered through celite, the celite was washed with methanol (50 mL), and the filtrate was concentrated in vacuo to afford a crude yellow crystalline solid (1.05 g). The crude was purified by column chromatography (15–50% acetone in petroleum ether) to afford (±)-*syn*-**5** as a white crystalline solid (0.993 g, 80%). **mp** 113–114 °C. **IR** (neat): ν_{max} 3279 (br), 2975 (w), 2944 (w), 1647 (m), 1462 (m), 1361 (m) cm^{-1}. **^{1}H NMR** (500 MHz, acetone-d_6): δ 4.70 (2H, m, C<u>H</u>F), 4.17 (2H, t, *J* = 5.9 Hz, OH), 3.88-3.78 (4H, m, C<u>H_2</u>) ppm. **^{13}C NMR** (101 MHz, acetone-d_6): δ 93.2 (2C, dd, *J* = 175.3, 19.1 Hz, <u>C</u>HF), 61.3 (2C, dd, *J* = 18.3, 13.9, <u>C</u>H_2). **^{19}F NMR** (471 MHz, acetone-d_6): -205.9 (2F, m). **HRMS** (ESI-): $(M-H)^+$ Calcd. 125.0417, Found 125.0420.

1.3 References

(1) Linclau, B.; Leung, L.; Nonnenmacher, J.; Tizzard, G. *Beilst. J. Org. Chem.* **2010**, *6*, 62.
(2) Elliott, W. J.; Fried, J. *J. Org. Chem.* **1976**, *41*, 2469.
(3) Al-Dulayymi, A.; Li, X. M.; Neuenschwander, M. *Helv. Chim. Acta* **2000**, *83*, 1633.
(4) Mischitz, M.; Kroutil, W.; Wandel, U.; Faber, K. *Tetrahedron-Asymmetry* **1995**, *6*, 1261.
(5) Bzowska, A.; Kulikowska, E.; Shugar, D.; Chen, B. Y.; Lindborg, B.; Johansson, N. G. *Biochem. Pharmacol.* **1991**, *41*, 1791.
(6) Kalow, J. A.; Doyle, A. G. *J. Am. Chem. Soc.* **2010**, *132*, 3268.
(7) Hachiya, I.; Matsumoto, T.; Inagaki, T.; Takahashi, A.; Shimizu, M. *Heterocycles* **2010**, *82*, 449.

2 Copies of spectra

2.1 *syn*-1,4-Bis(benzyloxy)-3-fluorobutan-2-ol ((±)-*syn*-3)

2.1.1 ^{1}H NMR (400 MHz, $CDCl_3$)

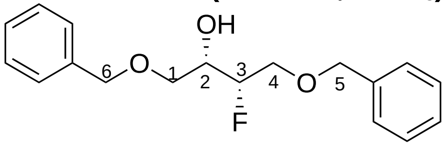

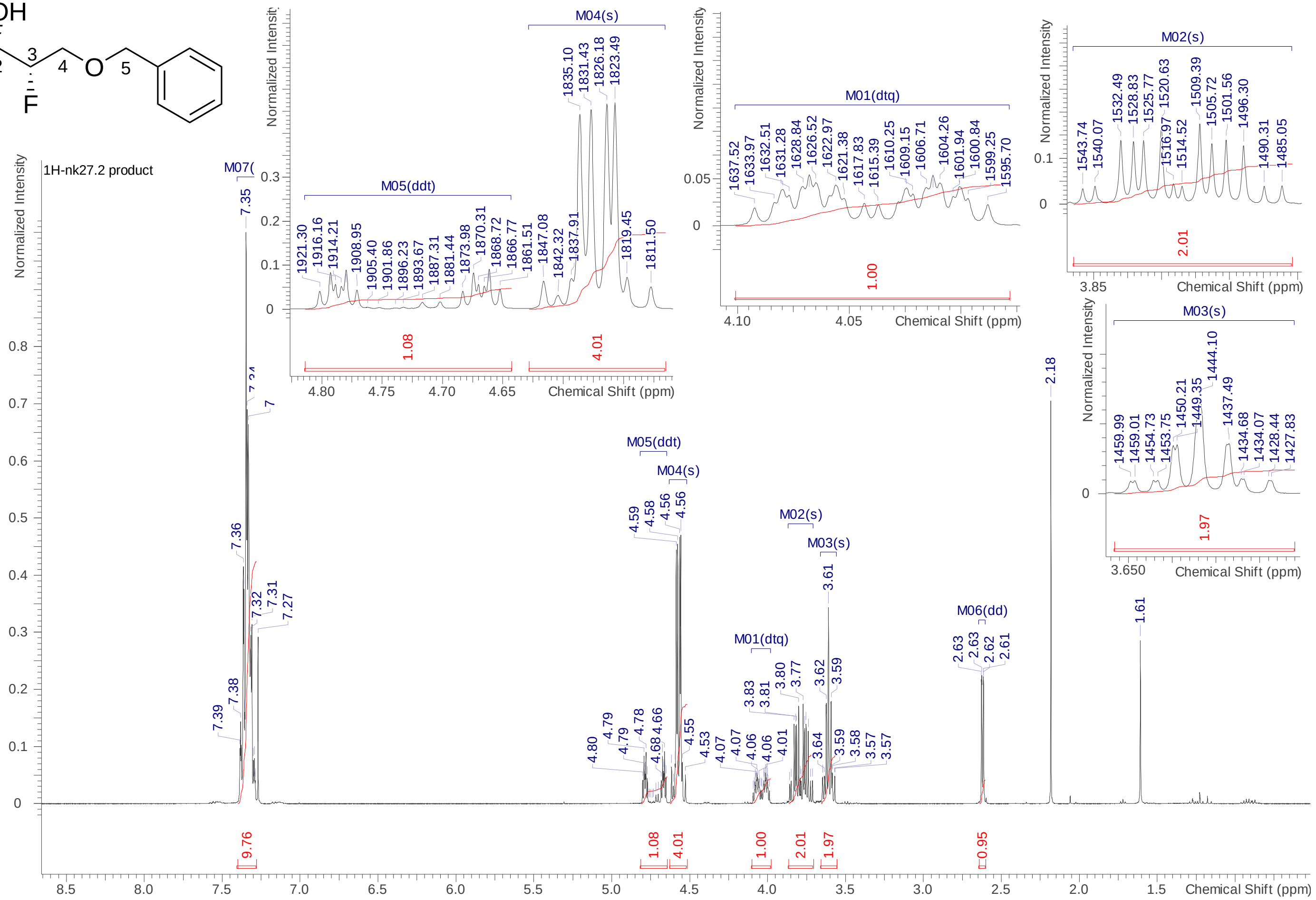

2.1.2 ^{13}C NMR (100 MHz, $CDCl_3$)

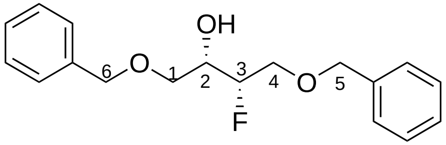

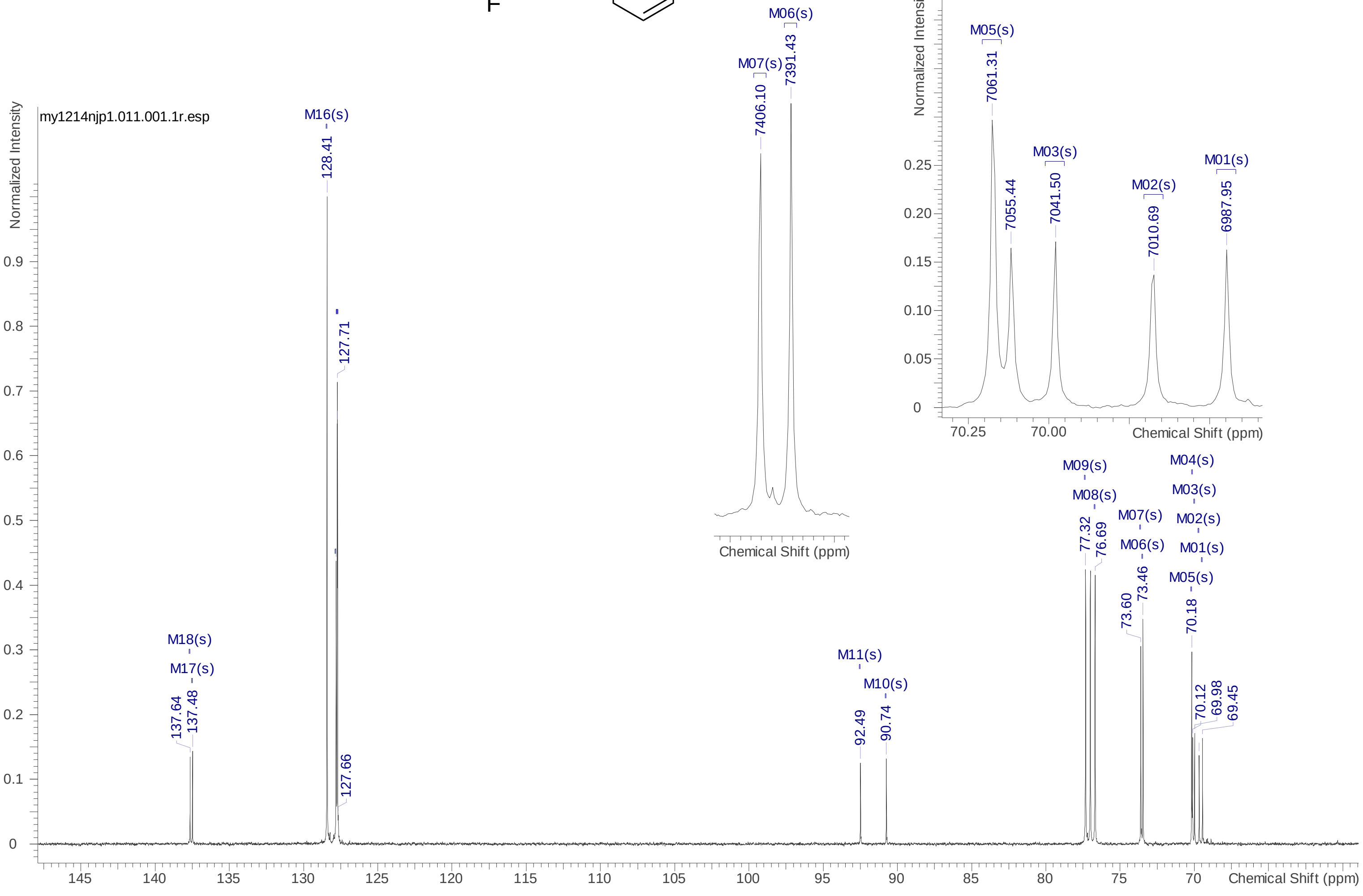

2.1.3 ^{13}C DEPT-135 NMR ^{13}C NMR (100 MHz, $CDCl_3$)

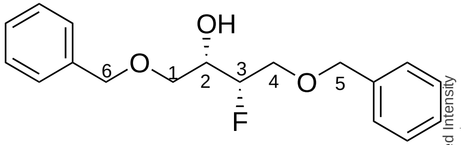

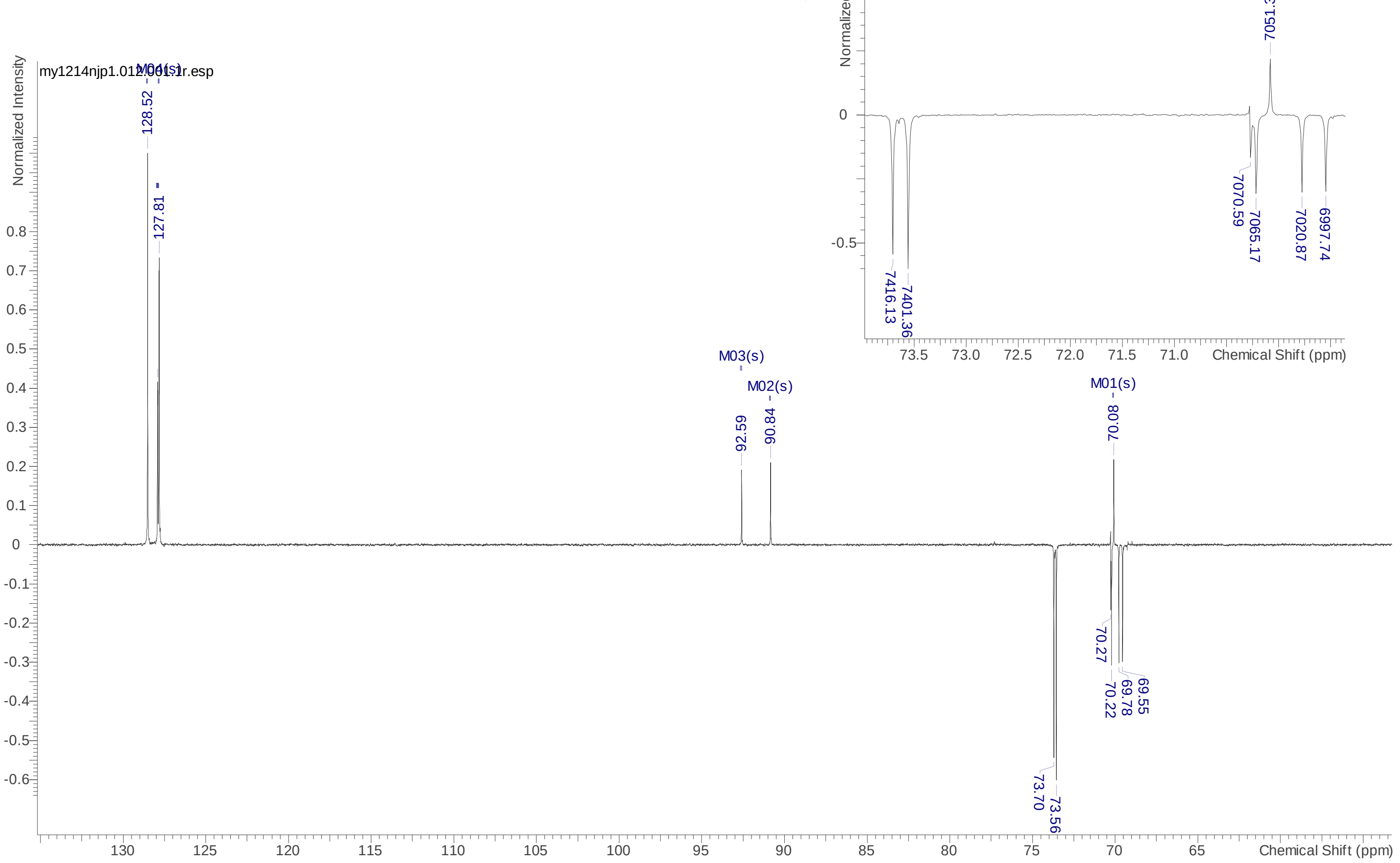

2.1.4 ^{19}F NMR (376 MHz, $CDCl_3$)

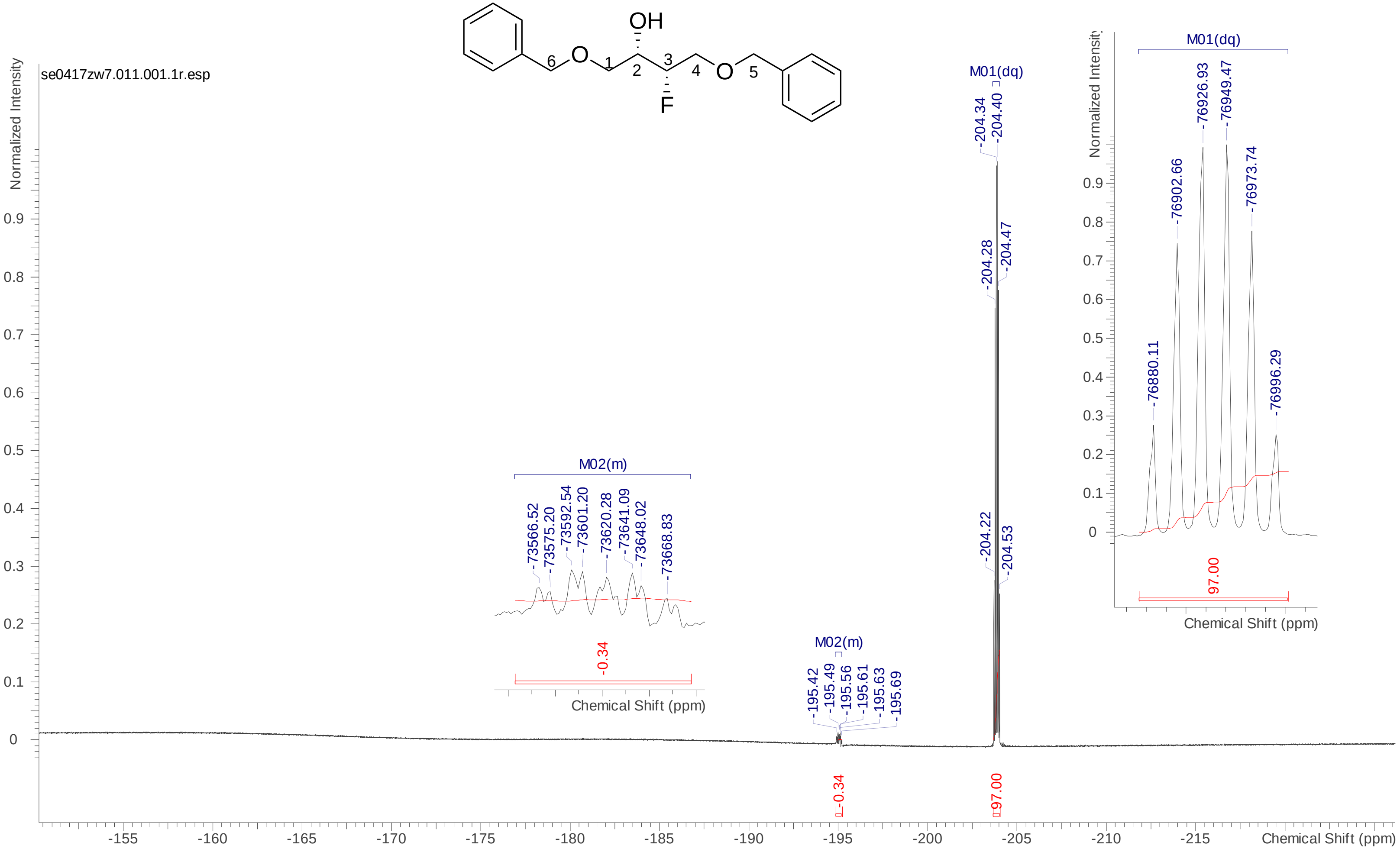

2.1.5 Diastereoselectivity of the epoxide opening

2.1.5.1 ^{1}H NMR of the *cis*-epoxide starting material, compared to that of the *trans*-isomer

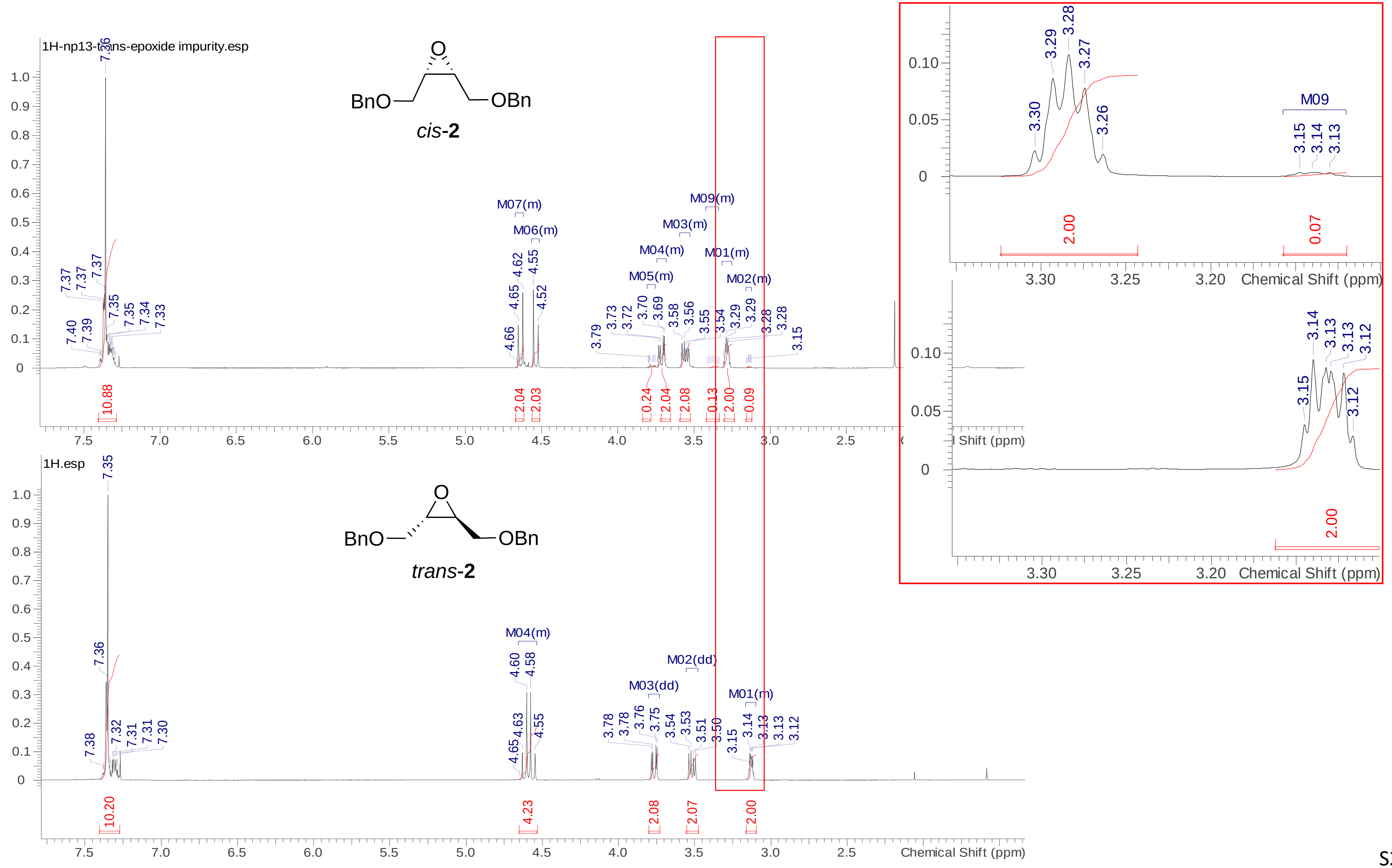

2.1.5.2 ^{19}F NMR of the fluorohydrin product *syn*-**3**, compared to that of the *anti*-isomer

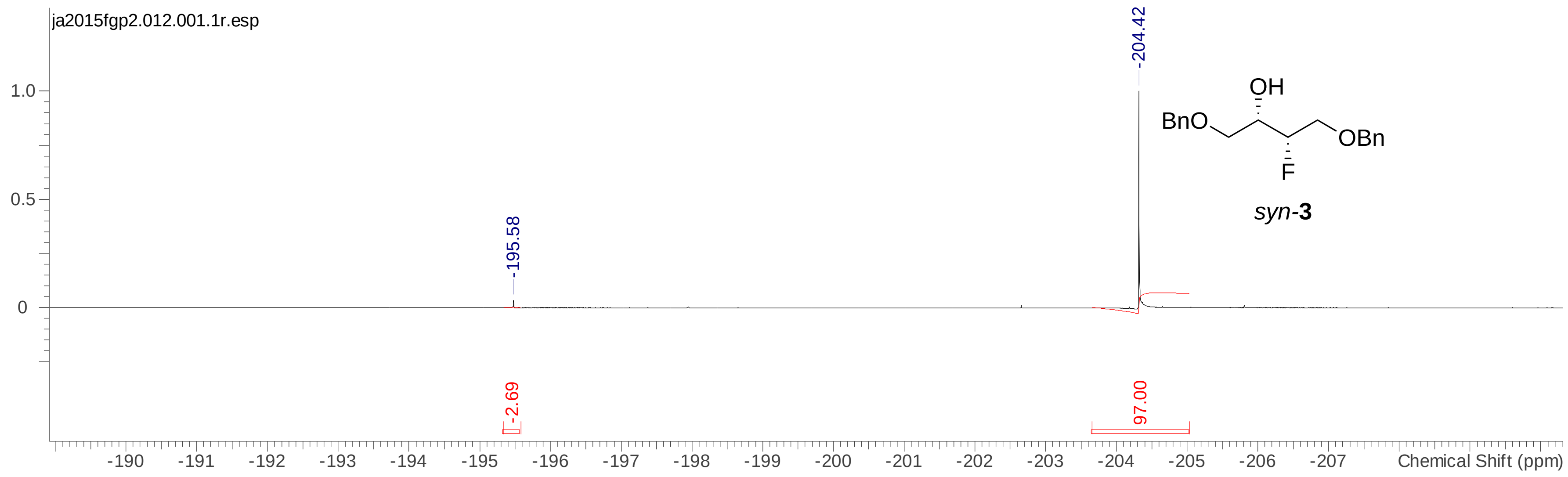

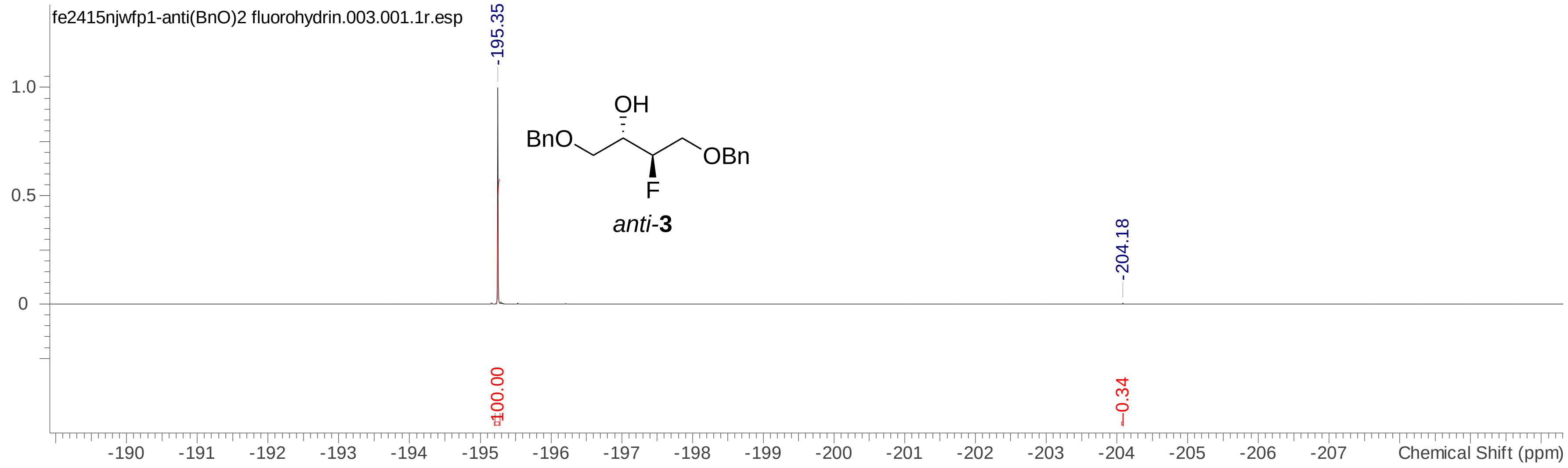

2.2 2,2-Dimethyl-1,3-dioxep-5-ene (6)

2.2.1 ^{1}H NMR (400 MHz, $CDCl_3$)

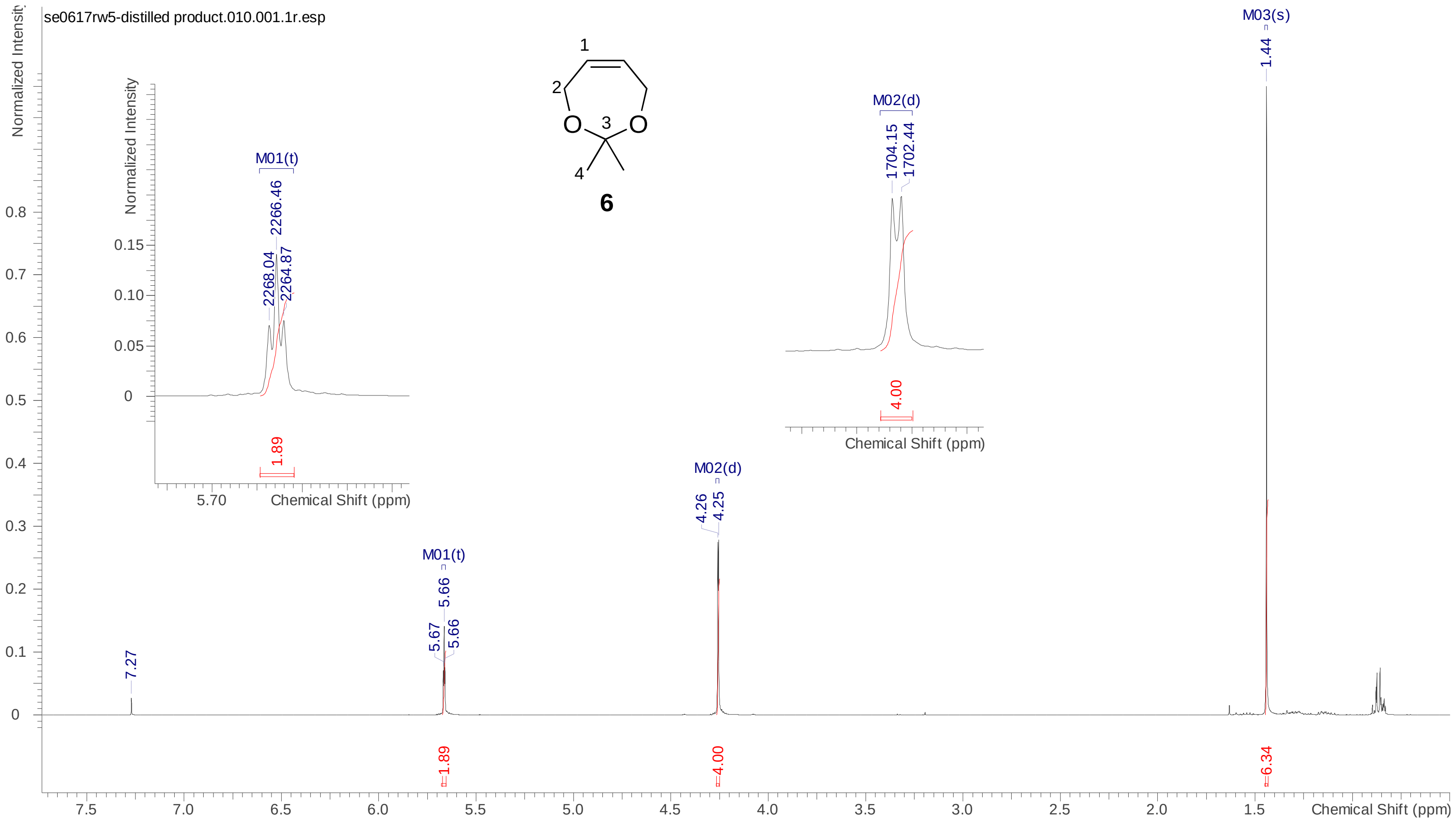

2.2.2 ^{13}C NMR (100 MHz, $CDCl_3$)

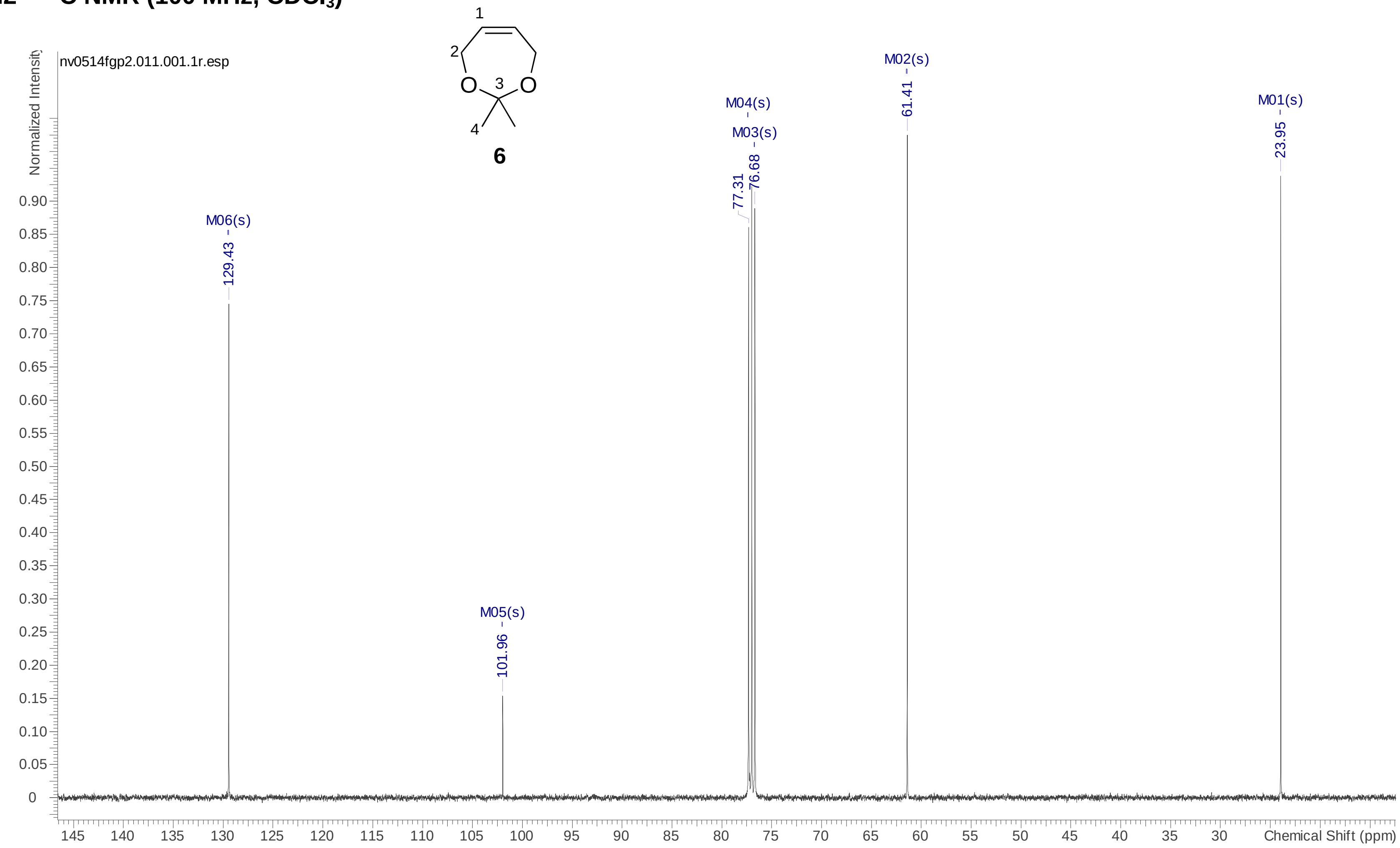

2.3 4,4-Dimethyl-3,5,8-trioxabicyclo[5.1.0]octane (7)

2.3.1 ^{1}H NMR (400 MHz, $CDCl_3$)

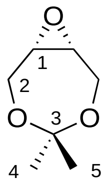

7

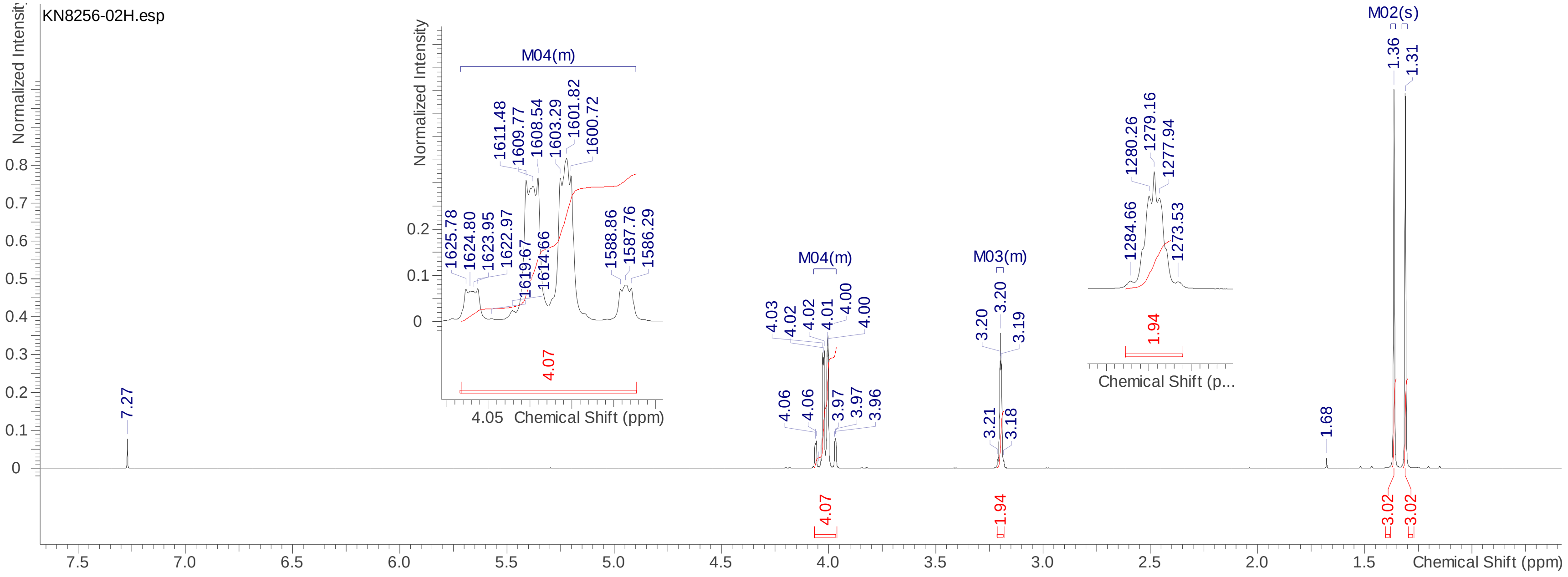

2.3.2 ^{13}C NMR (100 MHz, $CDCl_3$)

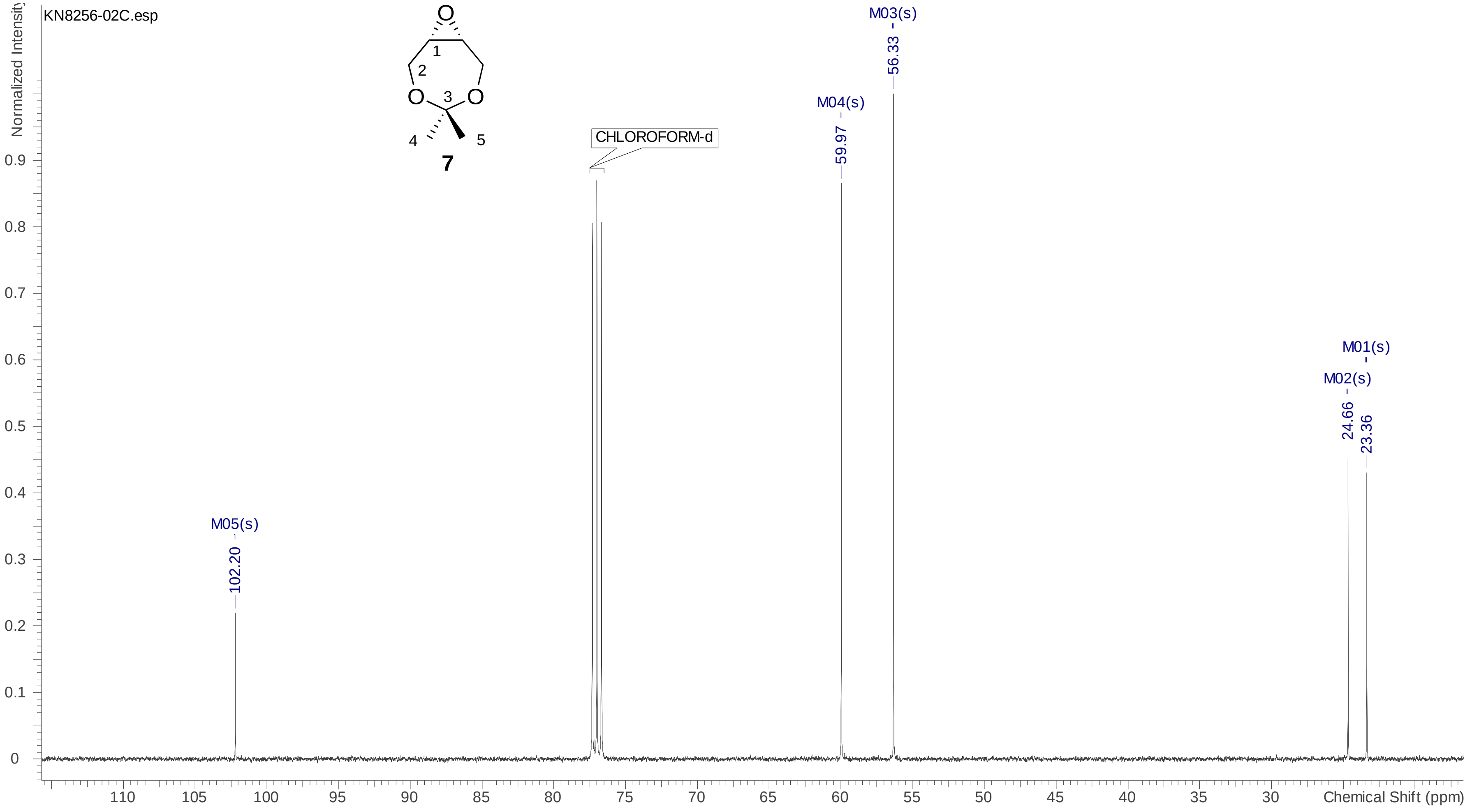

2.4 *syn*-2-Fluoro-3,4-*O*-isopropylidene butane-1,3,4-triol ((±)-9)

2.4.1 ^{1}H NMR (400 MHz, $CDCl_3$)

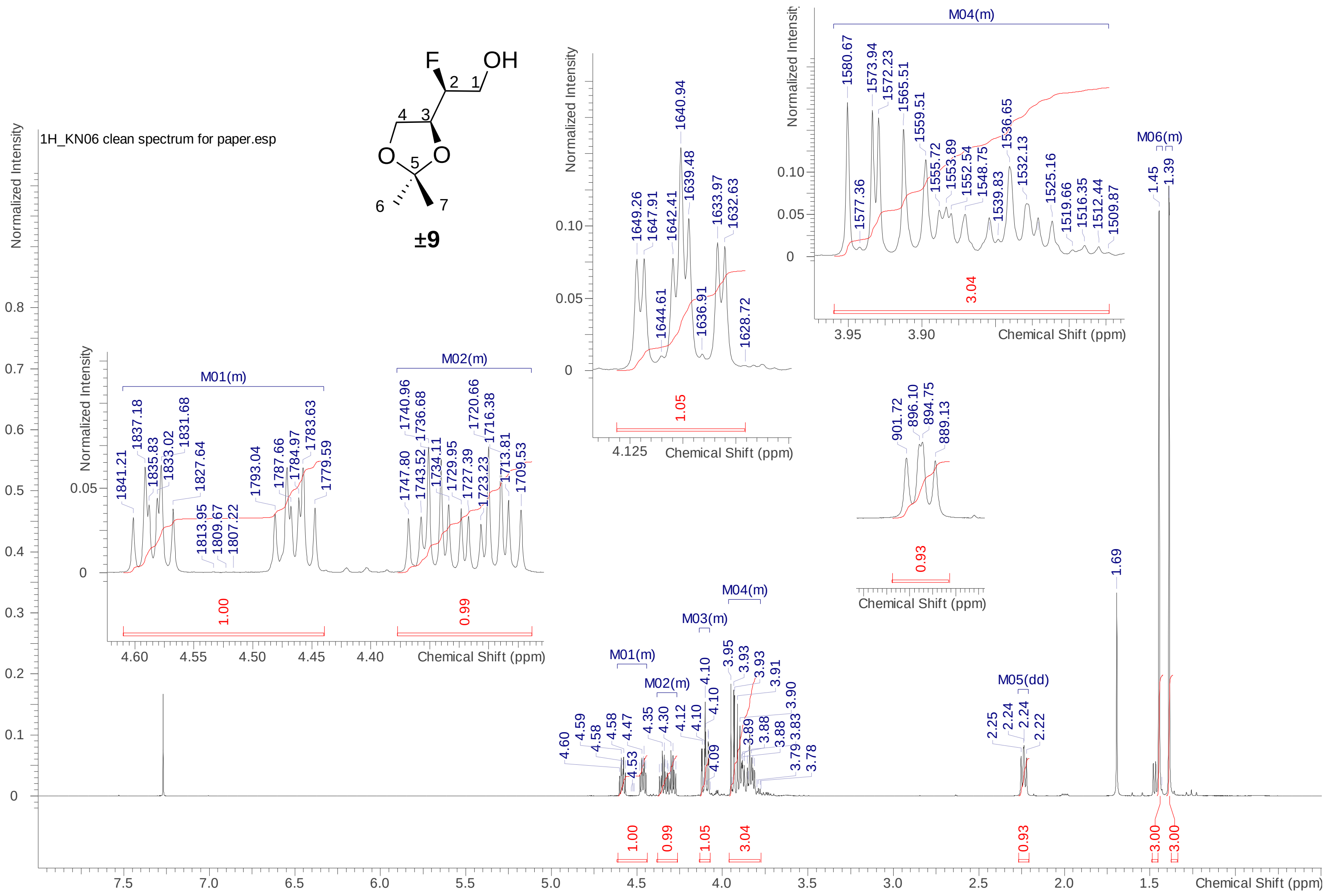

2.4.2 ^{1}H{OH} NMR (500 MHz, $CDCl_3$)(D_2O exchange experiment)

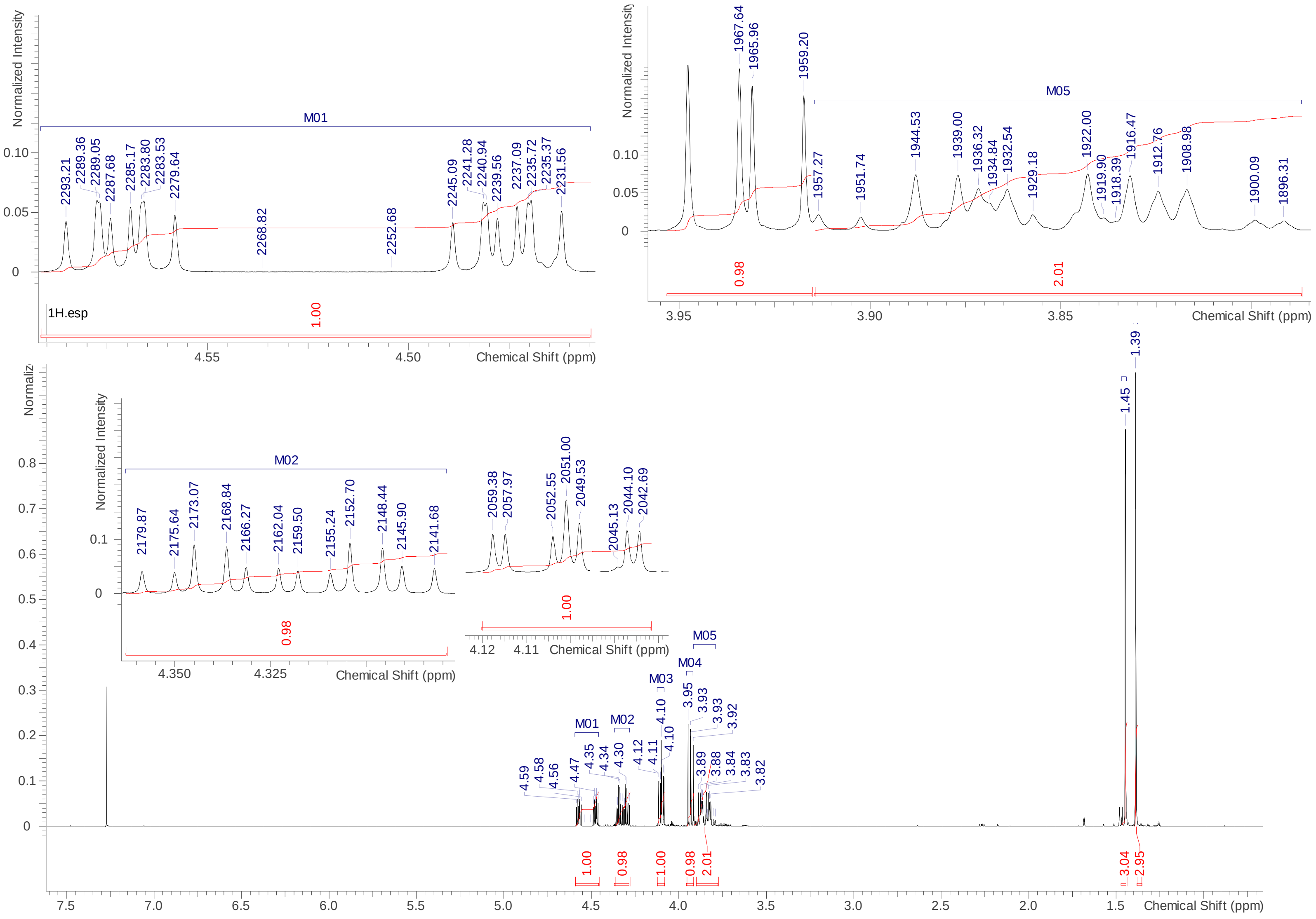

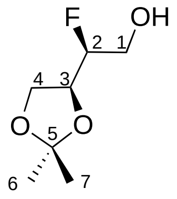

±9

2.4.3 $^{1}H\{^{19}F\}\{OH\}$ NMR (500 MHz, $CDCl_3$)(D_2O exchange experiment)

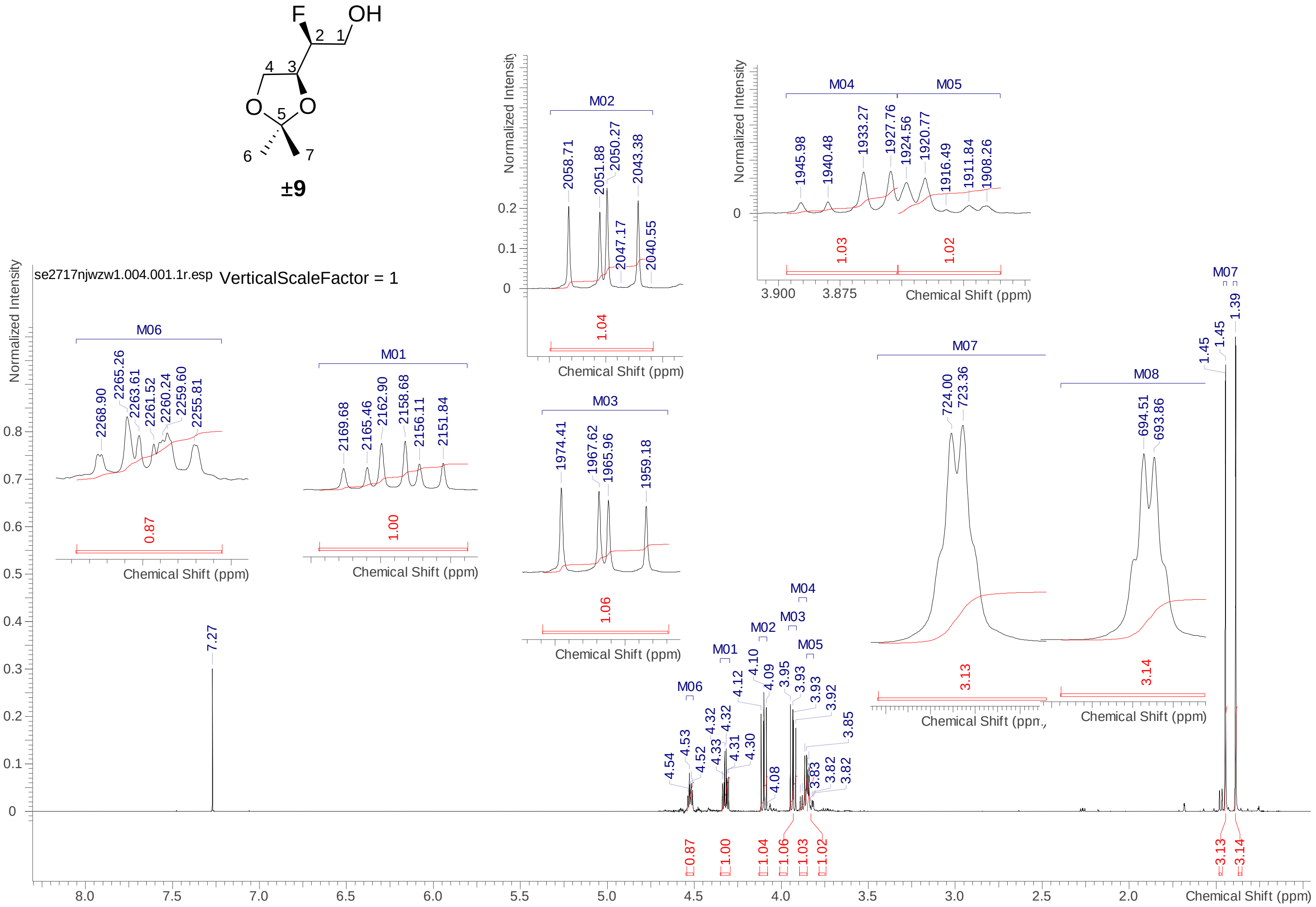

2.4.4 ^{19}F NMR of ±9 (471 MHz, $CDCl_3$)

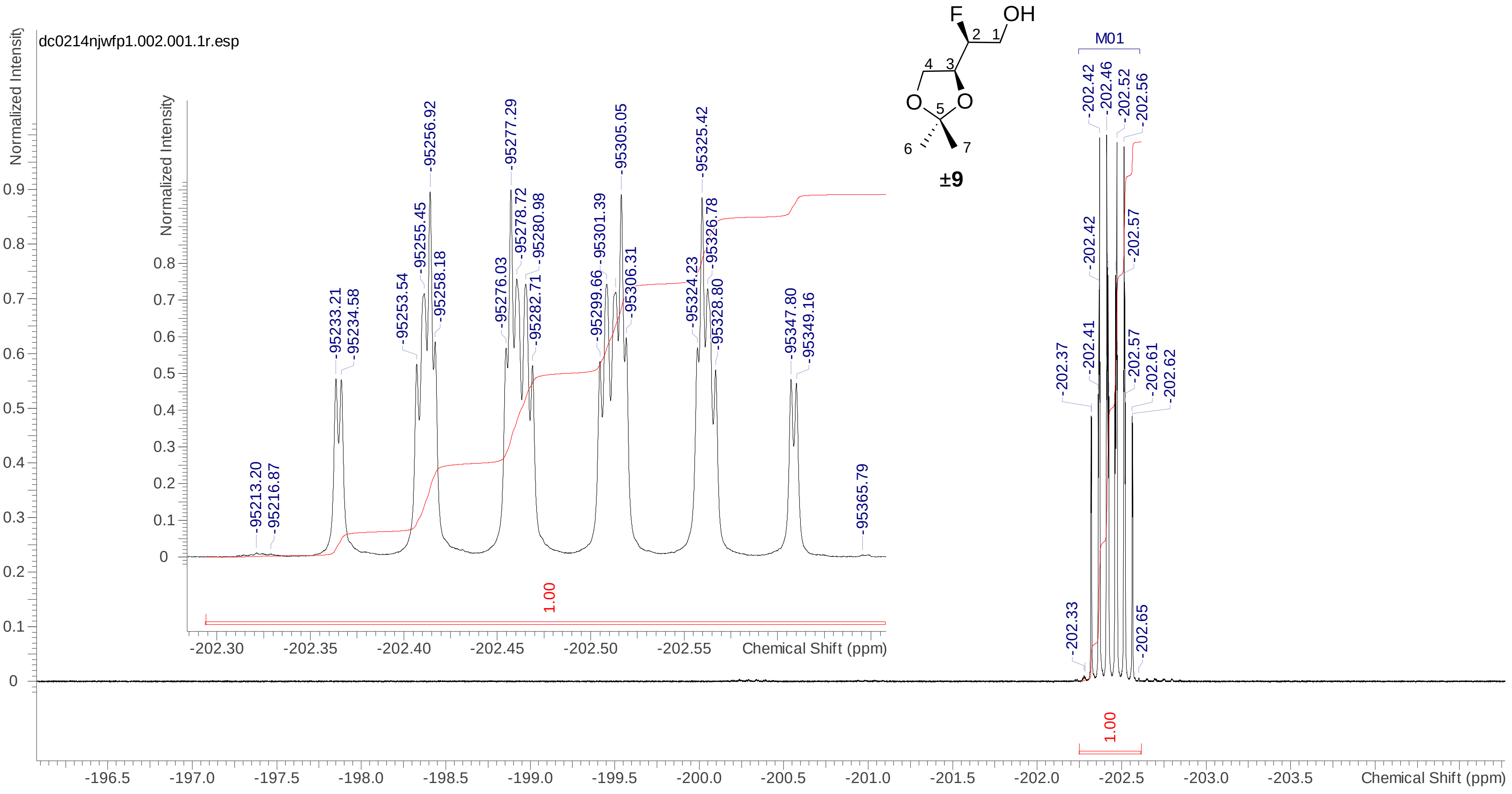

2.4.5 ^{13}C NMR (100 MHz, $CDCl_3$)

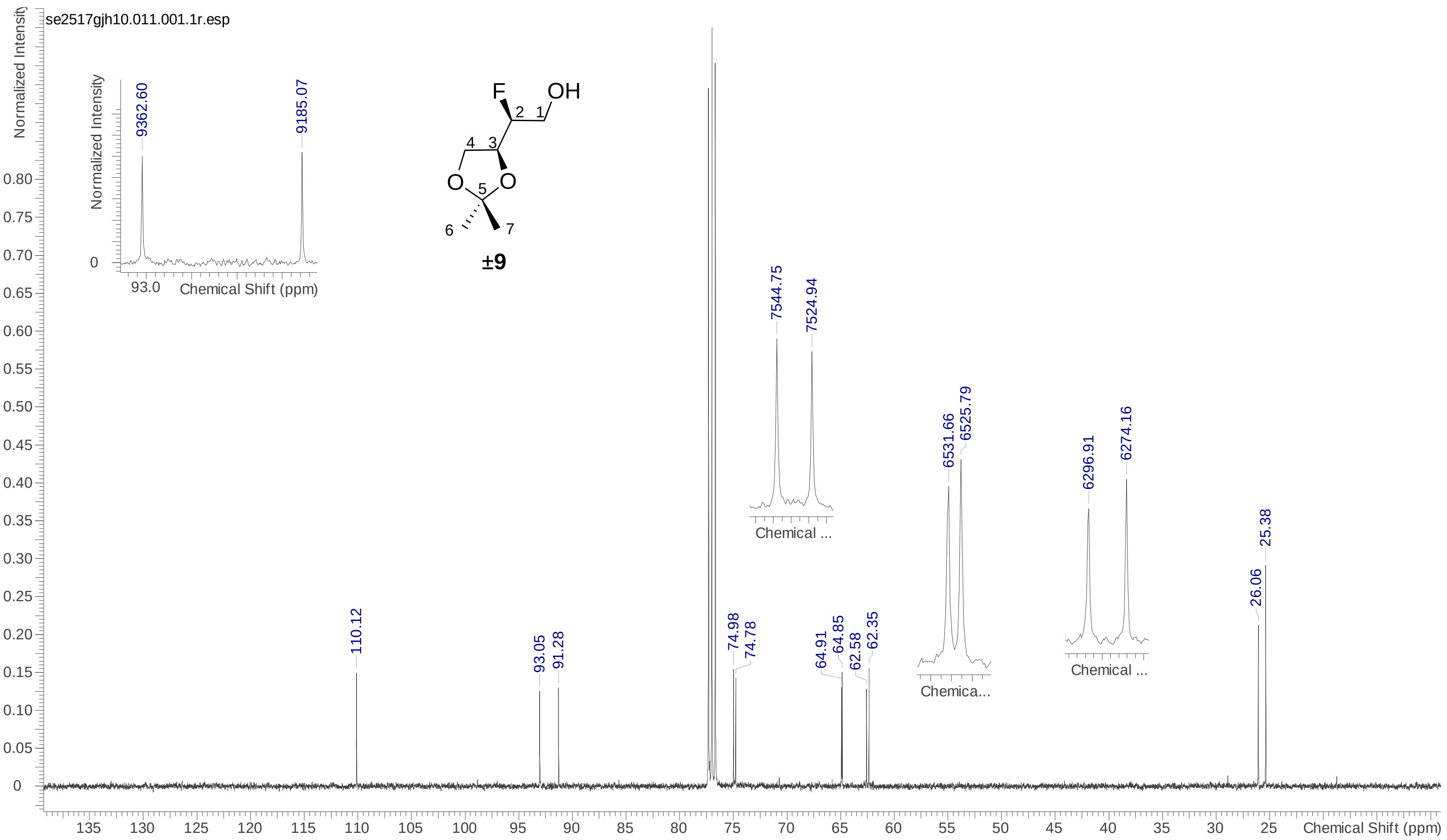

2.5 4-(1,2-Difluoroethyl)-2,2-dimethyl-1,3-dioxolane ((±)-10)

2.5.1 ^{1}H NMR (500 MHz, $CDCl_3$)

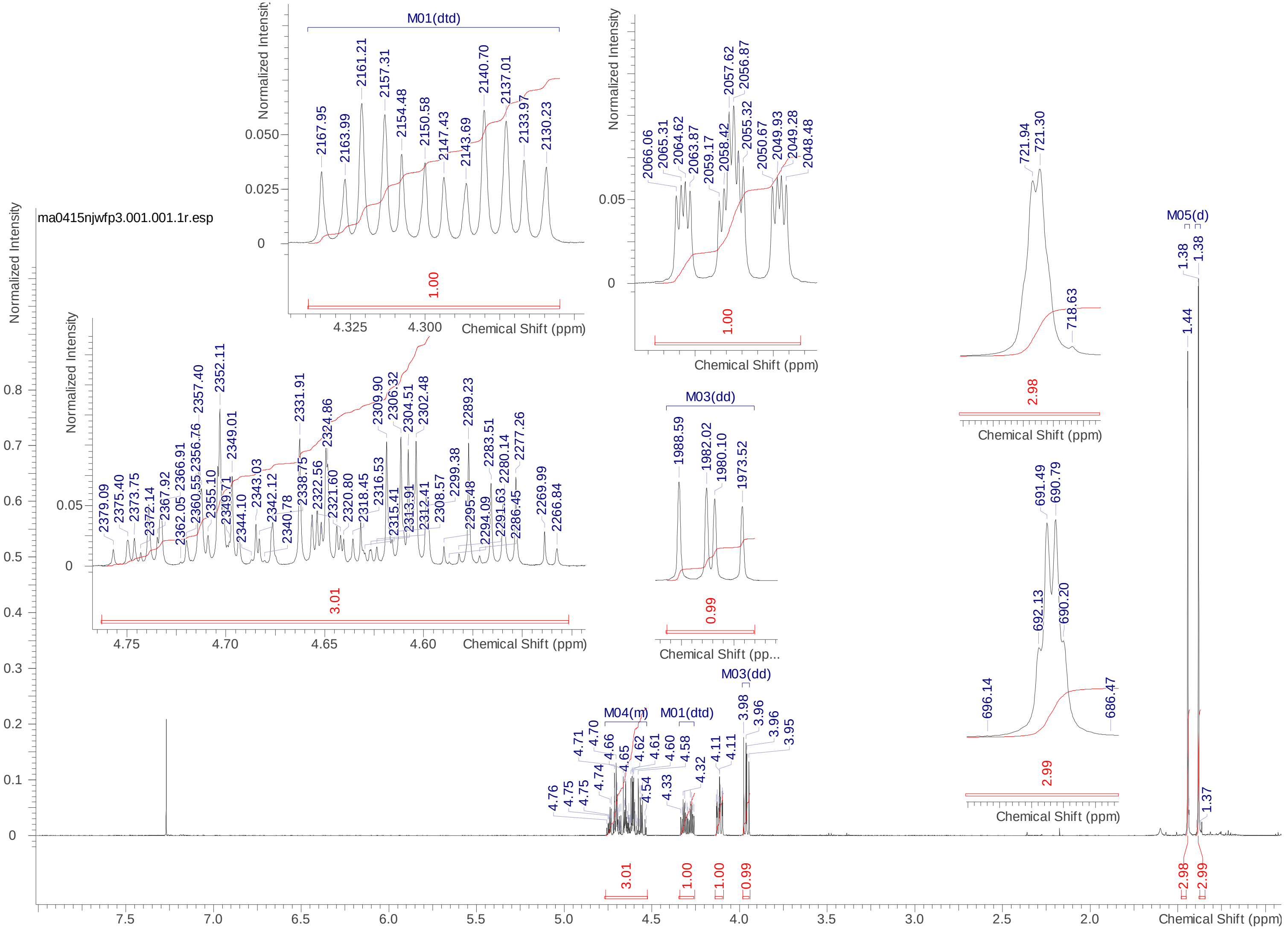

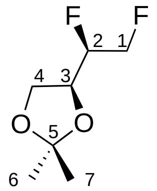

±10

2.5.2 $^{1}H\{^{19}F\}$ NMR (500 MHz, $CDCl_3$)

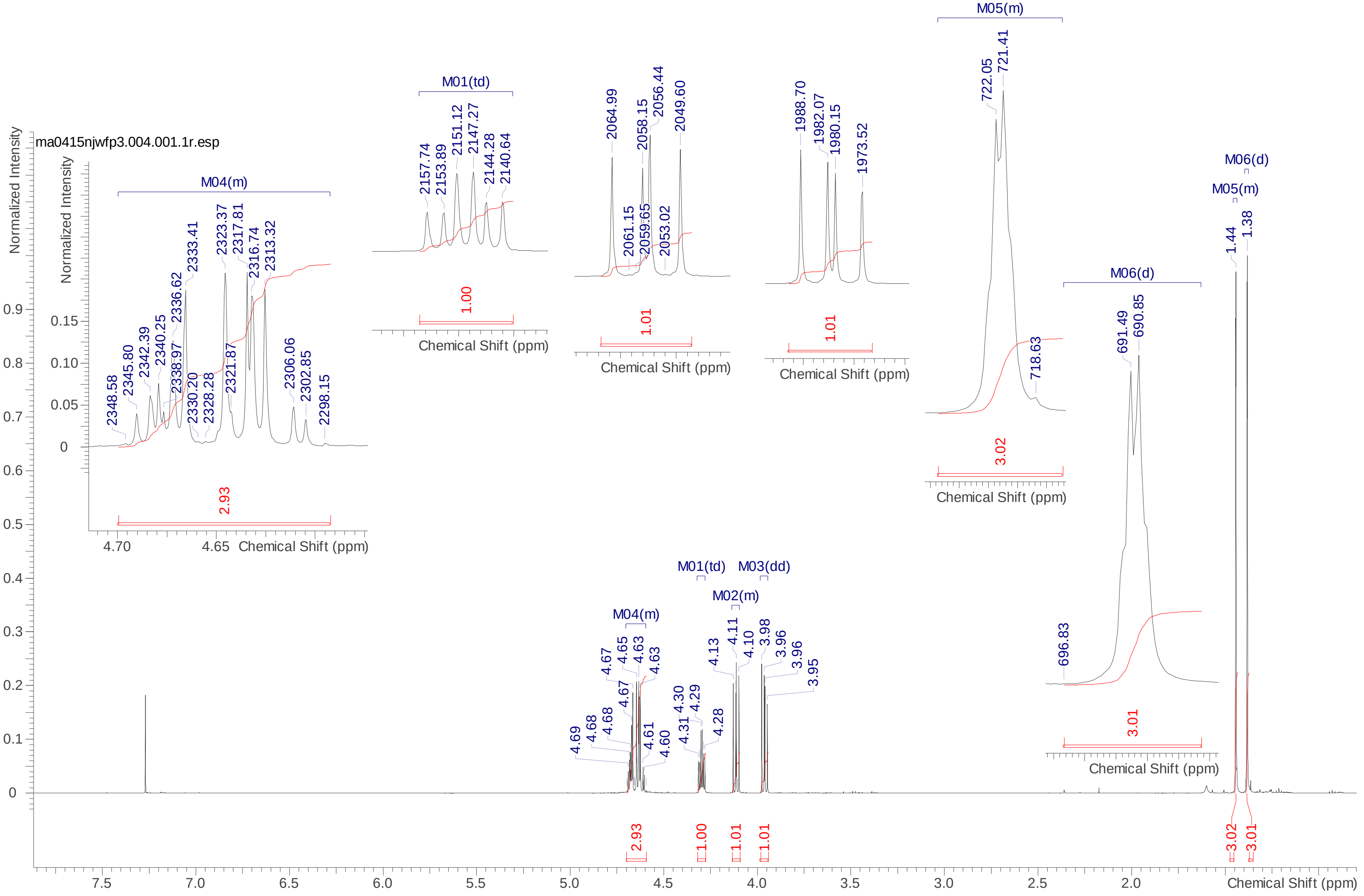

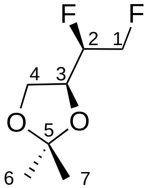

±10

2.5.3 ^{19}F NMR (471 MHz, $CDCl_3$)

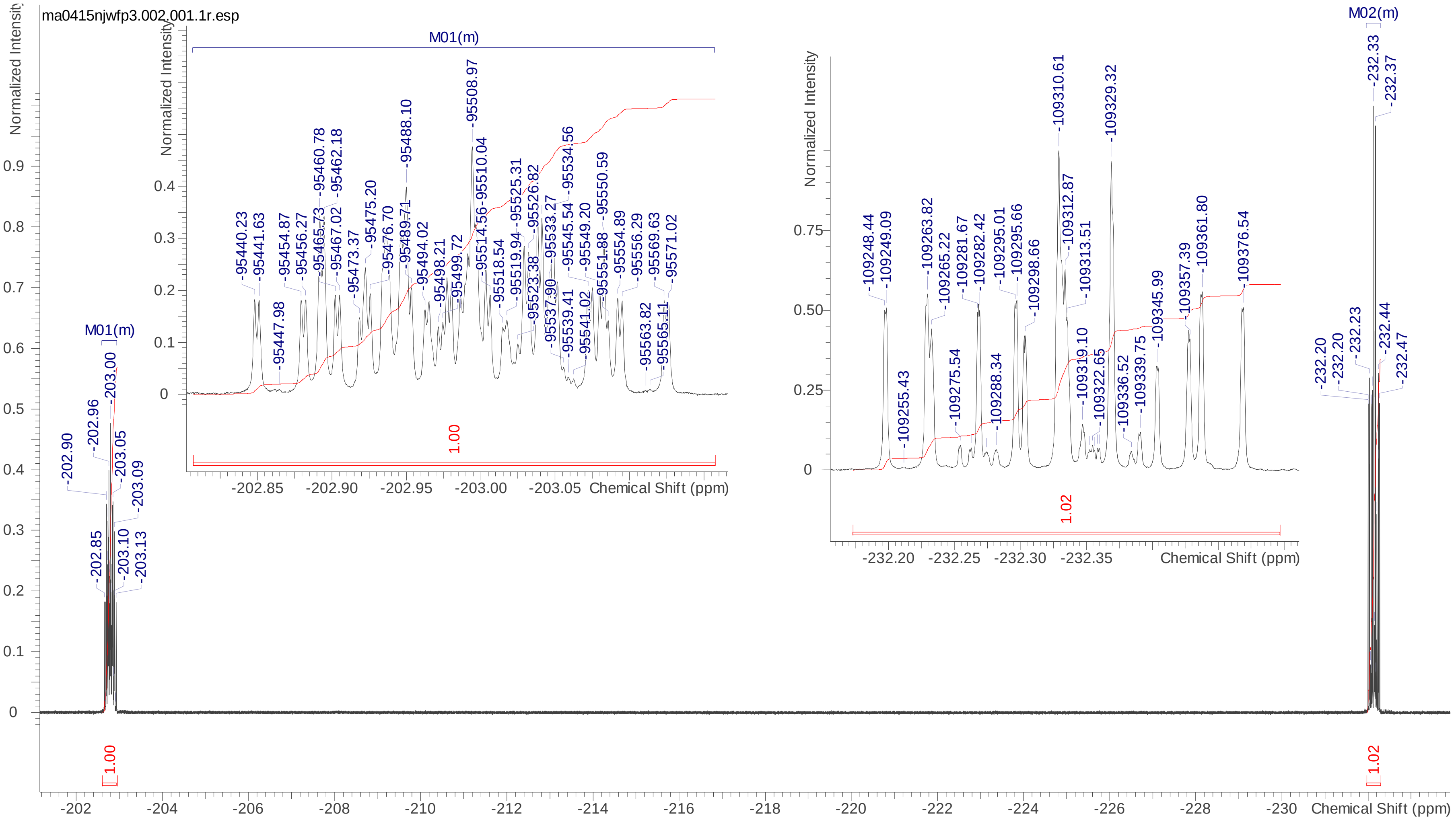

2.5.4 $^{19}F\{^1H\}$ (471 MHz, $CDCl_3$)

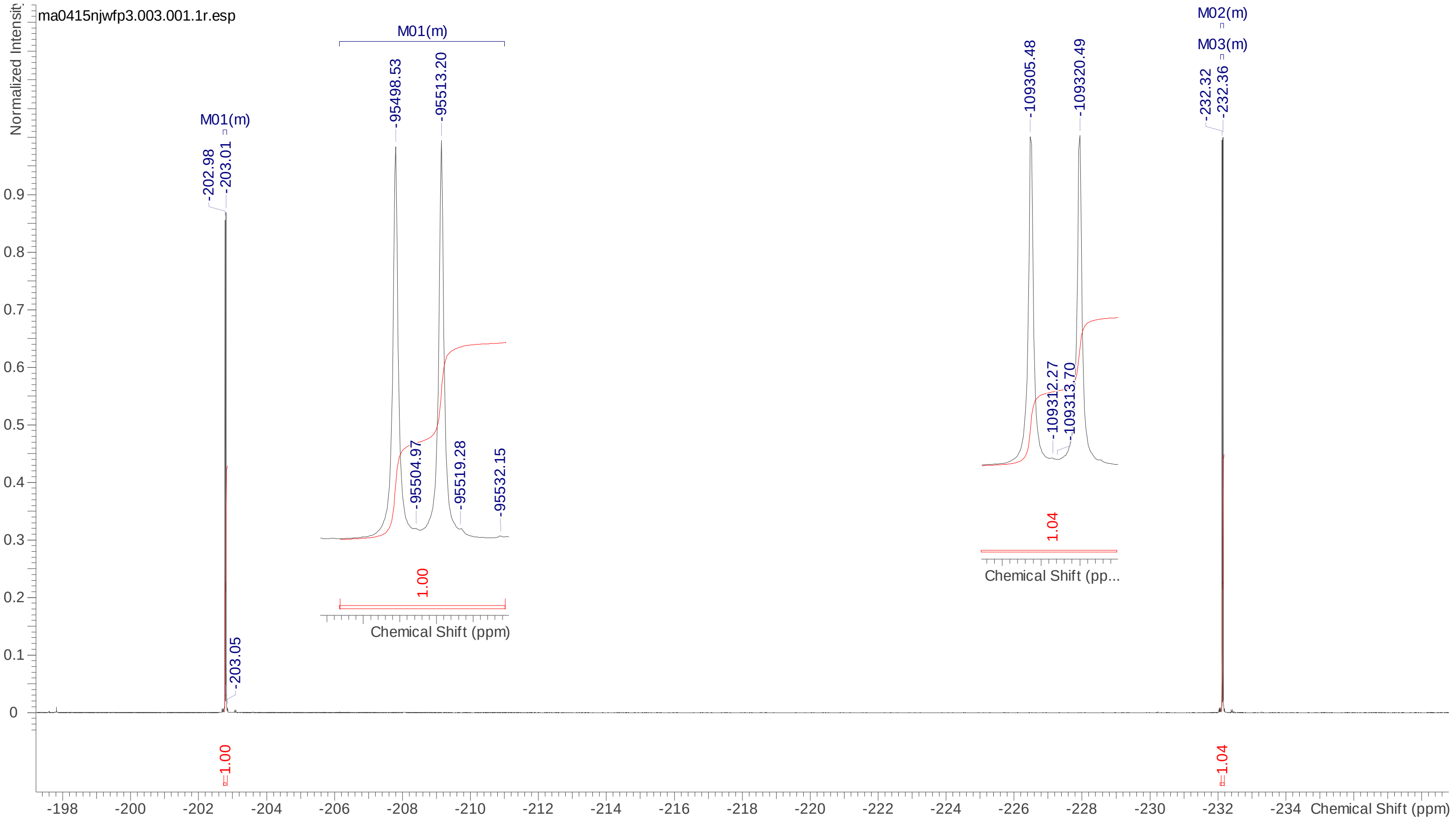

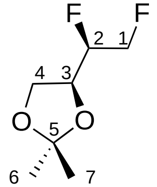

±10

2.5.5 ^{13}C NMR (100 MHz, $CDCl_3$)

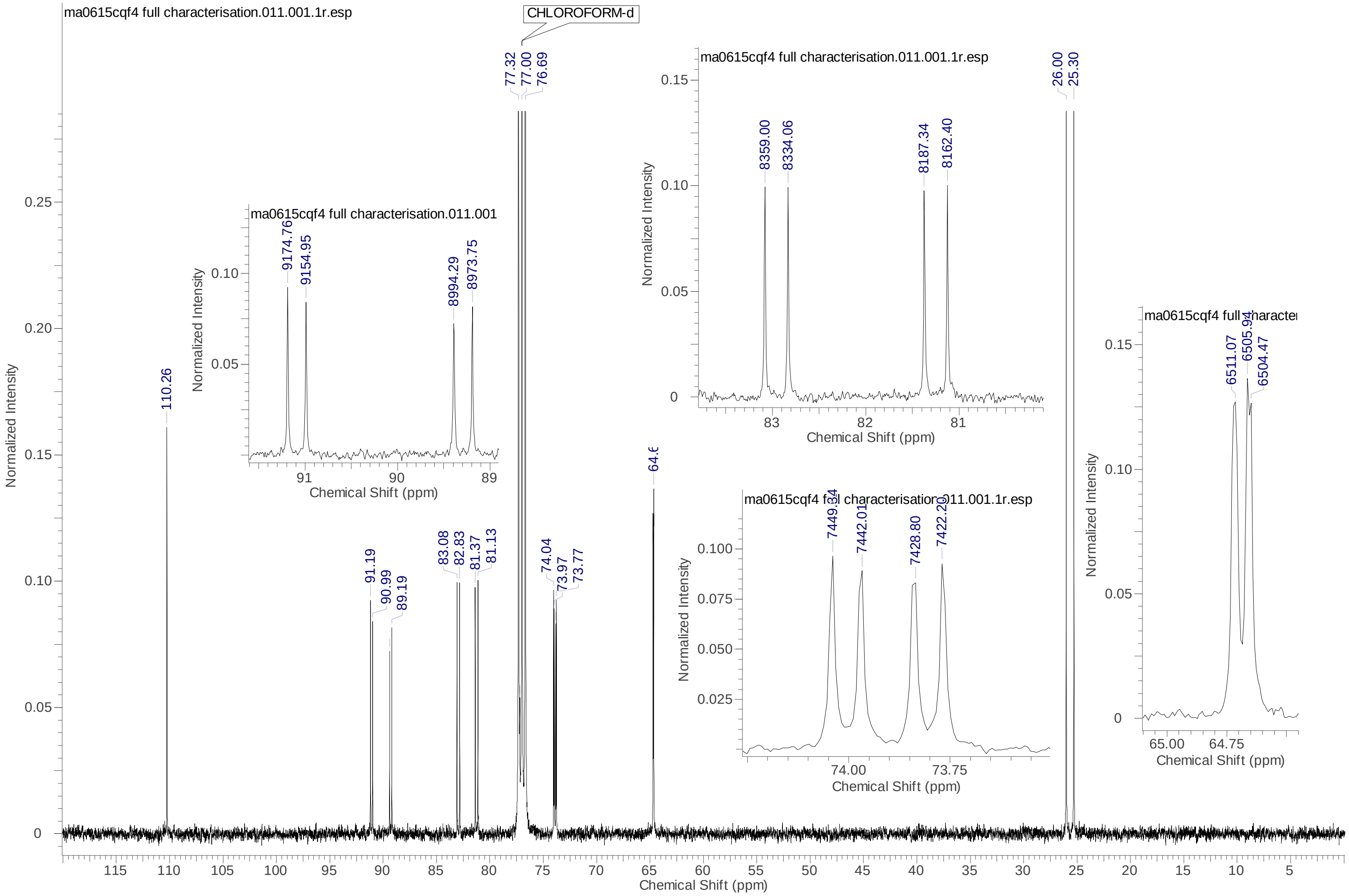

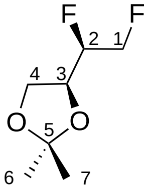

±10

2.5.6 DEPT-135 (100 MHz, $CDCl_3$)

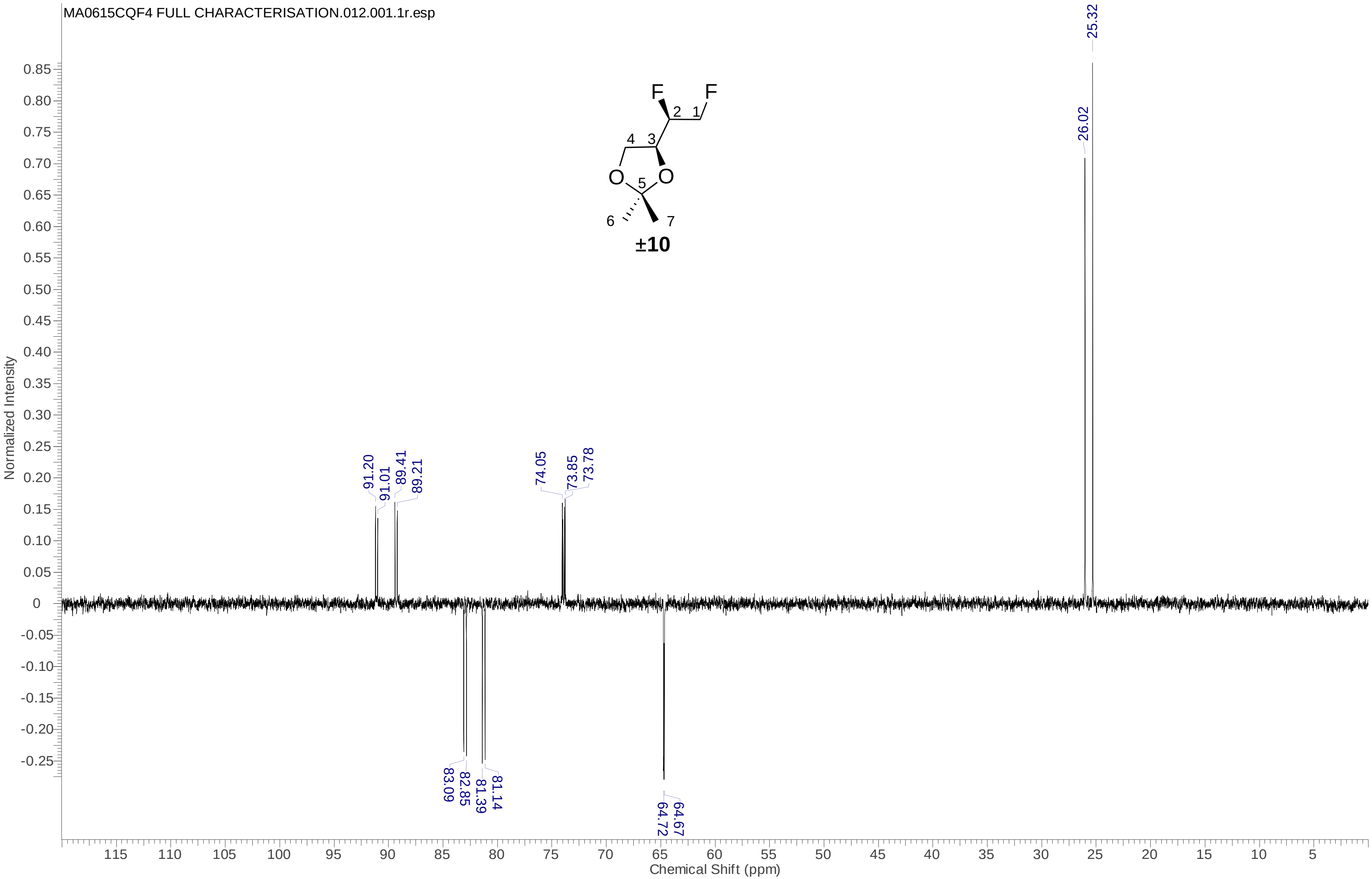

2.6 6-Fluoro-2,2-dimethyl-1,3-dioxepan-5-ol ((±)-8)

2.6.1 ^{1}H NMR (400 MHz, $CDCl_3$)

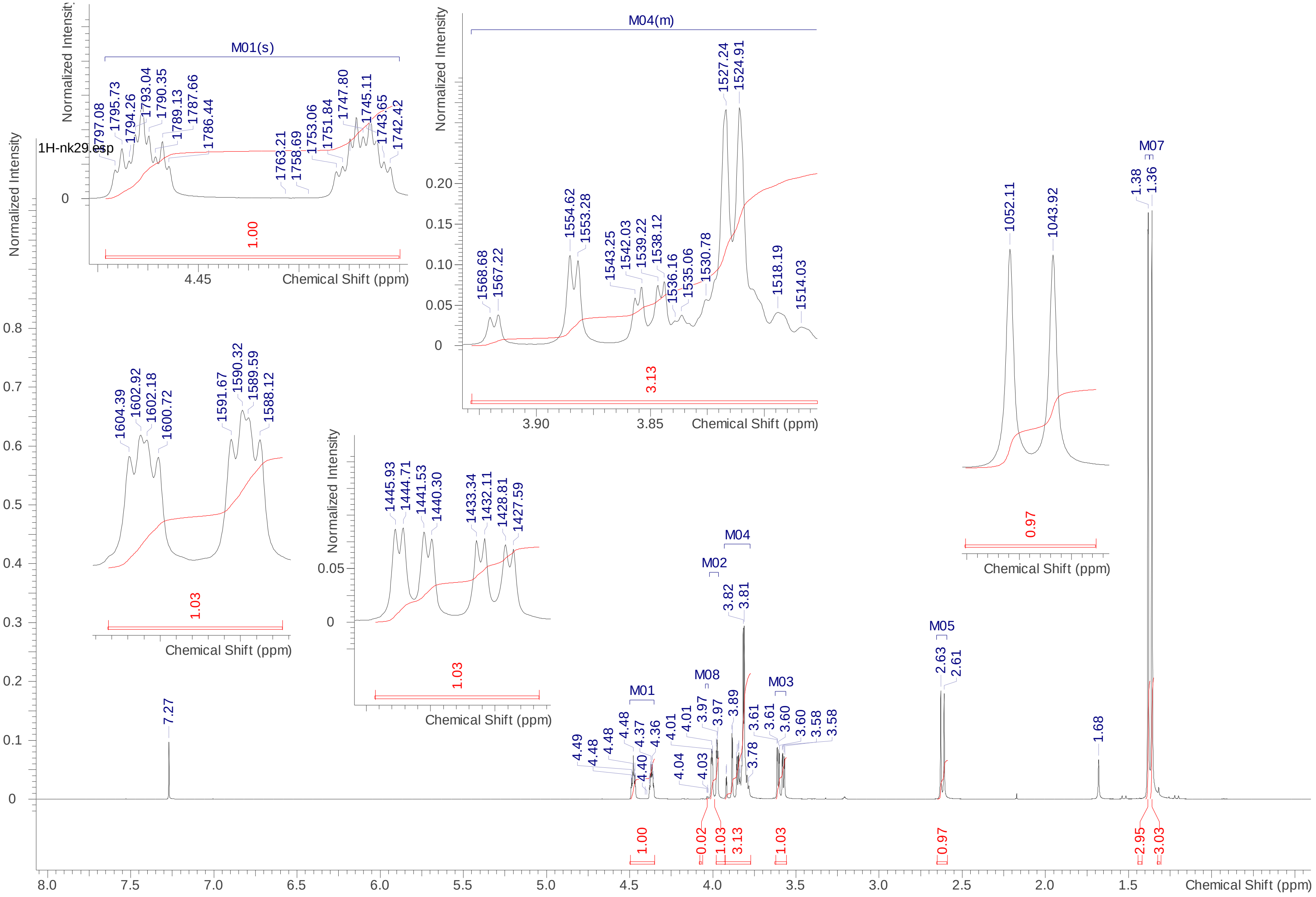

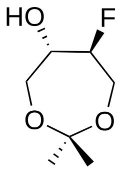

±8

2.6.2 ^{13}C NMR (100 MHz, $CDCl_3$)

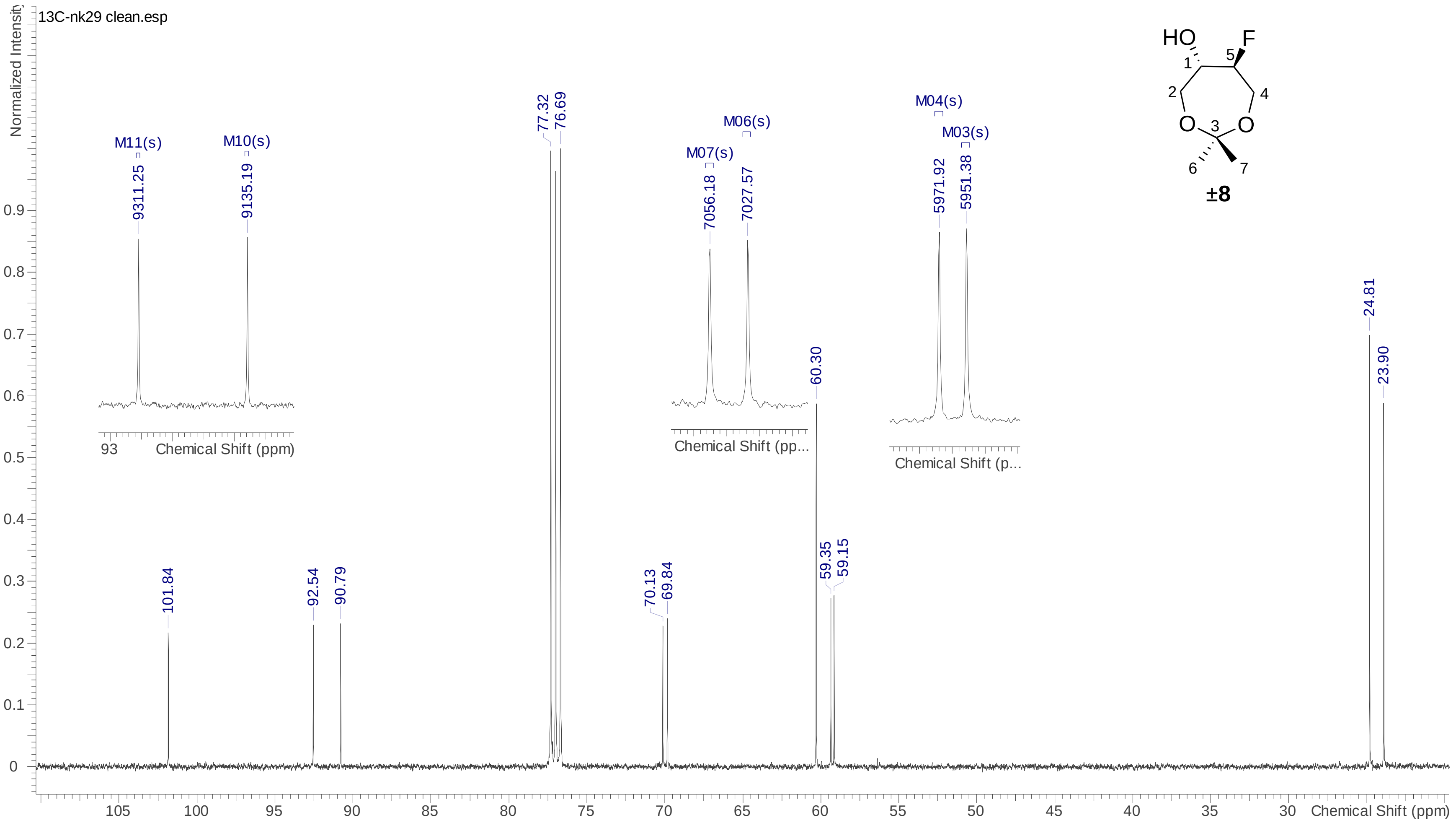

2.6.3 ^{19}F NMR (376 MHz, $CDCl_3$)

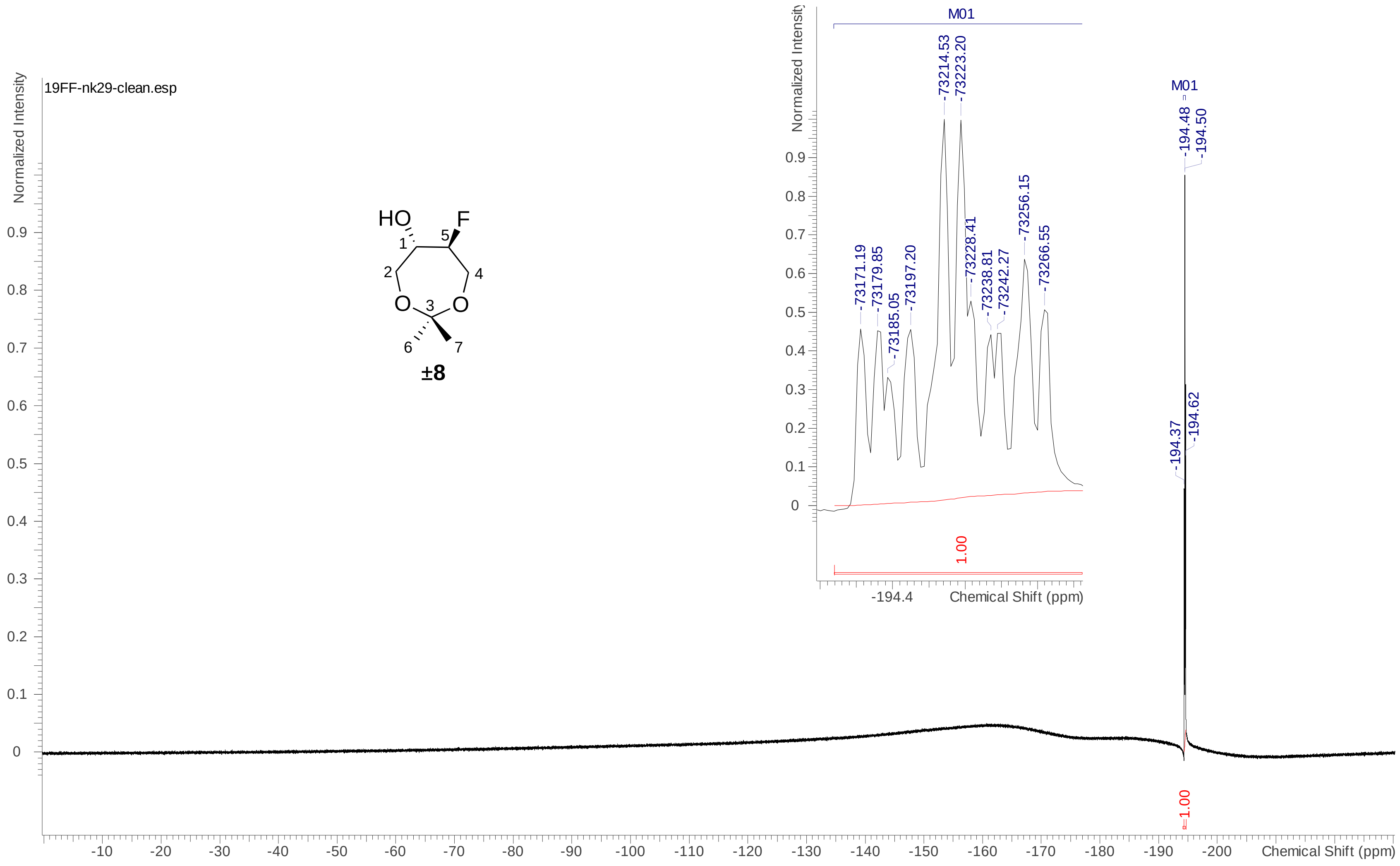

2.7 Sulfonate byproduct (±)-12

2.7.1 ^{1}H NMR (400 MHz, $CDCl_3$)

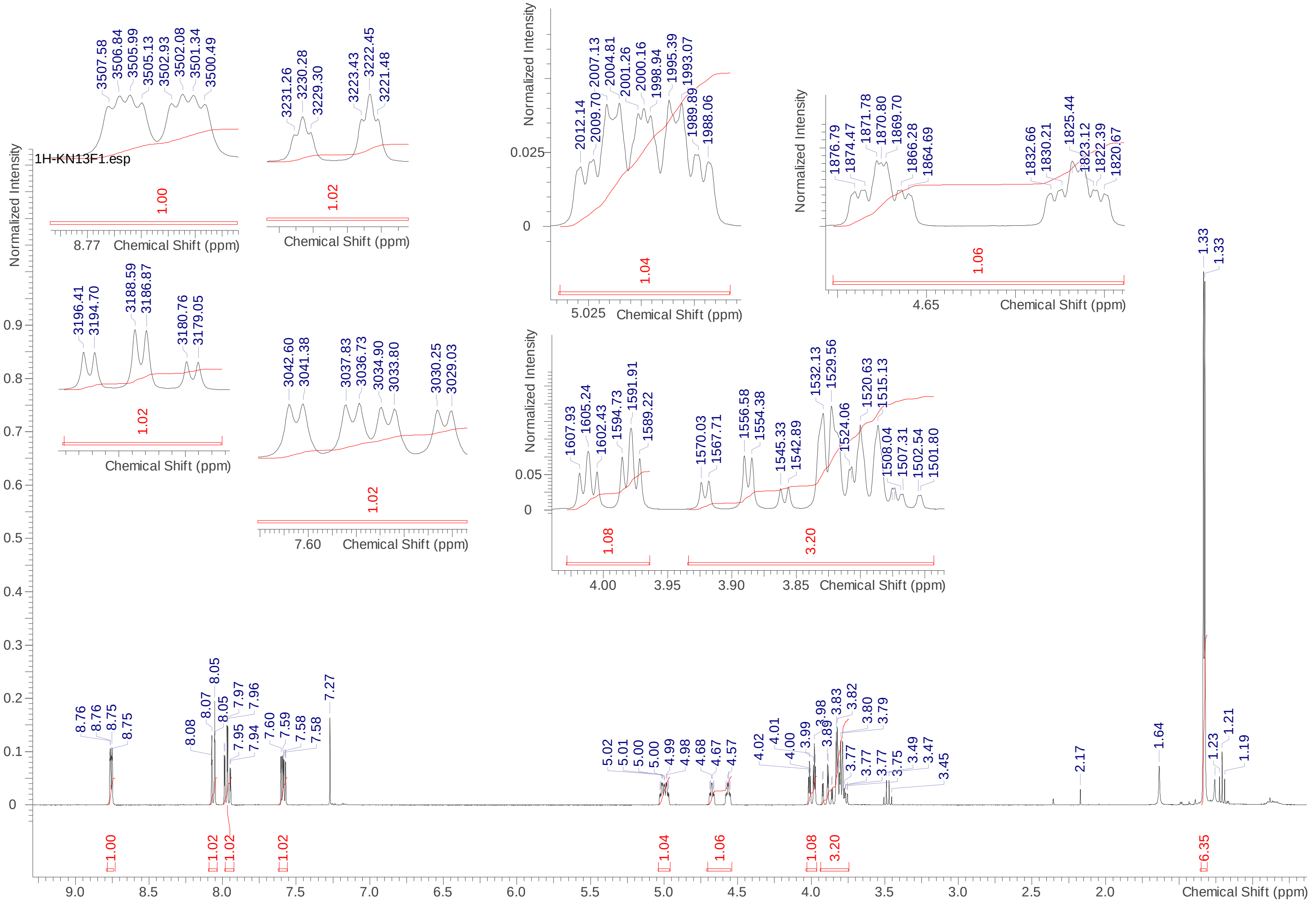

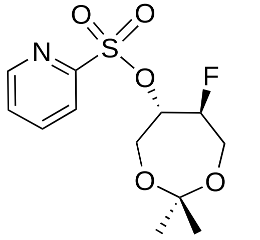

±12

2.7.2 ^{13}C NMR (100 MHz, $CDCl_3$)

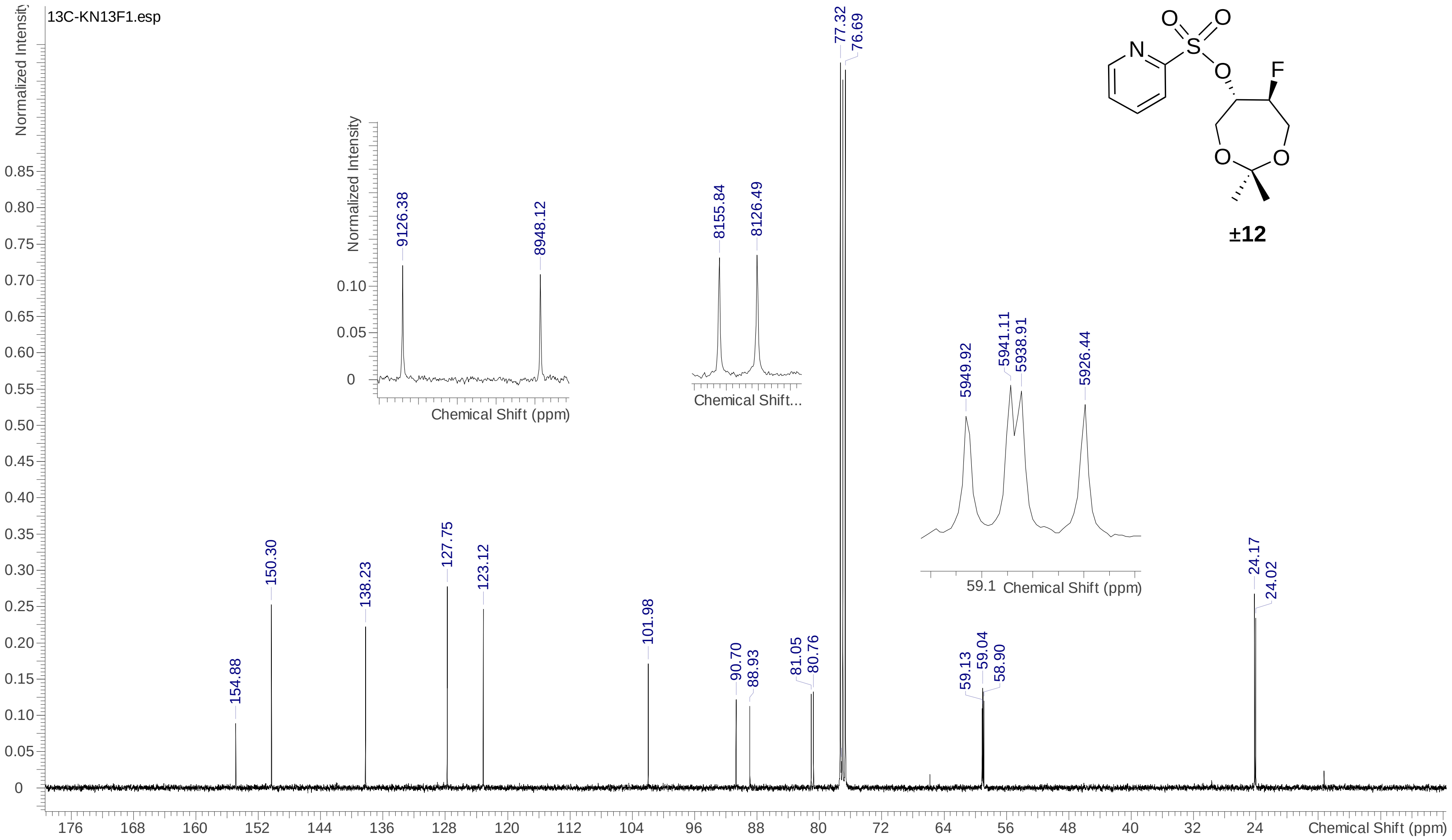

±12

2.7.3 ^{19}F NMR (376 MHz, $CDCl_3$)

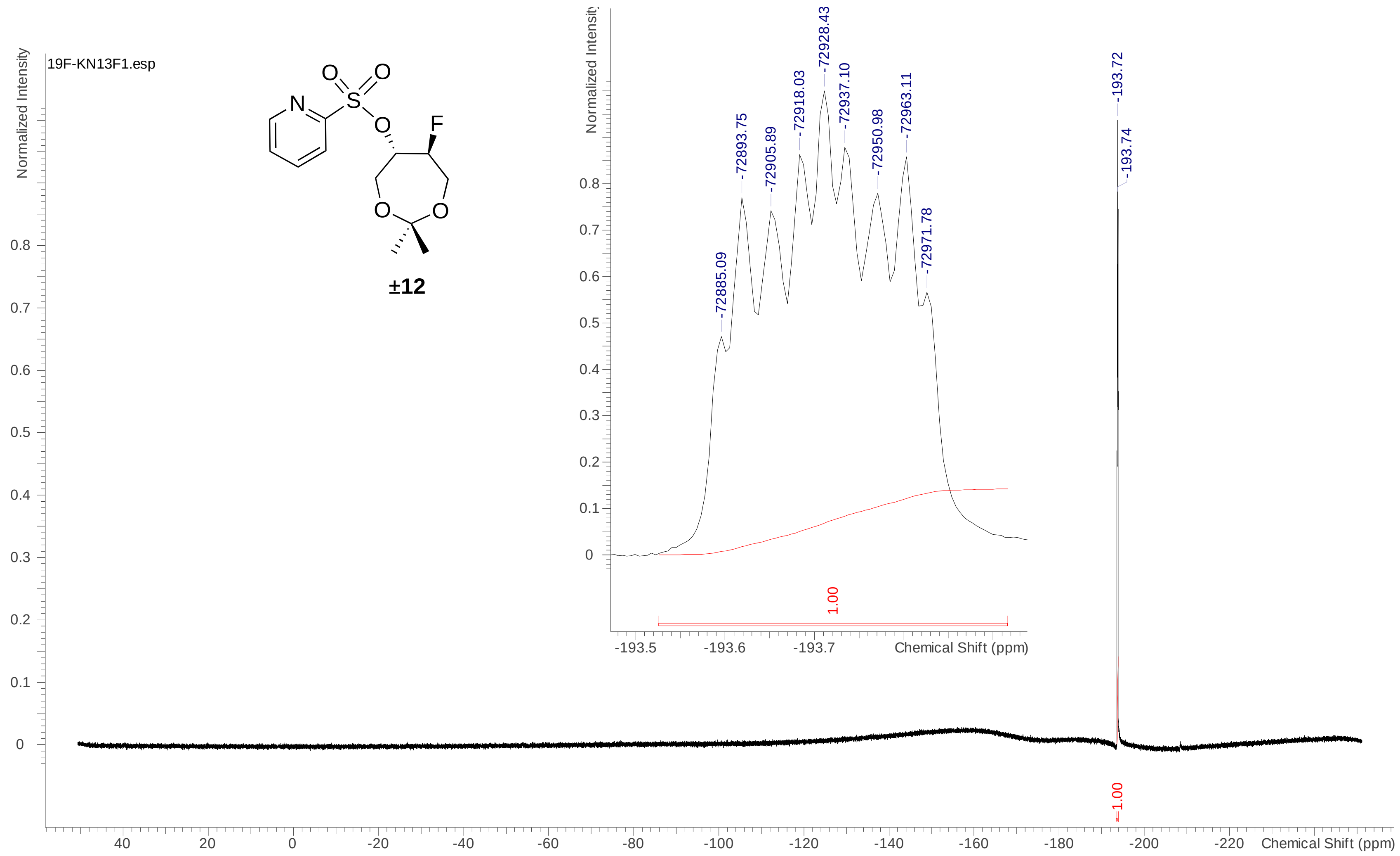

2.8 *meso*-5,6-Difluoro-2,2-dimethyl-1,3-dioxepane (*meso*-11)

2.8.1 ^{1}H NMR (400 MHz, $CDCl_3$)

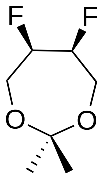

meso-**11**

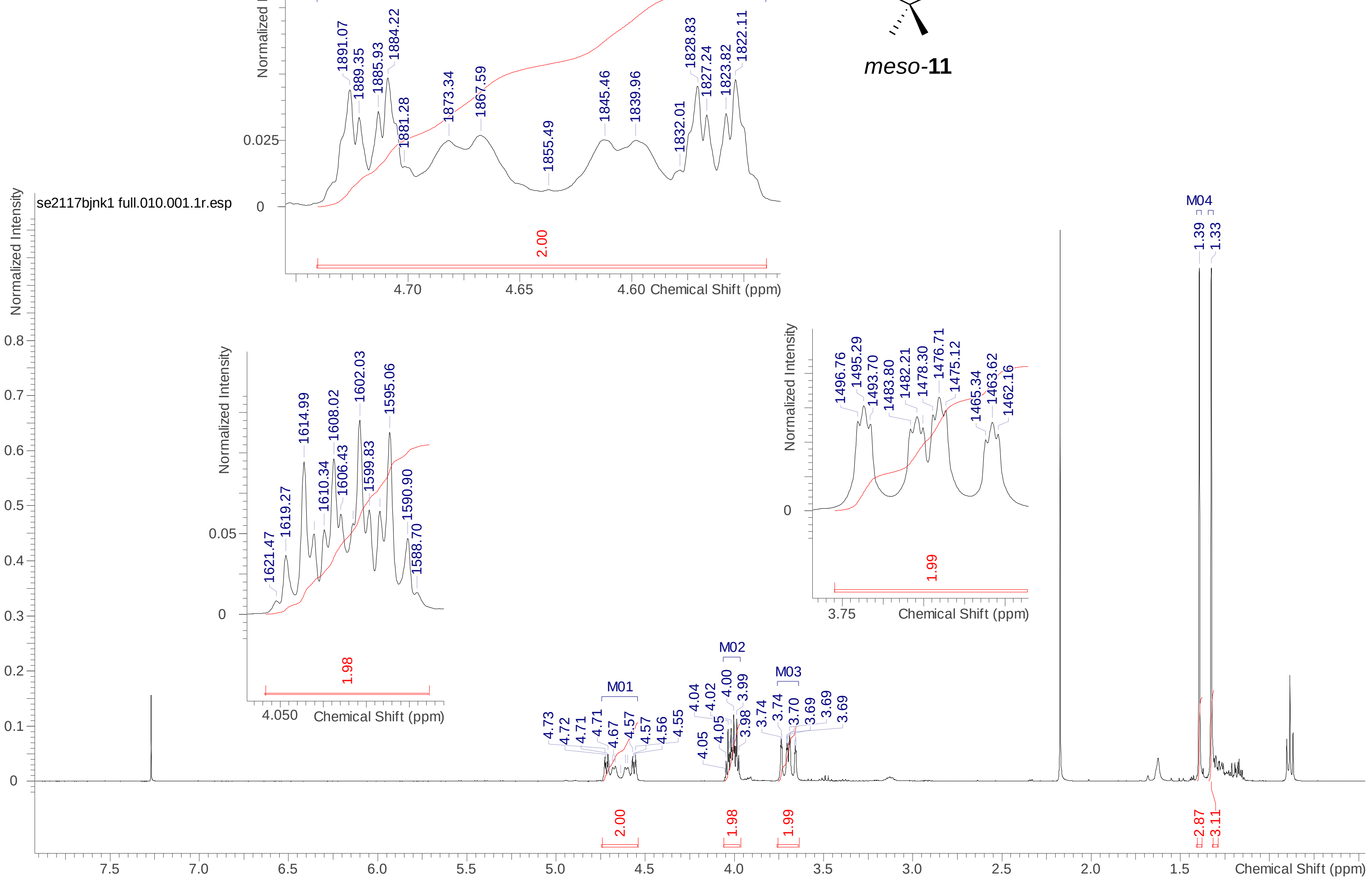

2.8.2 ^{19}F NMR (376 MHz, $CDCl_3$)

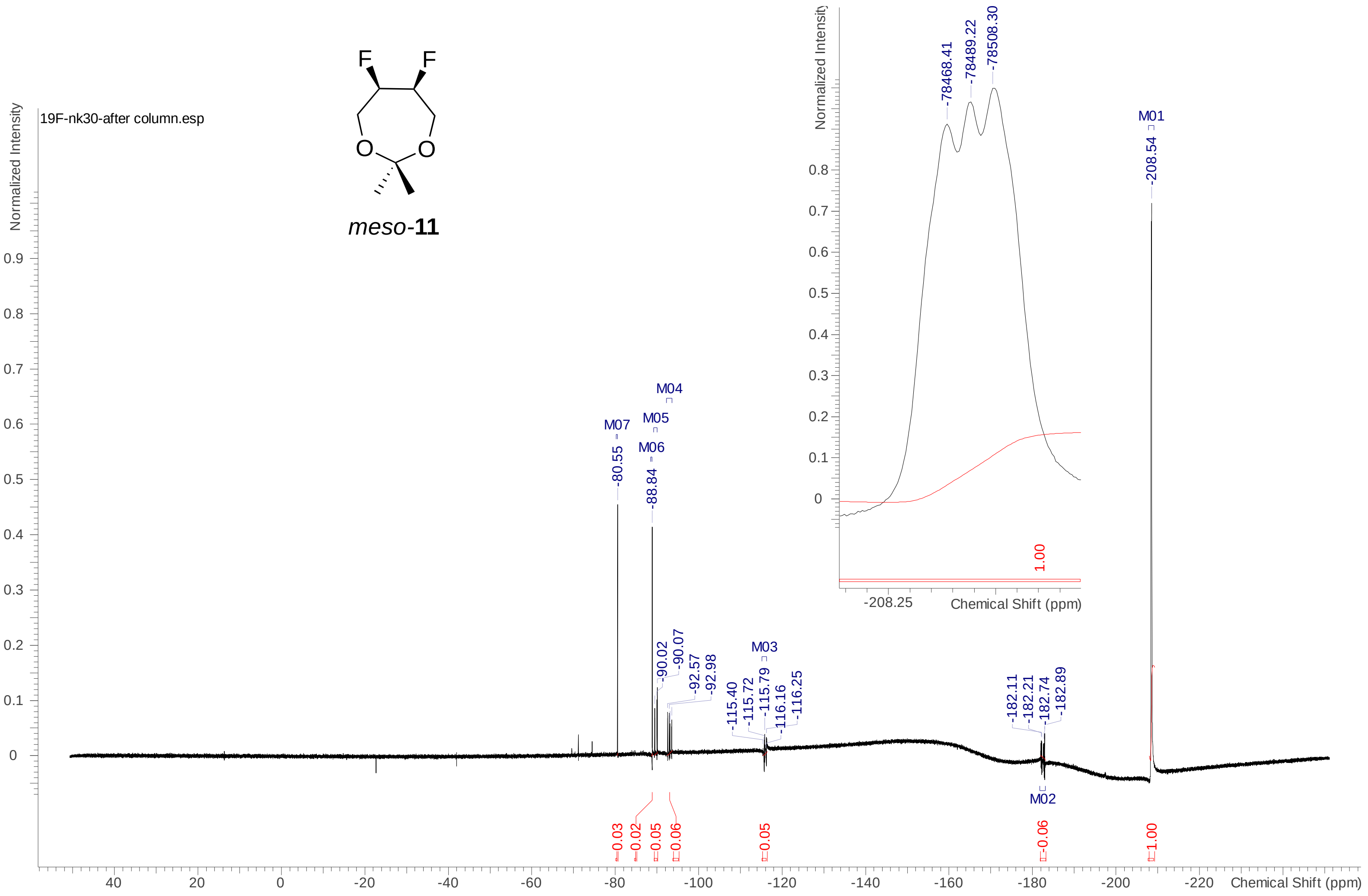

2.8.3 ^{13}C NMR (100 MHz, $CDCl_3$)

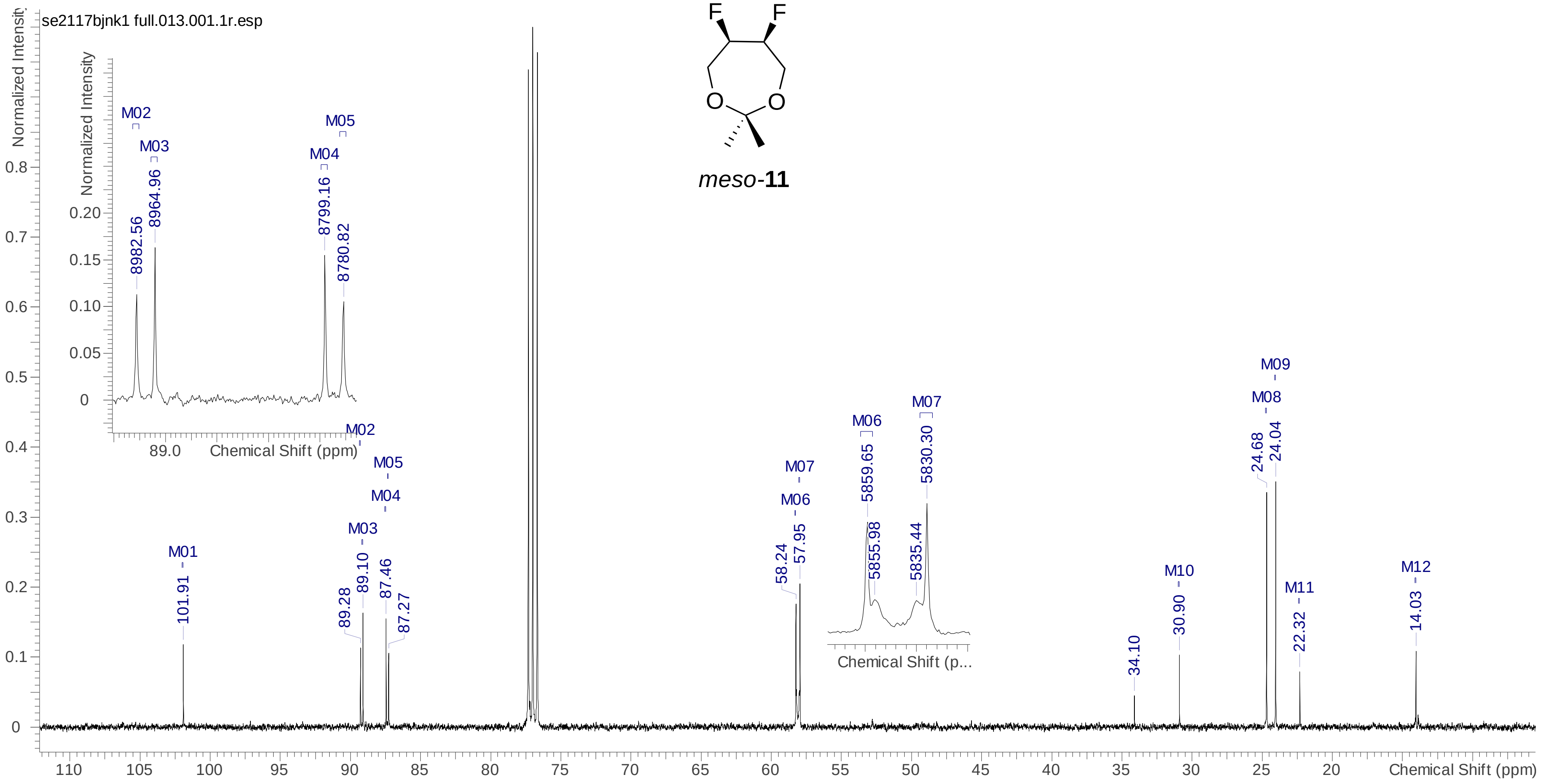

meso-**11**

2.9 *anti*-2,3-Difluorobutane-1,4-diol (*anti*-5)

2.9.1 ^{1}H NMR (400 MHz, $CDCl_3$)

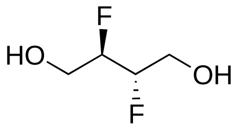

anti-**5**

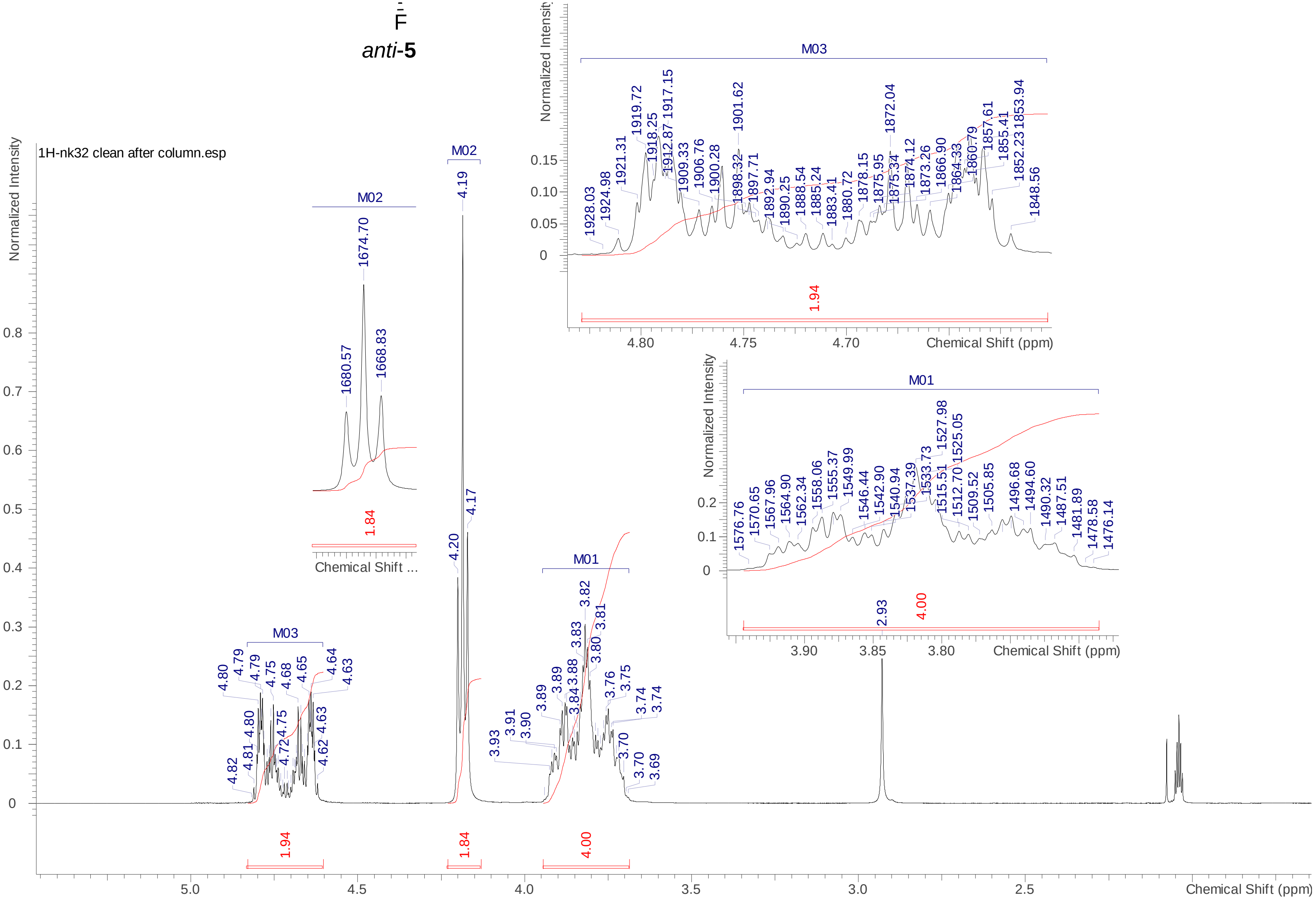

2.9.2 ^{19}F NMR (376 MHz, $CDCl_3$)

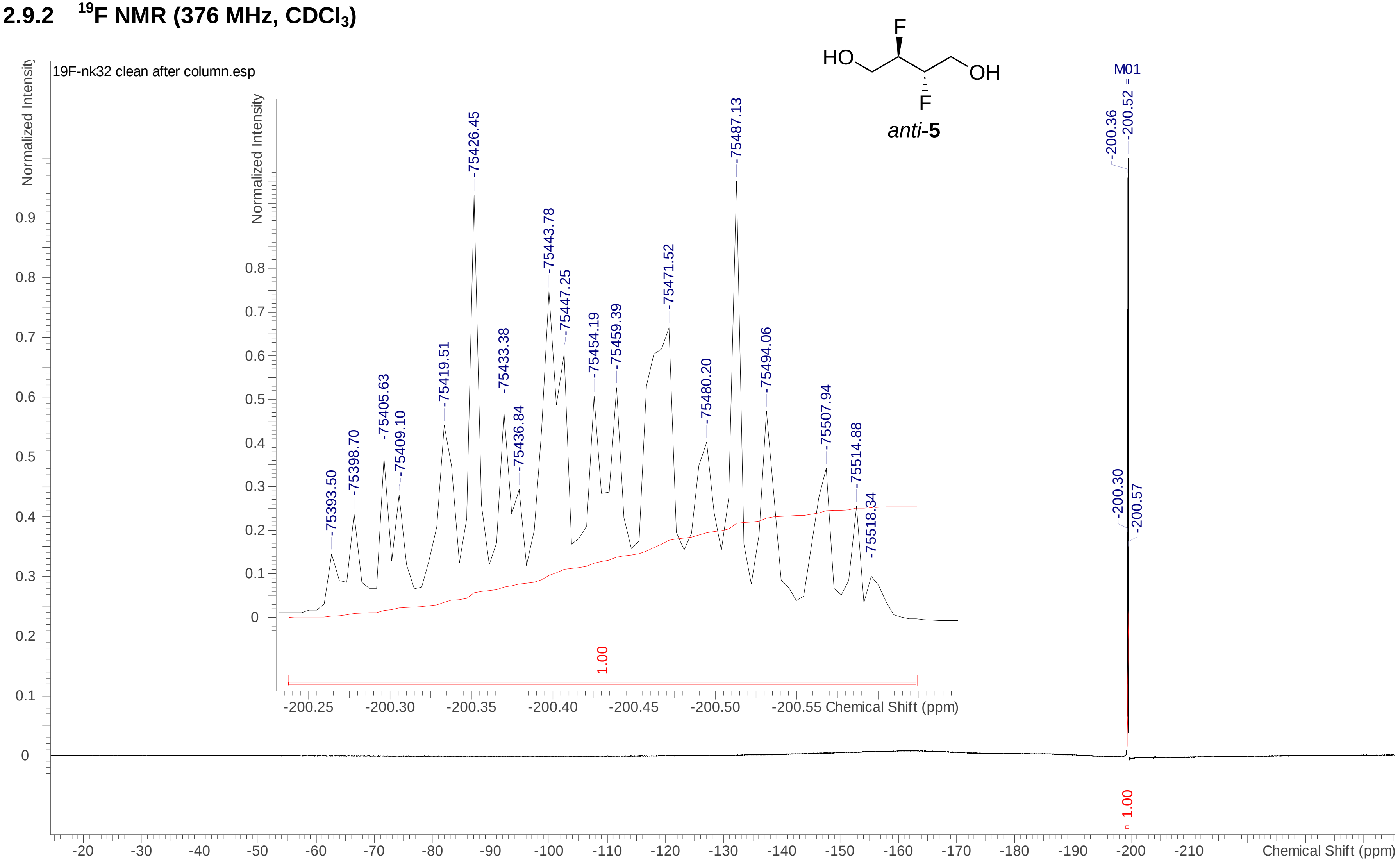

2.10 *(E)*-But-2-ene-1,4-diol (*trans*-1)

2.10.1 ^{1}H NMR (400 MHz, $CDCl_3$)

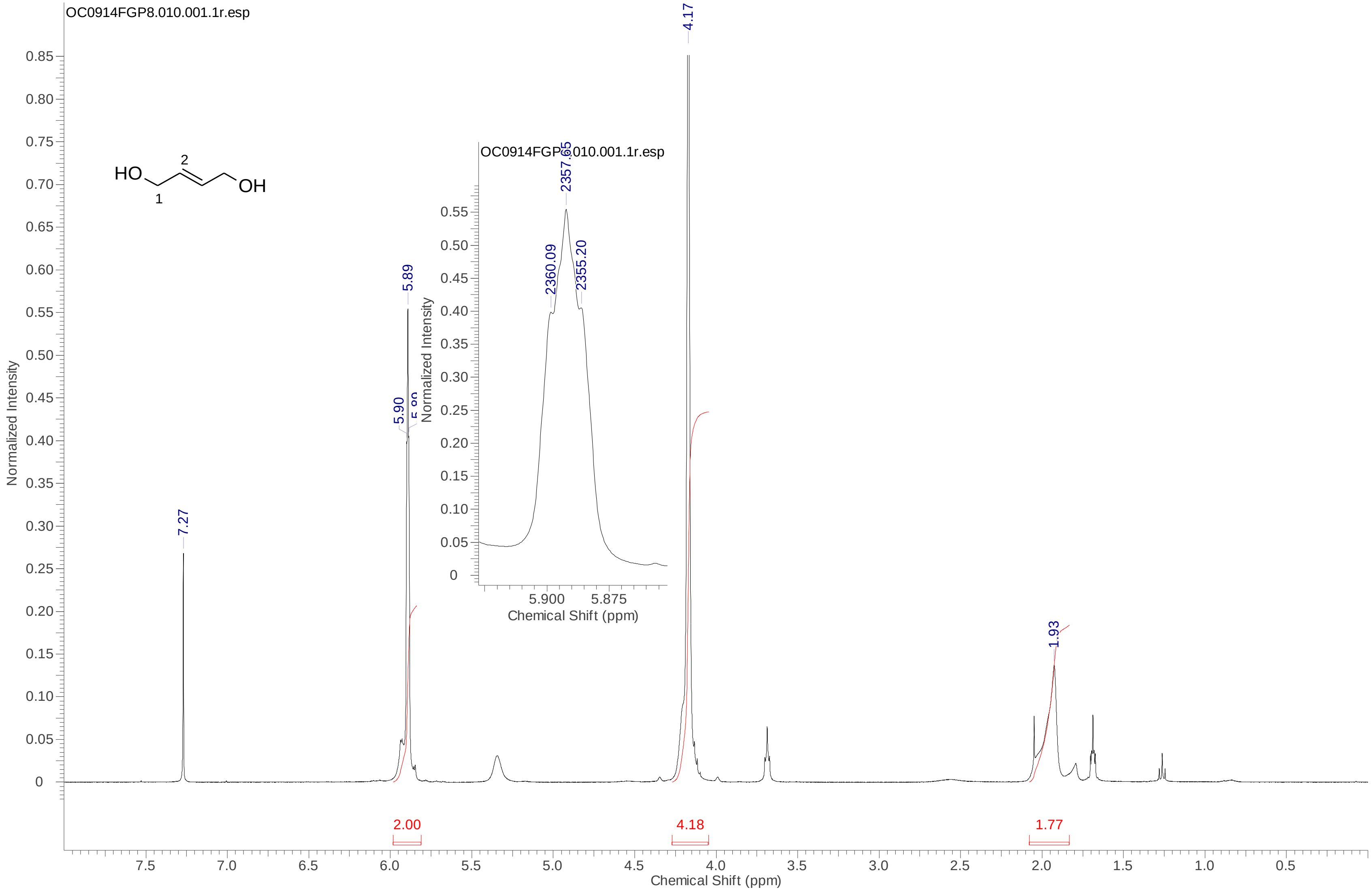

2.10.2 ^{13}C NMR (100 MHz, $CDCl_3$)

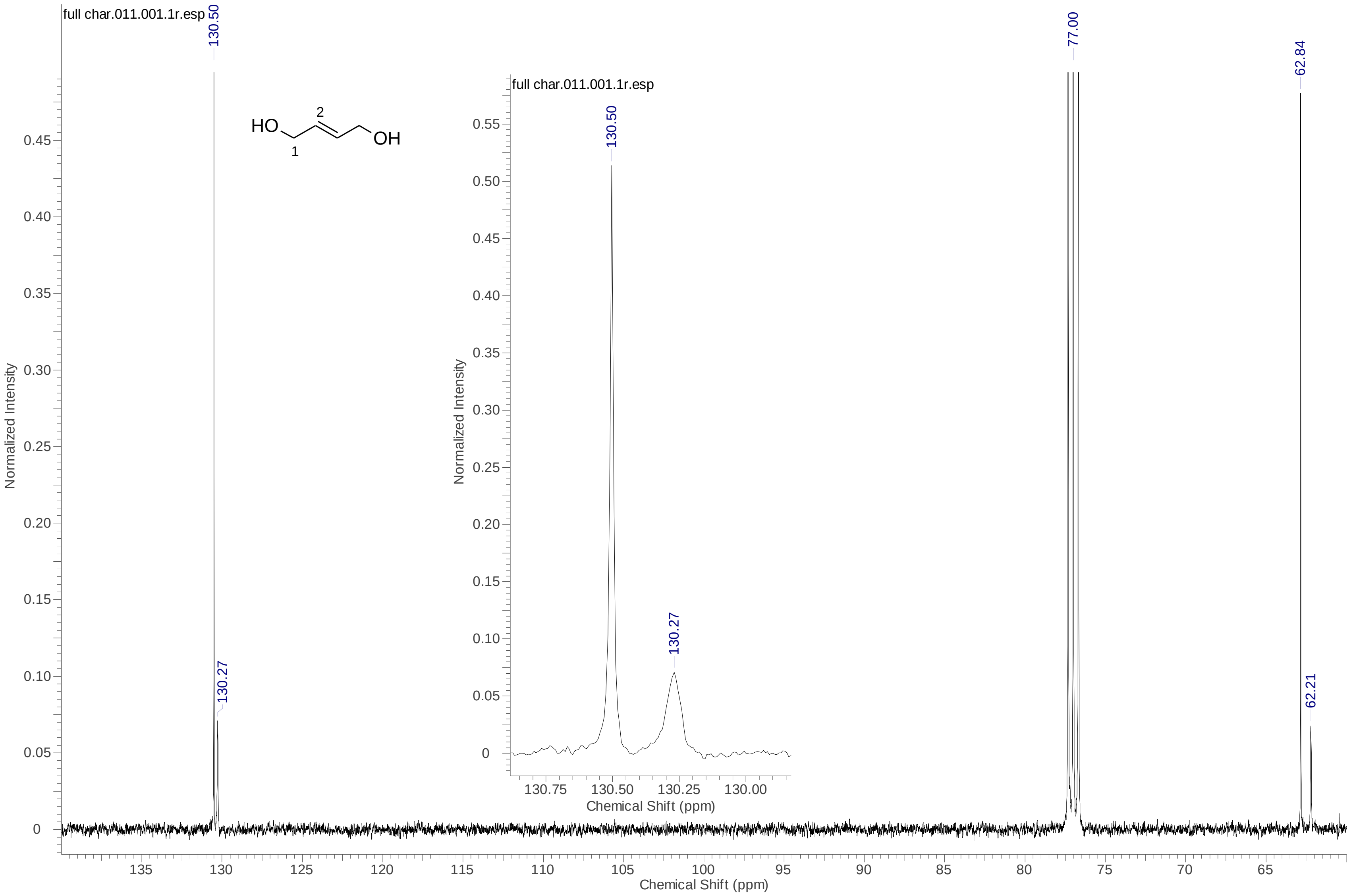

2.11 *(E)*-1,4-Dibenzyloxybut-2-ene (*trans*-14)

2.11.1 ^{1}H NMR (400 MHz, $CDCl_3$)

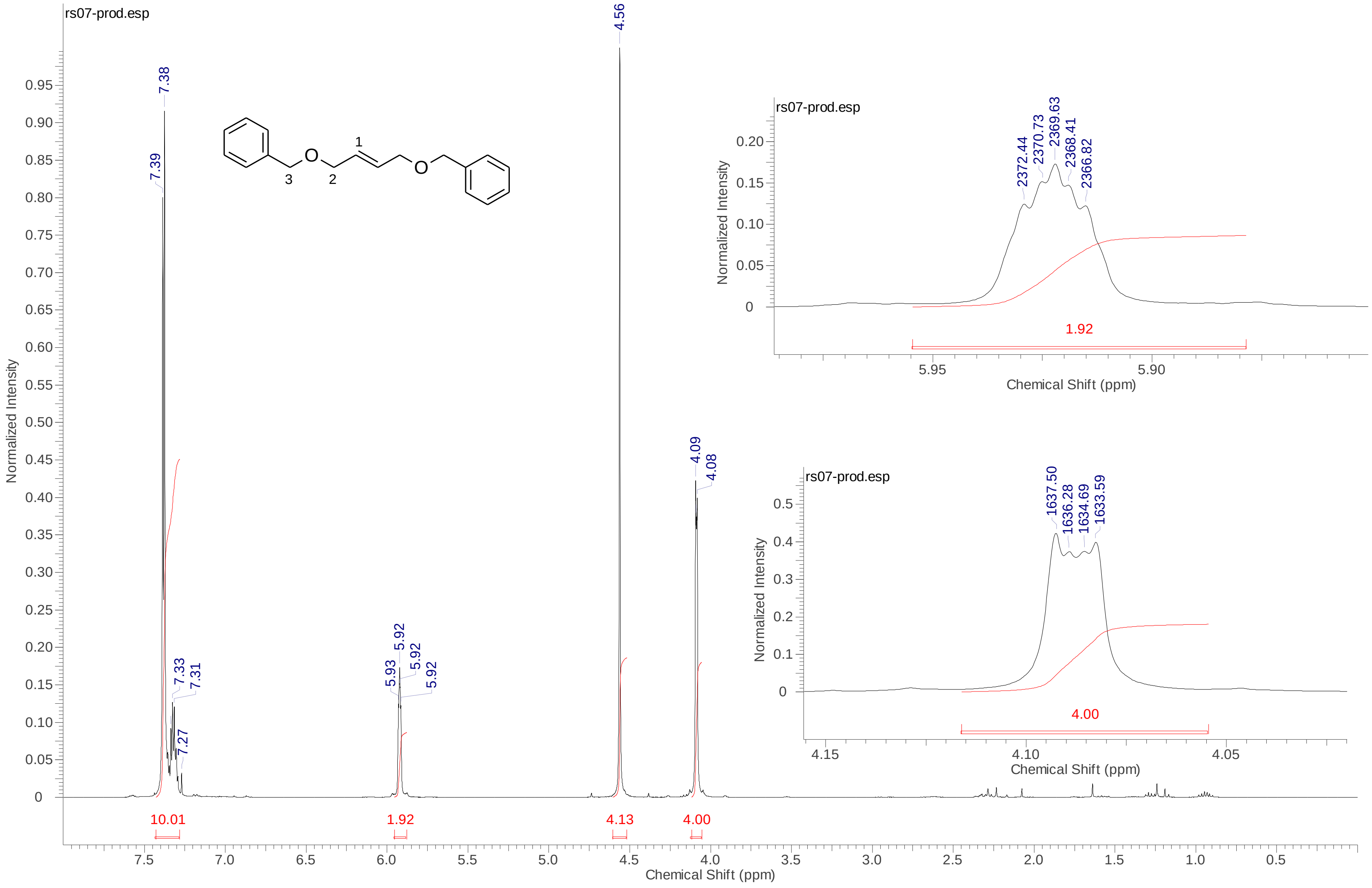

2.11.2 ^{13}C NMR (100 MHz, $CDCl_3$)

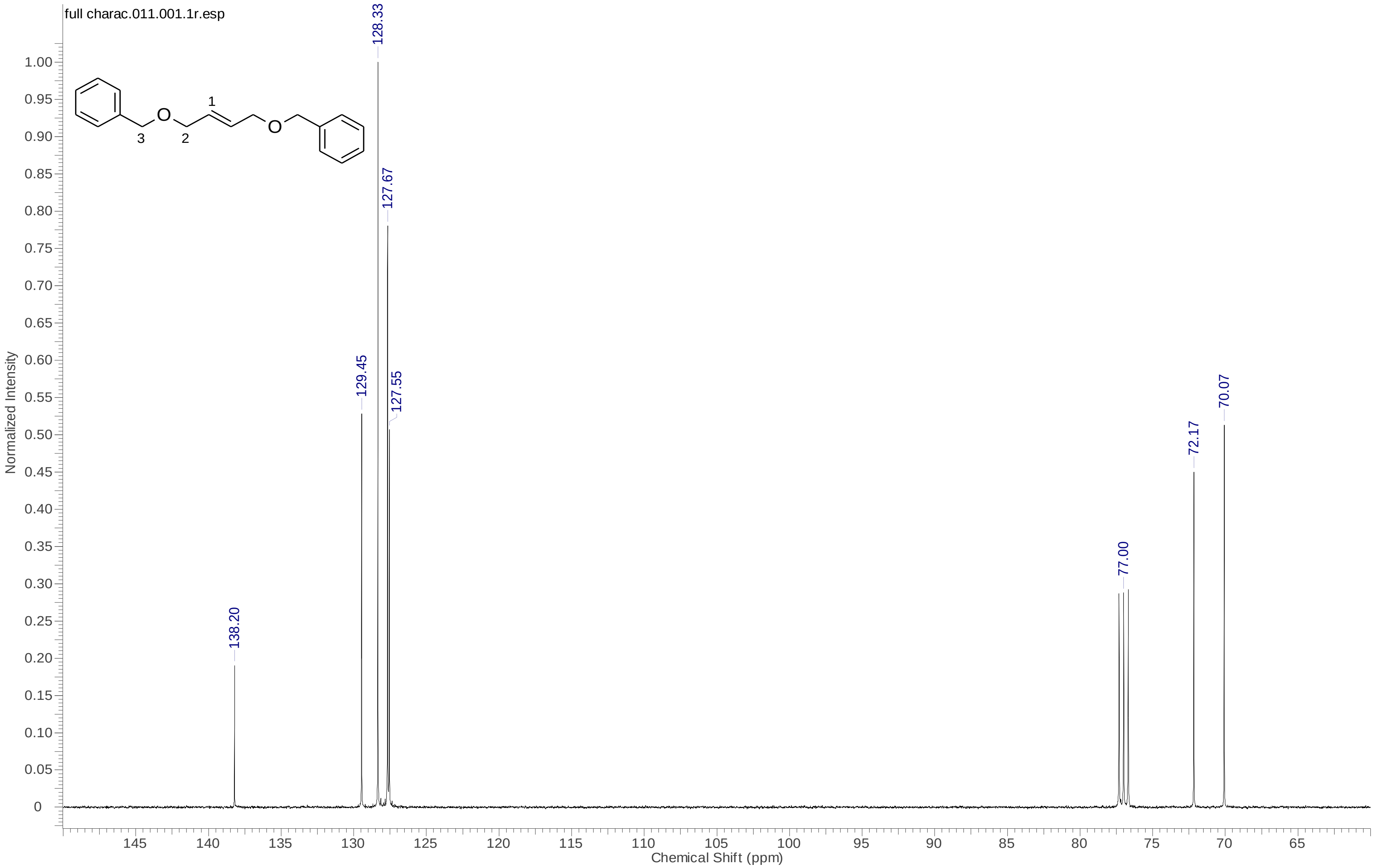

2.12 *trans*-1,2-Bis((benzyloxy)methyl)oxirane ((±)-*trans*-2)

2.12.1 ^{1}H NMR (400 MHz, $CDCl_3$)

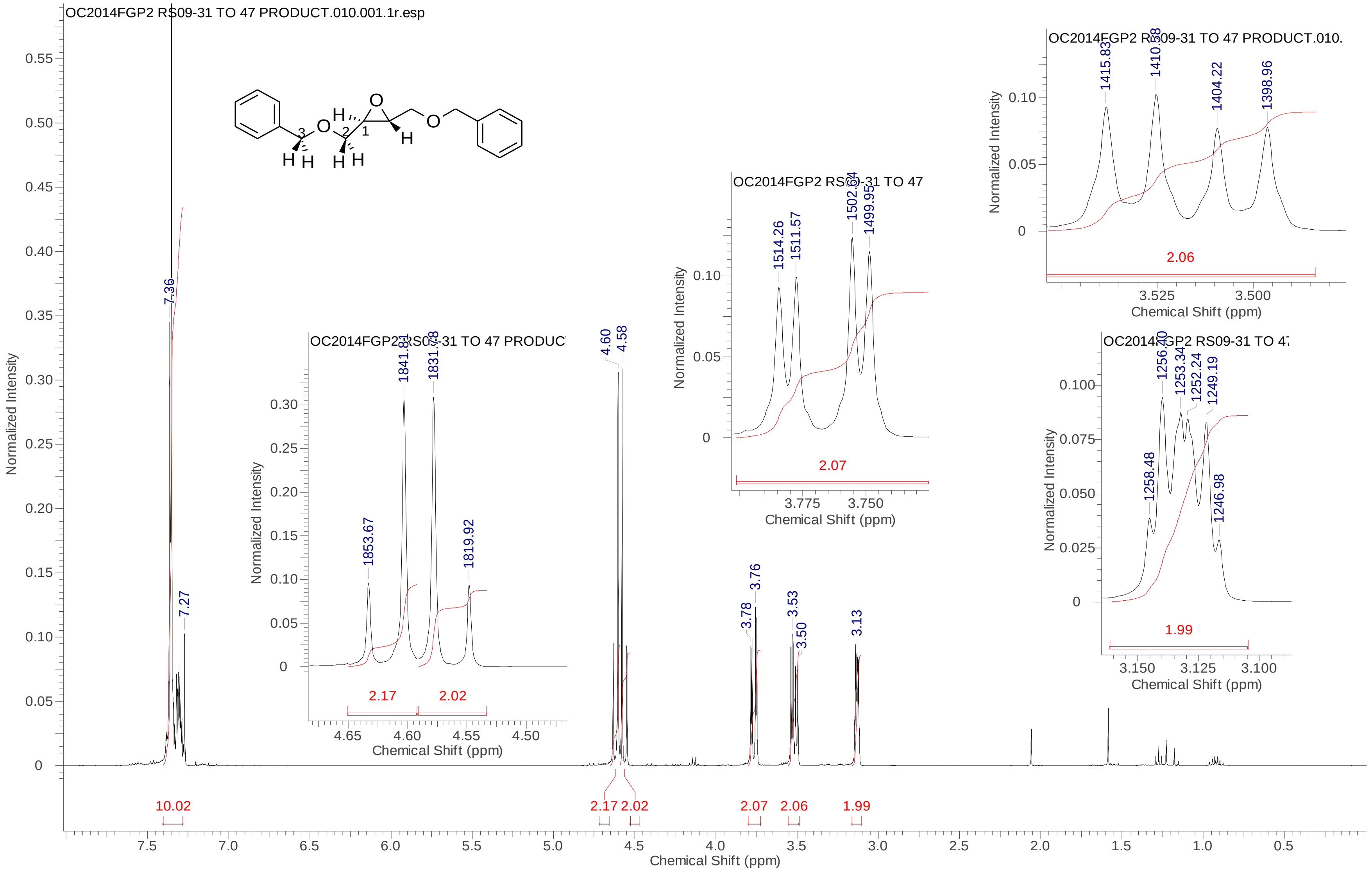

2.12.2 ^{13}C NMR (100 MHz, $CDCl_3$)

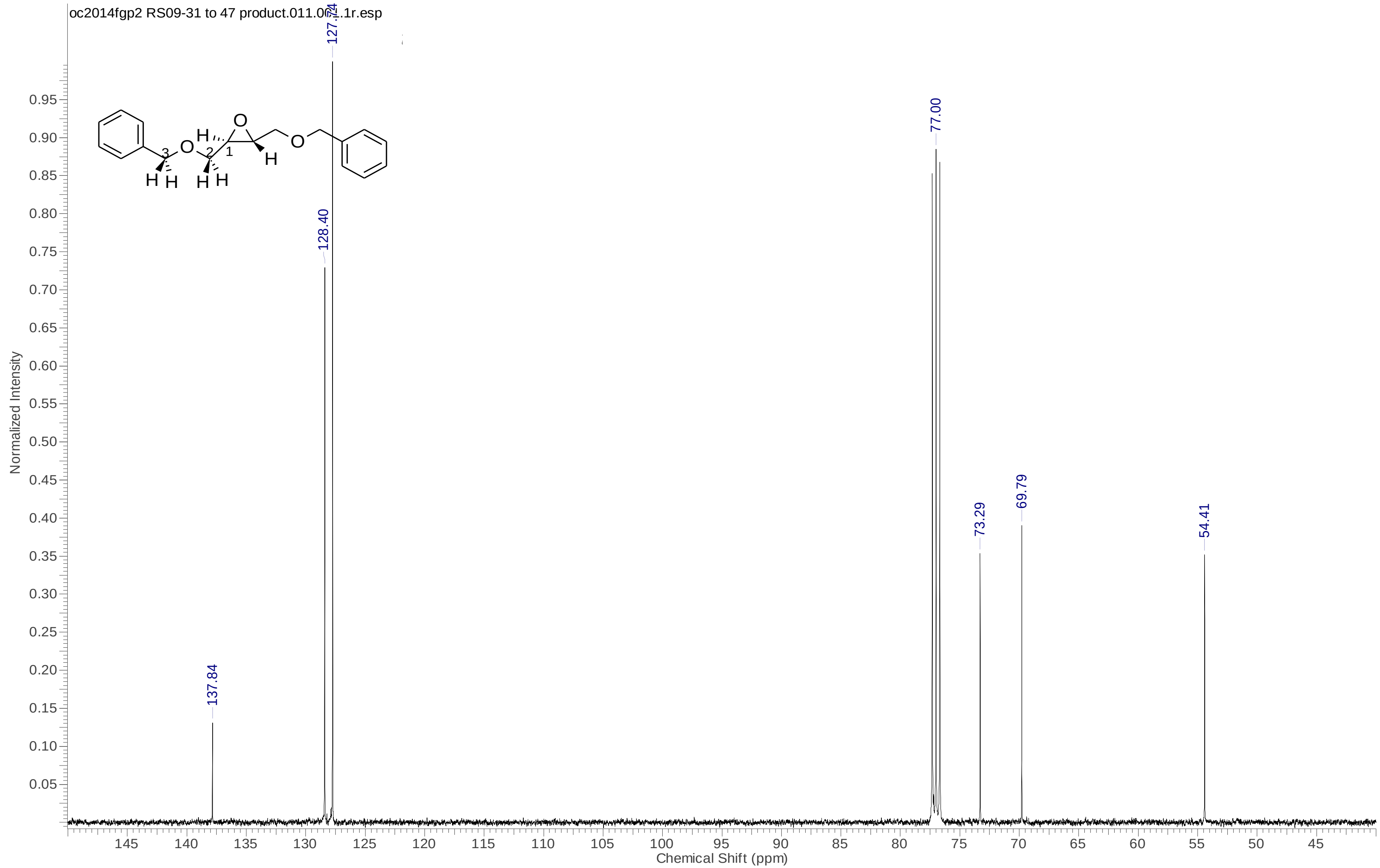

2.13 *anti*-1,4-Bis(benzyloxy)-3-fluorobutan-2-ol ((±)-*anti*-3)

2.13.1 ^{1}H NMR (500 MHz, $CDCl_3$)

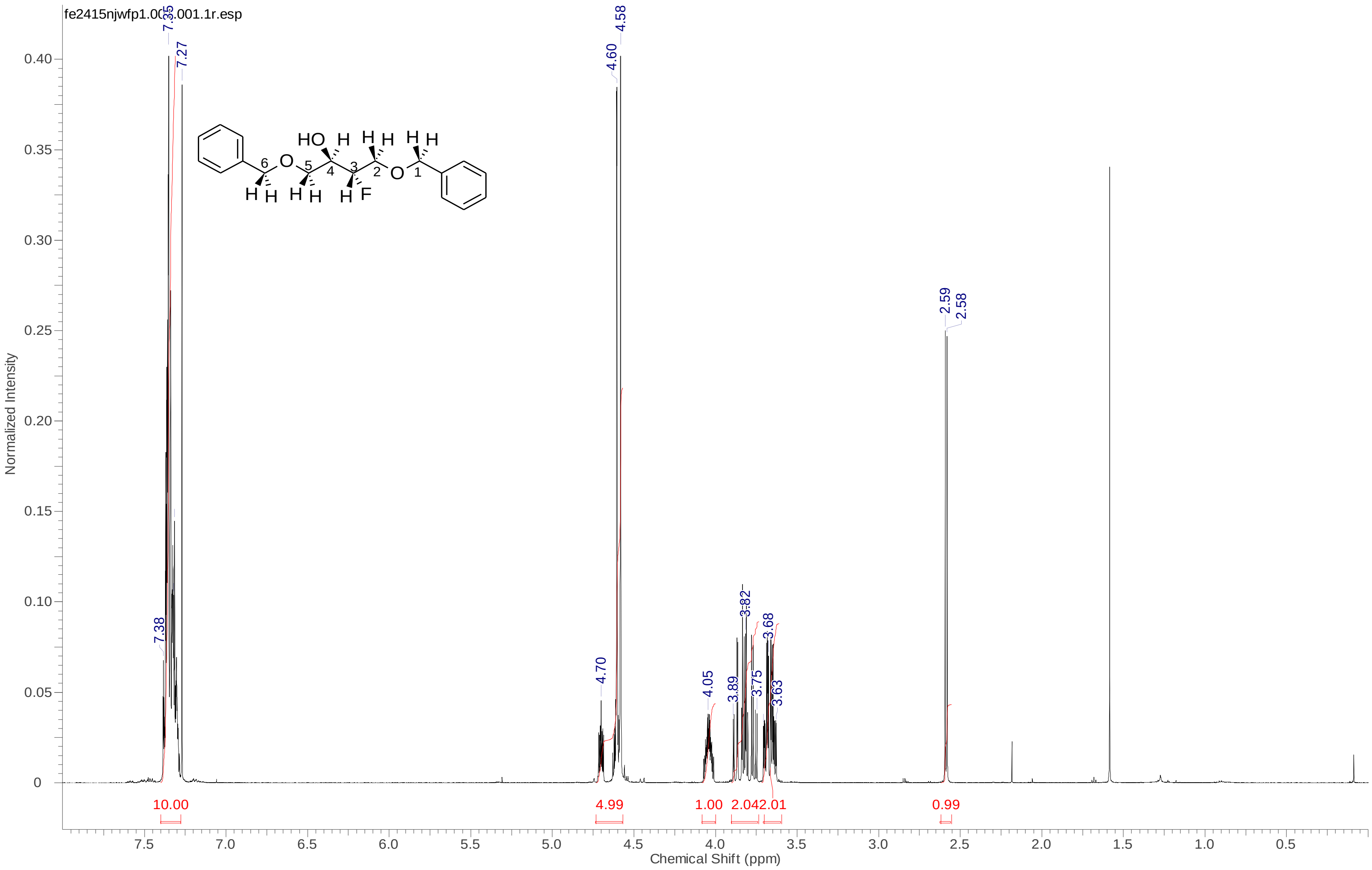

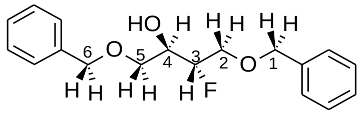
HO
H
H
H
H
H
6
O
5
4
3
2
O
1
H H
H H
H F

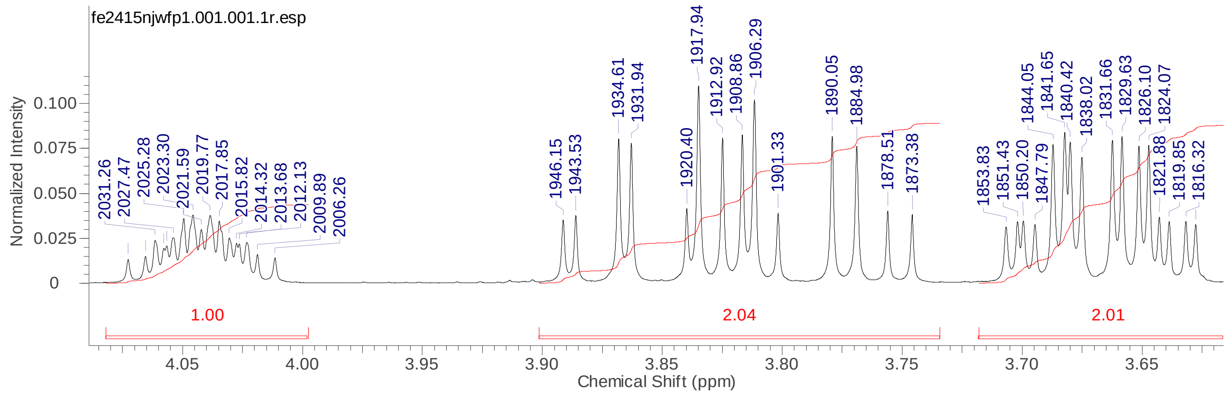
fe2415njwfp1.001.001.1r.esp
2031.26
2027.47
2025.28
2023.30
2021.59
2019.77
2017.85
2015.82
2014.32
2013.68
2012.13
2009.89
2006.26
1946.15
1943.53
1934.61
1931.94
1920.40
1917.94
1912.92
1908.86
1906.29
1901.33
1890.05
1884.98
1878.51
1873.38
1853.83
1851.43
1850.20
1847.79
1844.05
1841.65
1840.42
1838.02
1831.66
1829.63
1826.10
1824.07
1821.88
1819.85
1816.32
1.00
2.04
2.01
Normalized Intensity
Chemical Shift (ppm)

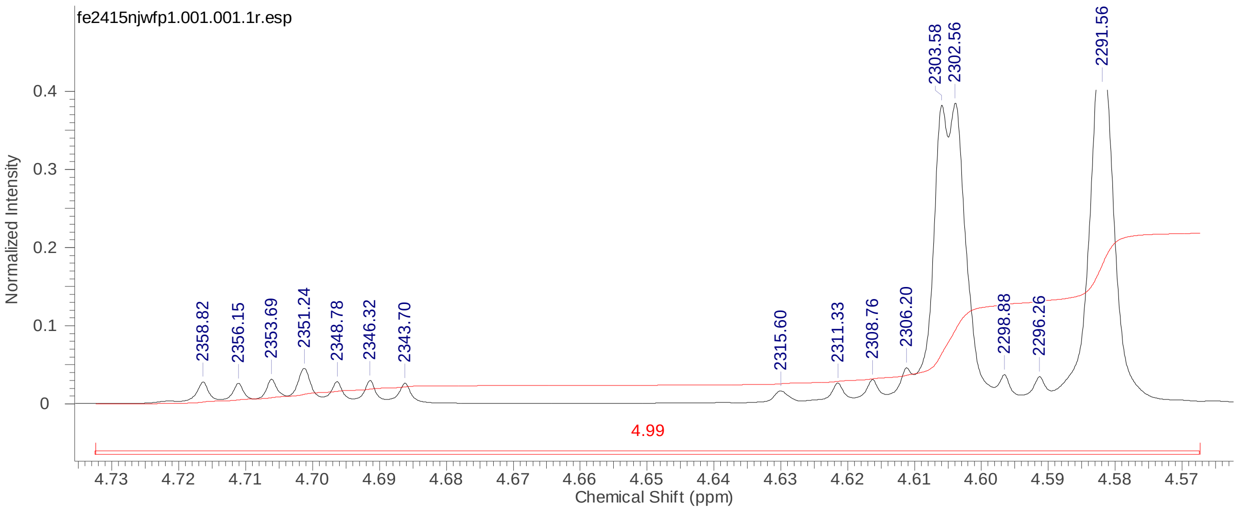
fe2415njwfp1.001.001.1r.esp
2358.82
2356.15
2353.69
2351.24
2348.78
2346.32
2343.70
2315.60
2311.33
2308.76
2306.20
2303.58
2302.56
2298.88
2296.26
2291.56
4.99
Normalized Intensity
Chemical Shift (ppm)

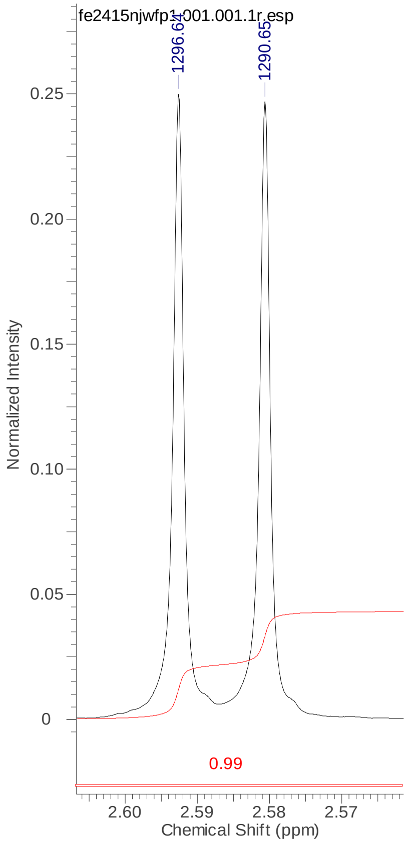
fe2415njwfp1.001.001.1r.esp
1296.63
1290.65
0.99
Normalized Intensity
Chemical Shift (ppm)

2.13.2 ^{13}C NMR (100 MHz, $CDCl_3$)

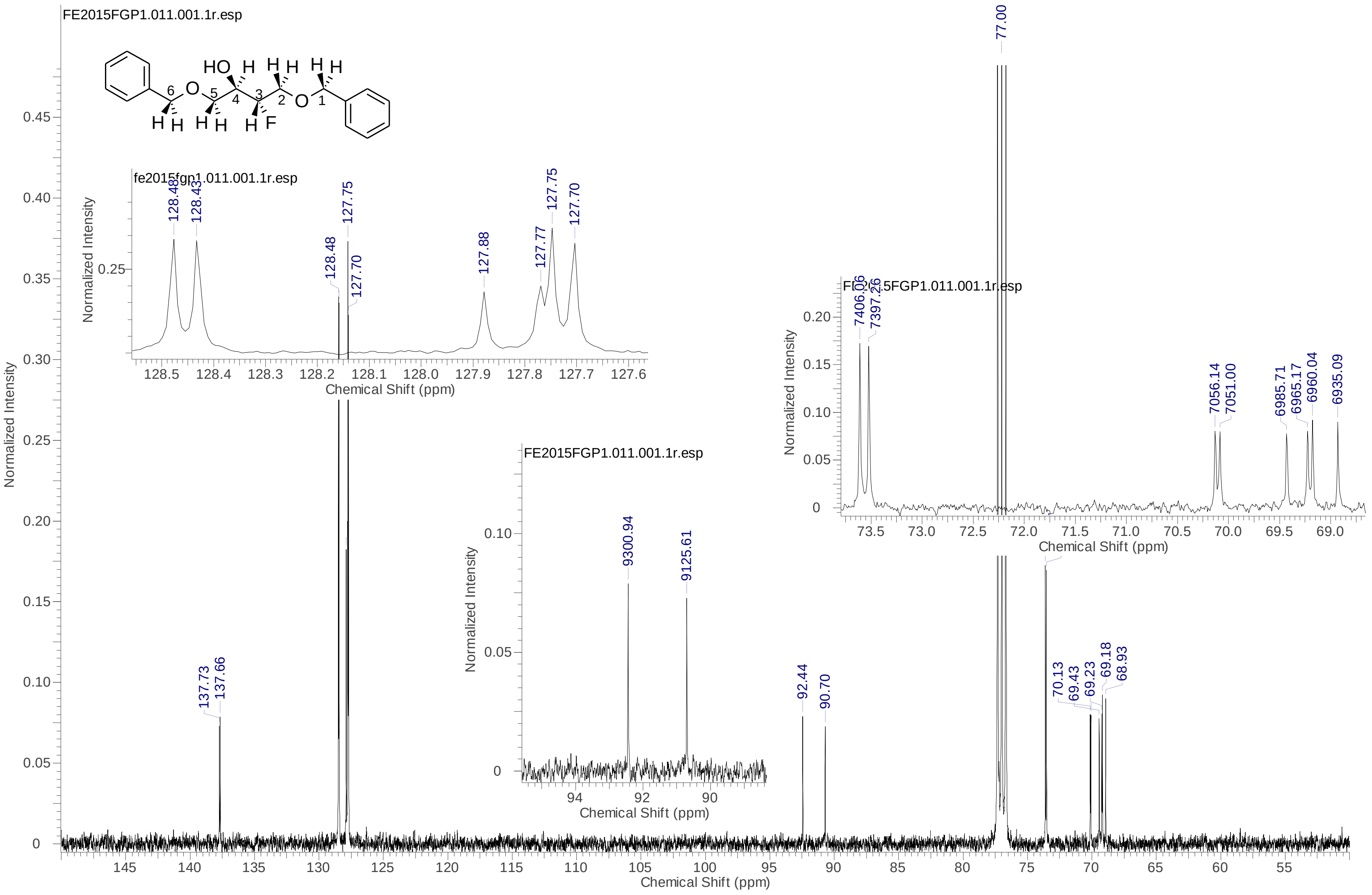

2.13.3 ^{19}F NMR (376 MHz, $CDCl_3$)

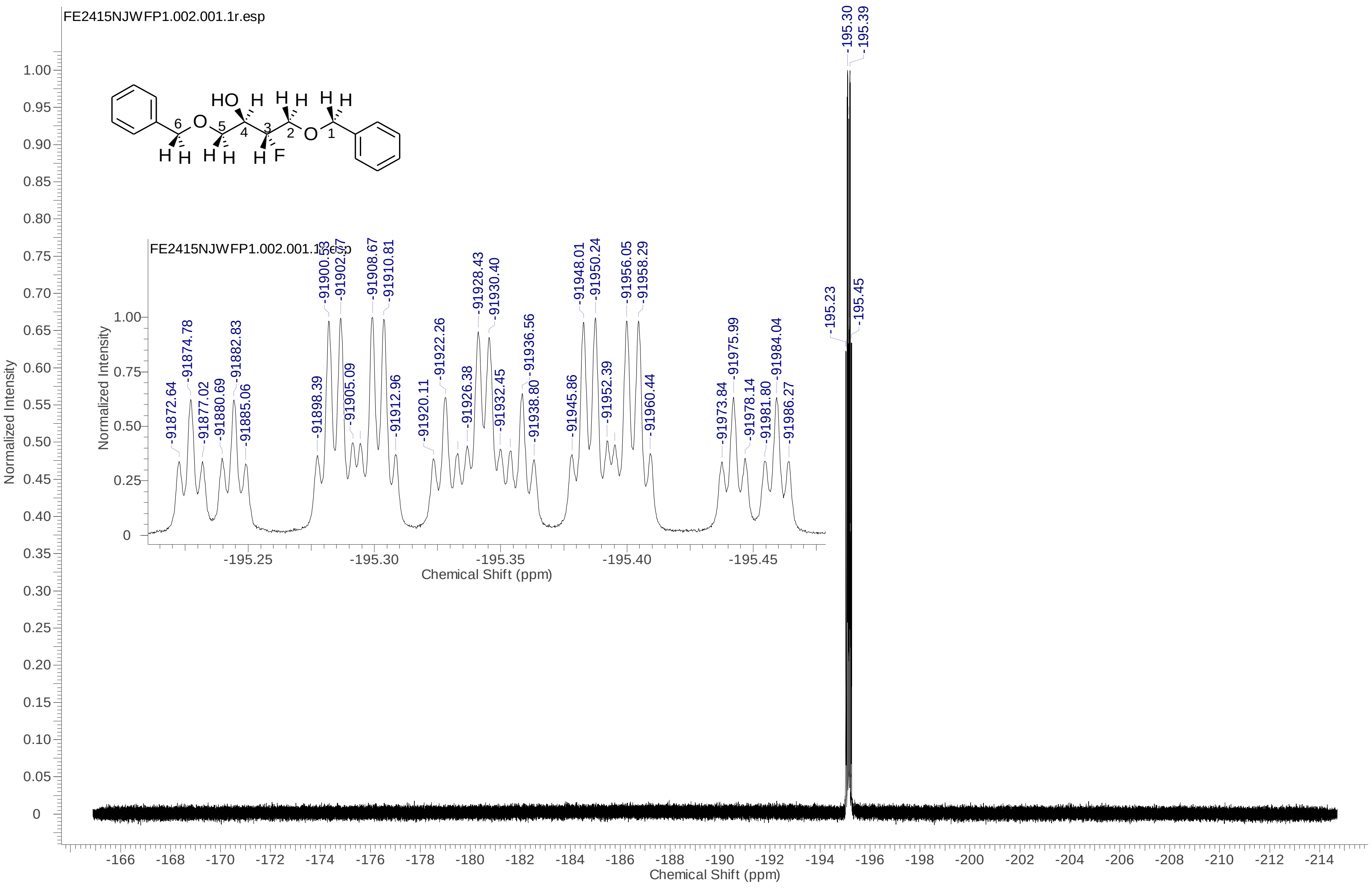

2.13.4 Diastereoselectivity of the epoxide opening

2.13.4.1 ^{1}H NMRs of the *cis*- and *trans*-epoxide starting material

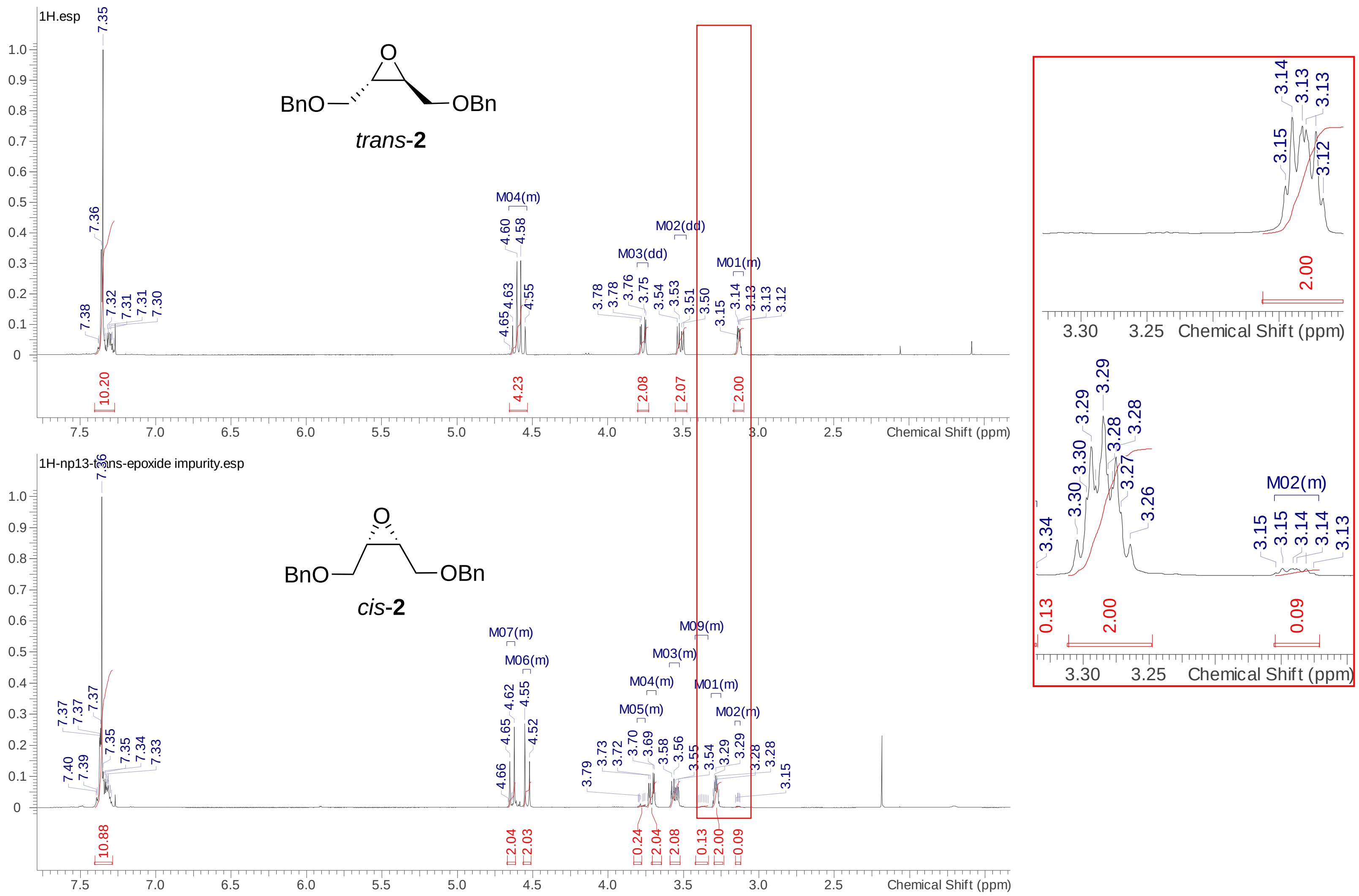

2.13.4.2 ^{19}F NMR of the fluorohydrin product

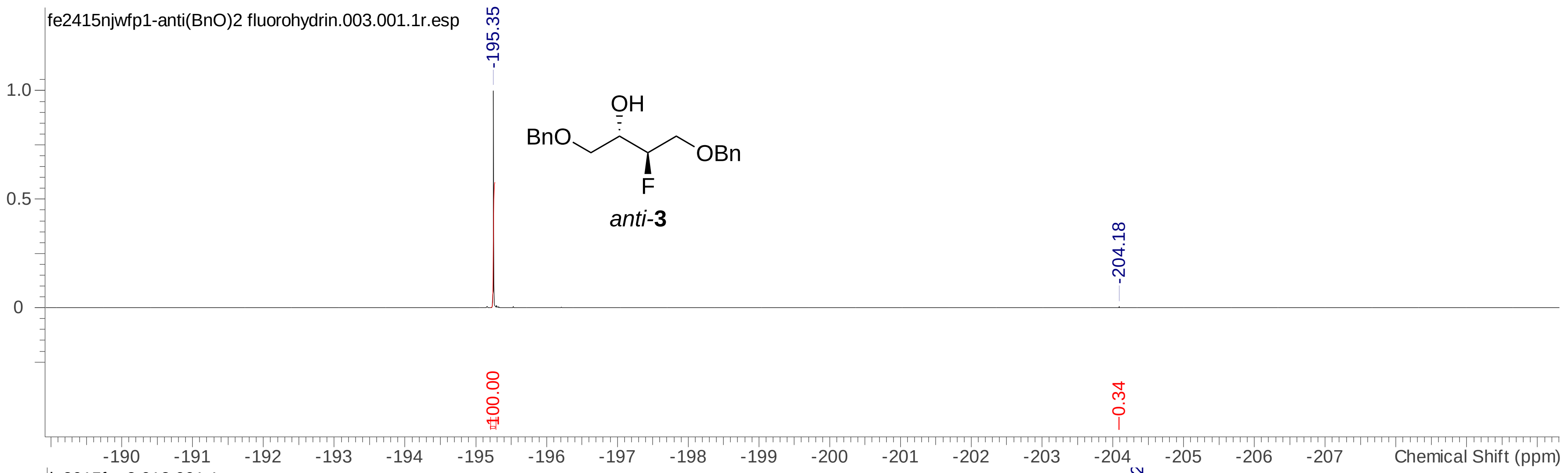

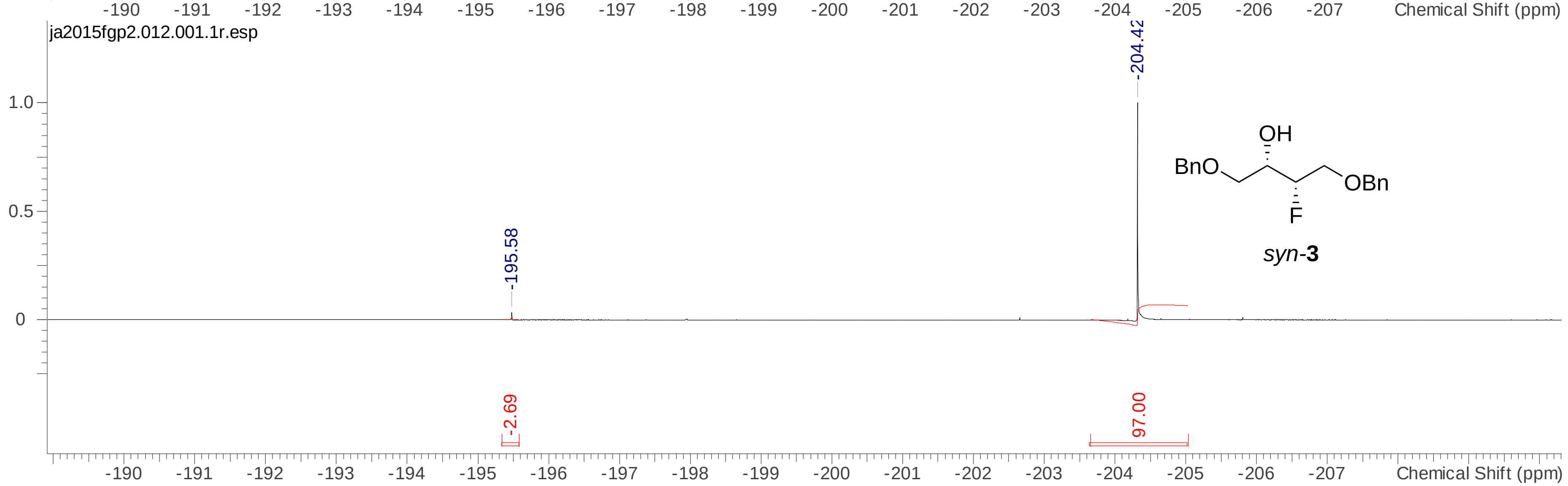

2.14 *syn*-1,4-Bis(benzyloxy)-2,3-difluorobutane ((±)-*syn*-4)

2.14.1 ^{1}H NMR (500 MHz, $CDCl_3$)

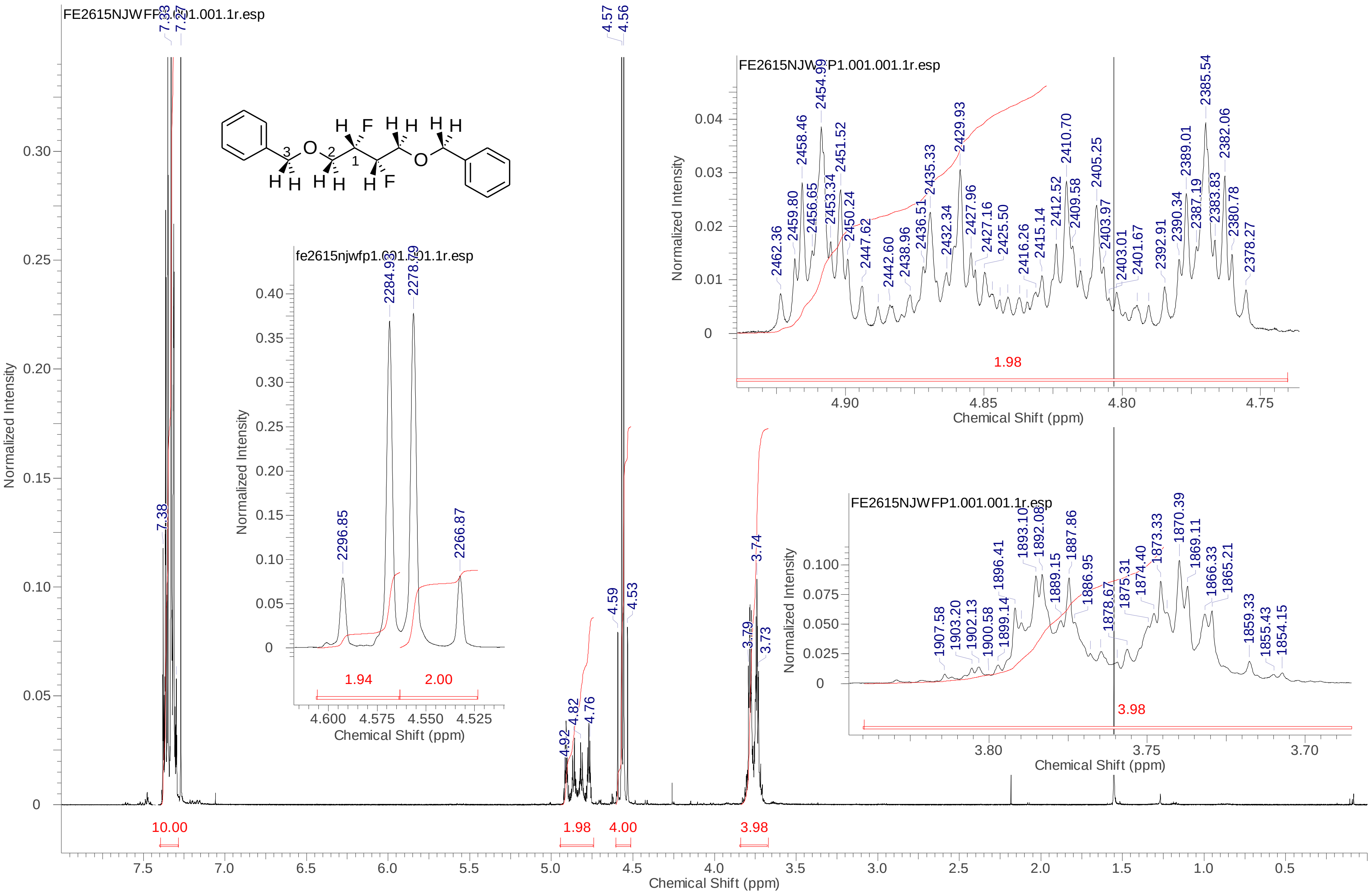

2.14.2 ^{13}C NMR (100 MHz, $CDCl_3$)

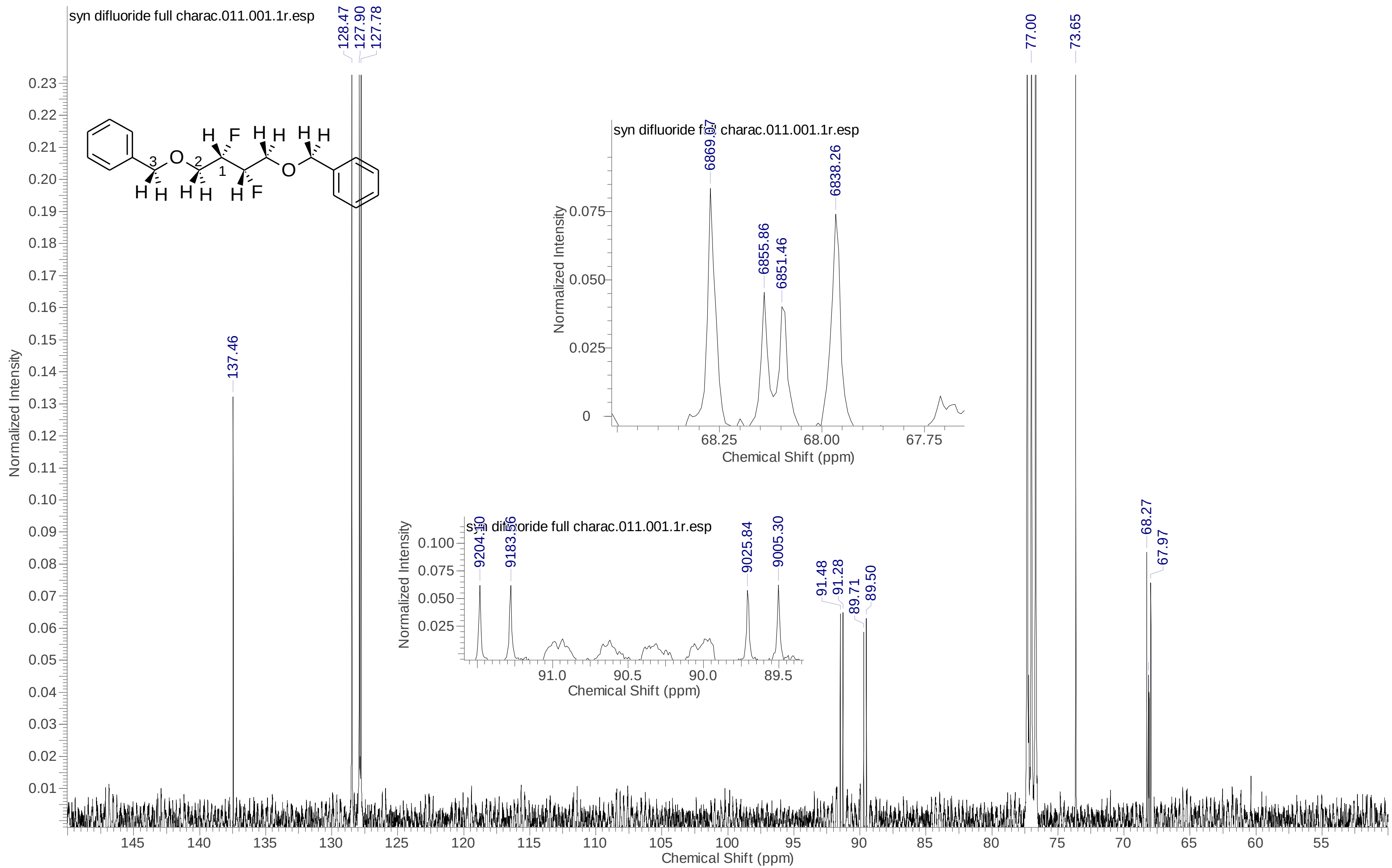

2.14.3 ^{19}F NMR (376 MHz, $CDCl_3$)

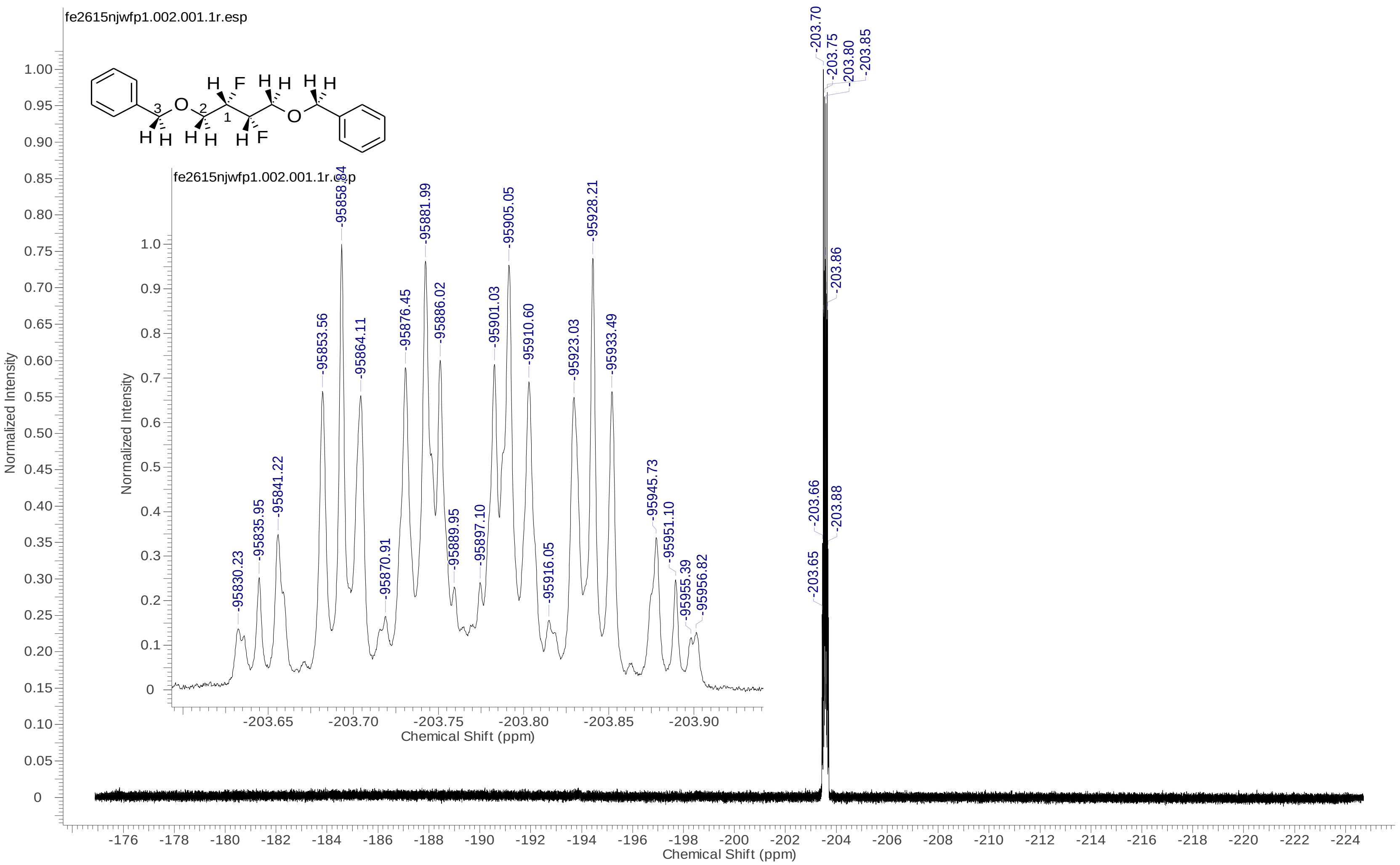

2.14.3.1 {^{1}H}^{19}F NMR (376 MHz, $CDCl_3$)

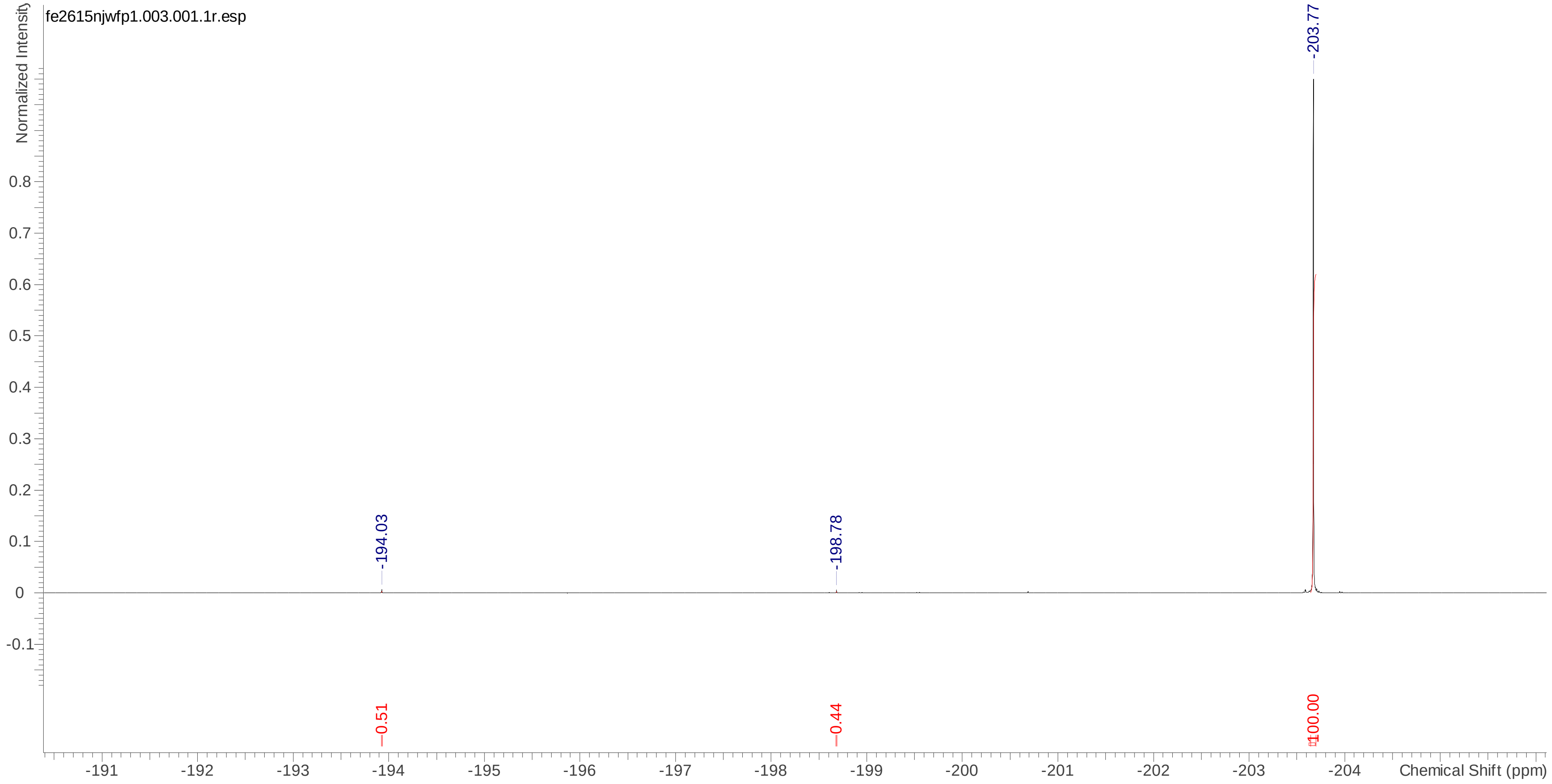

2.14.4 Diastereoselectivity of the DAST reaction

2.14.4.1 ^{19}F NMRs of the *syn*-fluorohydrin starting material, compared to that of the *anti*

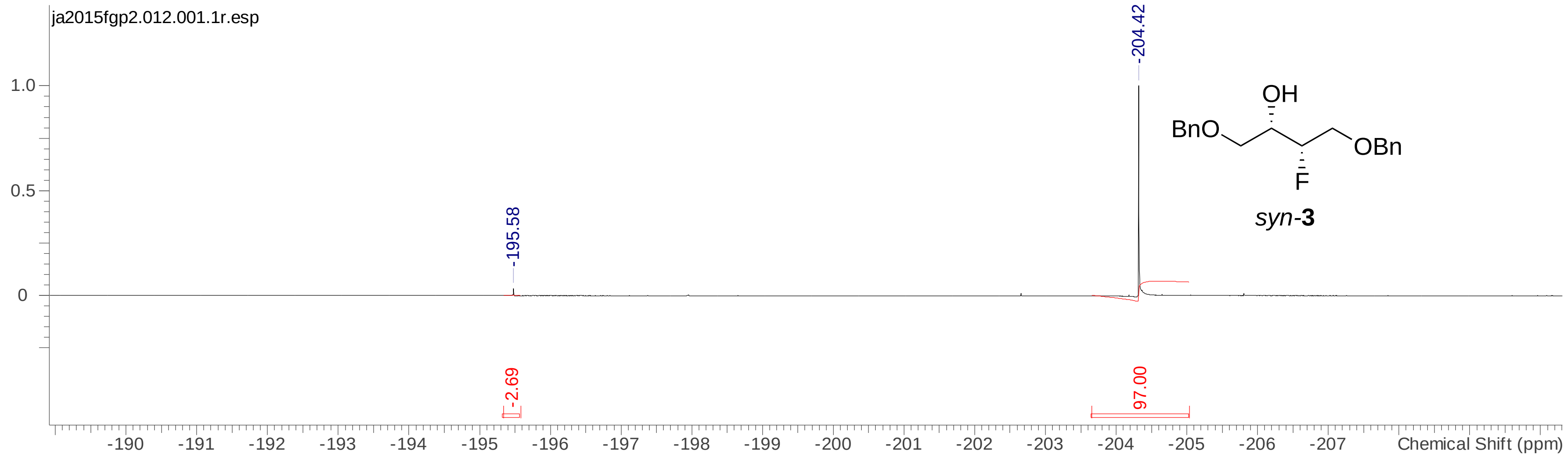

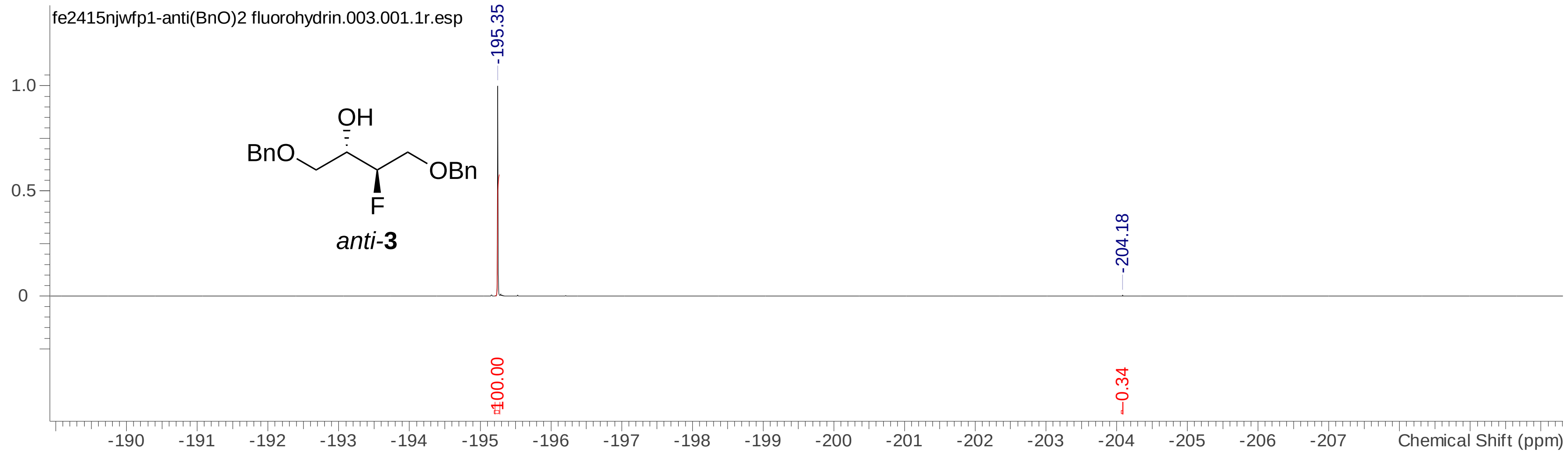

2.14.4.2 ^{19}F NMR of *anti*-**4**, compared to that of *syn*-**4**

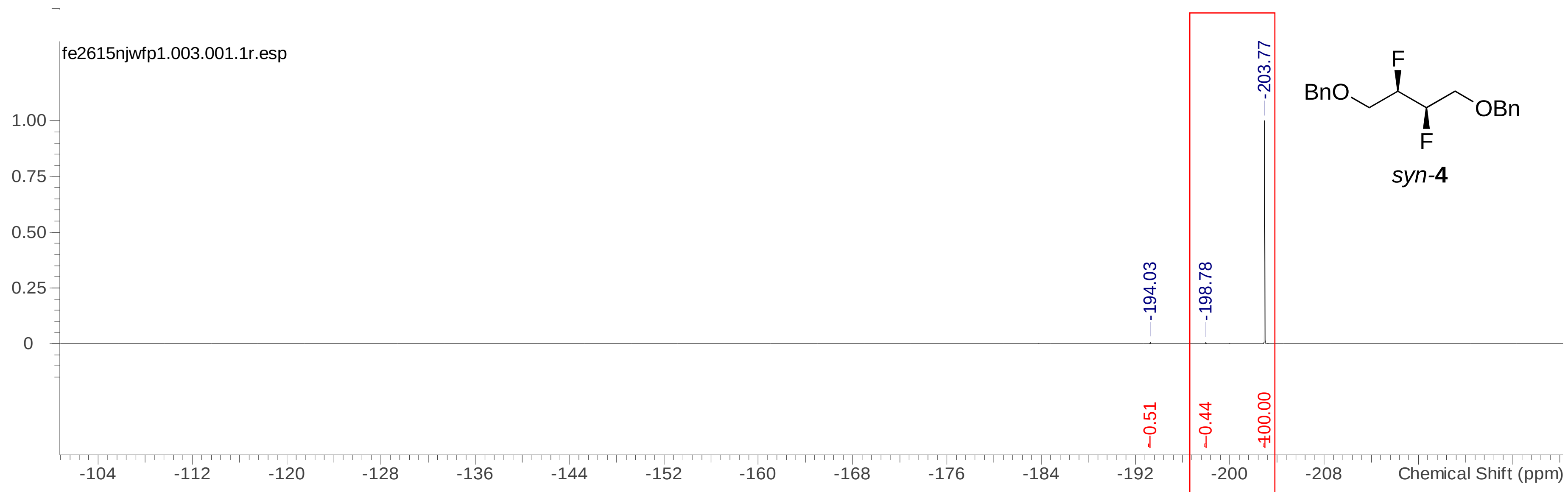

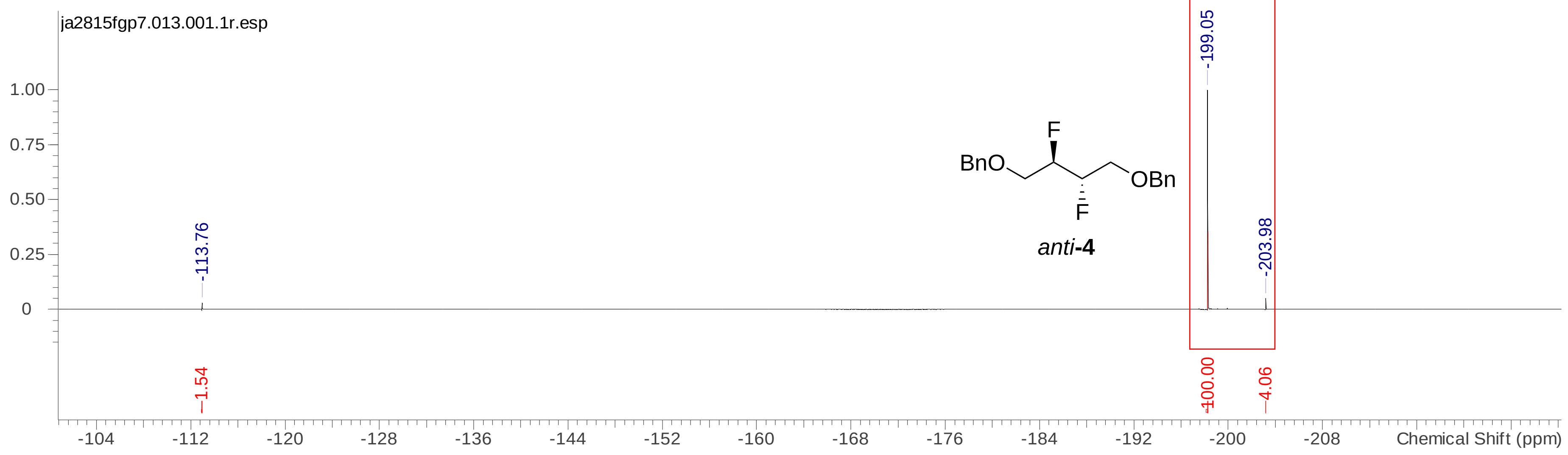

2.15 *syn*-2,3-Difluorobutane-1,4-diol ((±)-*syn*-5)

2.15.1 $^{1}H\{^{19}F\}$ NMR (500 MHz, $CDCl_3$)

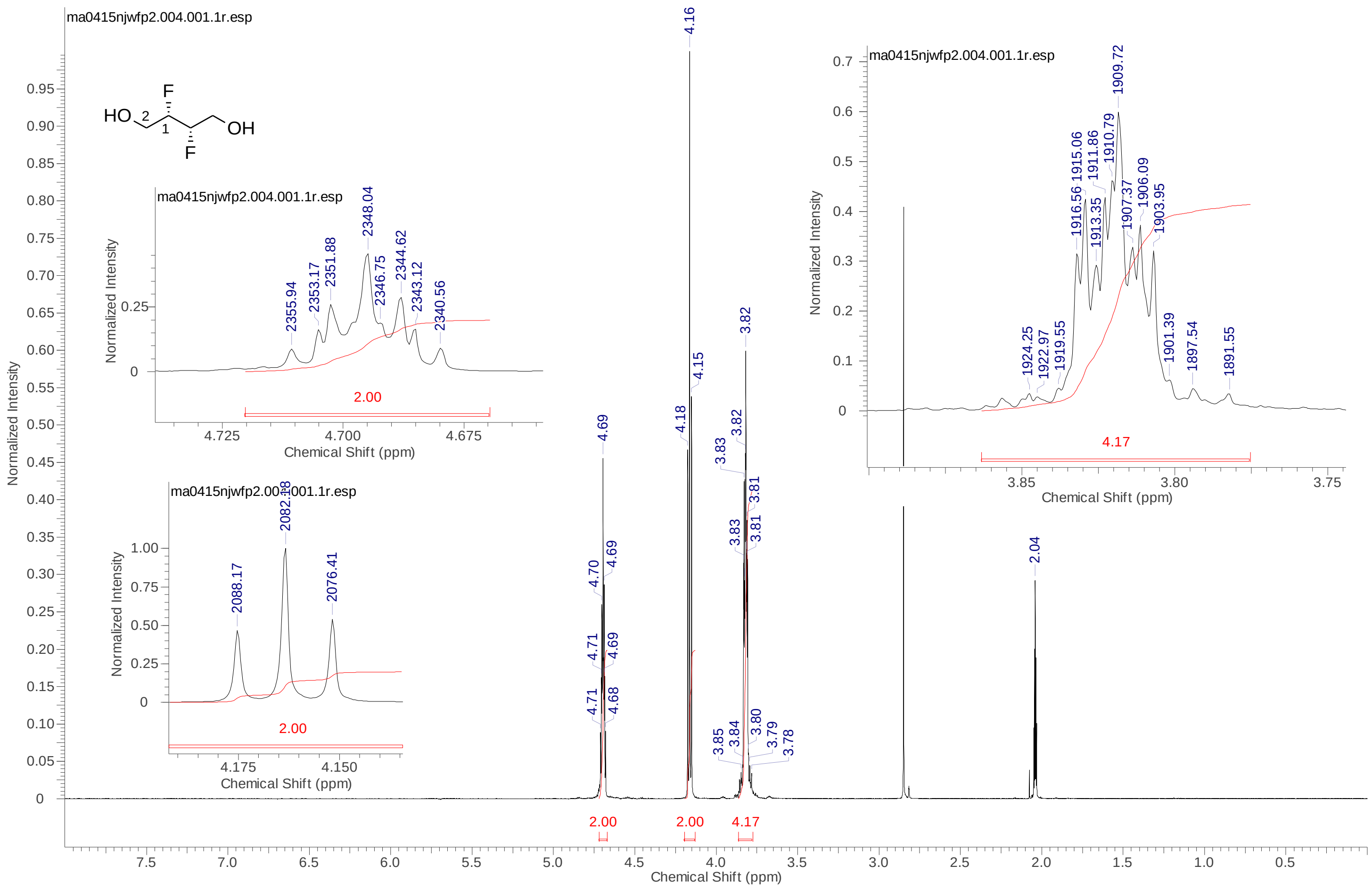

2.15.2 ^{13}C NMR (100 MHz, $CDCl_3$)

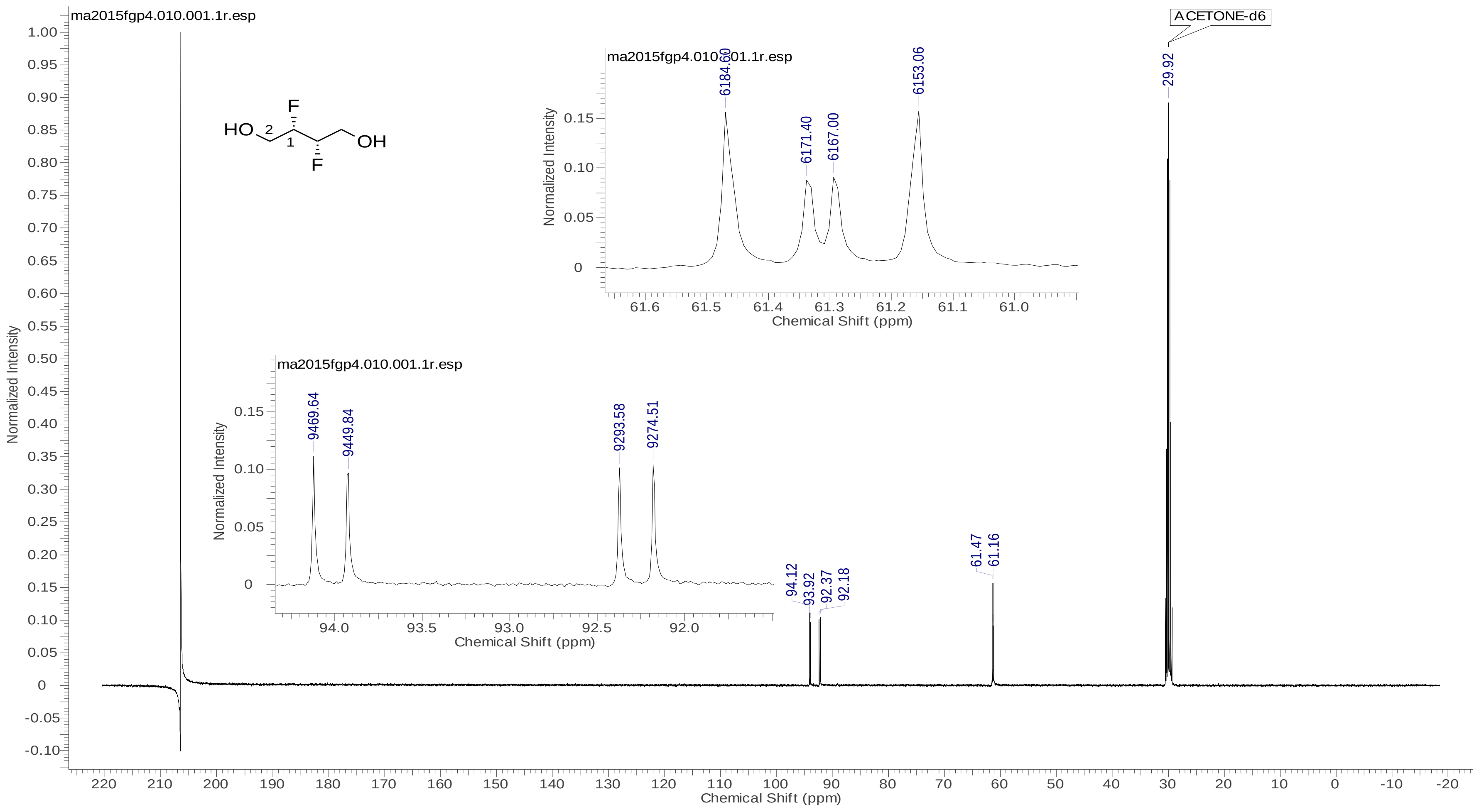

2.15.3 ^{19}F NMR (376 MHz, $CDCl_3$)

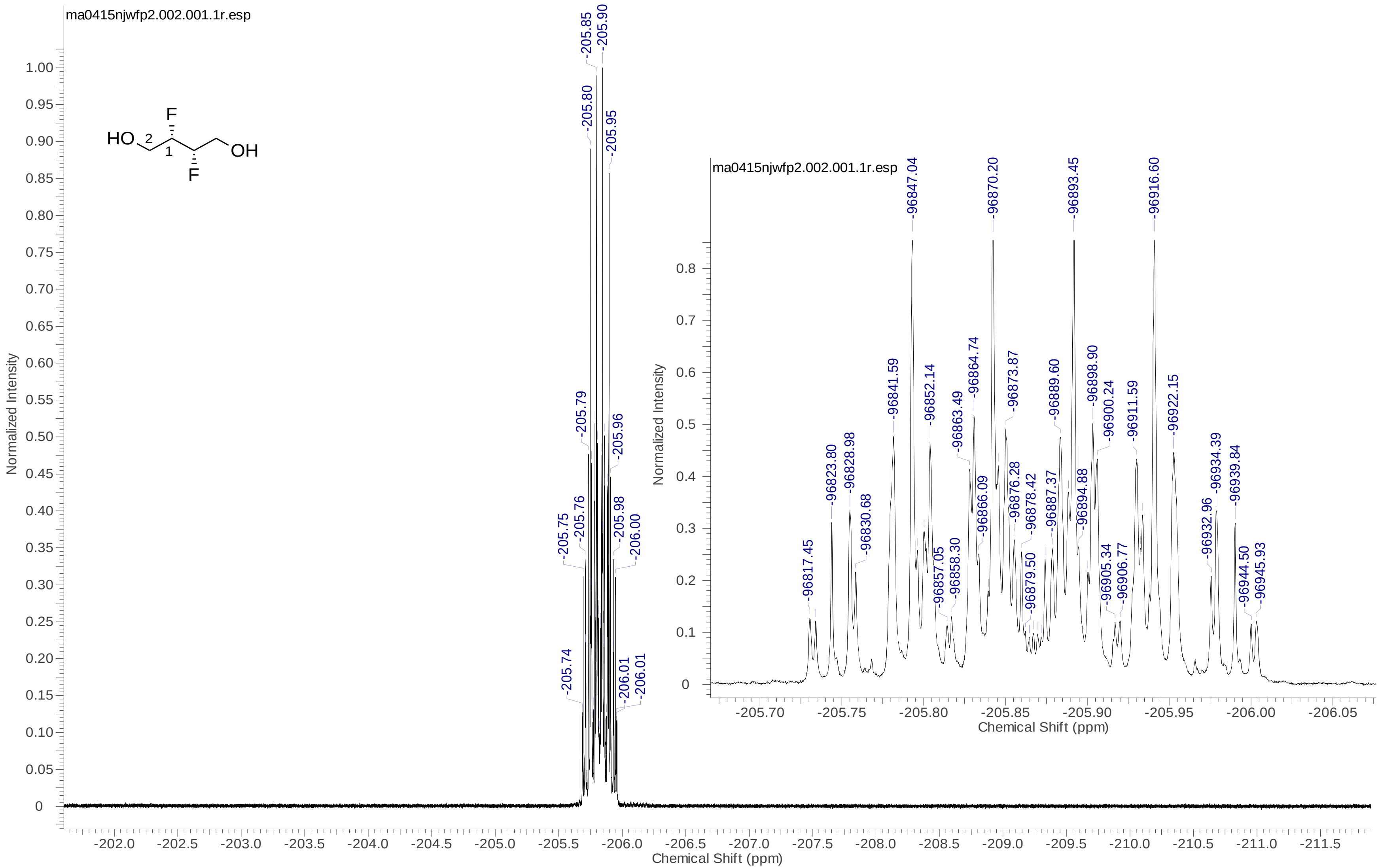

Supplement: File 1 — Experimental part and NMR spectra. [file Beilstein_J_Org_Chem-13-2883-s001.pdf]
